# CRISPR-Cas12a exploits R-loop asymmetry to form double-strand breaks

**DOI:** 10.7554/eLife.55143

**Published:** 2020-06-10

**Authors:** Joshua C Cofsky, Deepti Karandur, Carolyn J Huang, Isaac P Witte, John Kuriyan, Jennifer A Doudna

**Affiliations:** 1 Department of Molecular and Cell Biology, University of California, BerkeleyBerkeleyUnited States; 2 California Institute for Quantitative Biosciences (QB3), University of California, BerkeleyBerkeleyUnited States; 3 Howard Hughes Medical Institute, University of California, BerkeleyBerkeleyUnited States; 4 Department of Chemistry, University of California, BerkeleyBerkeleyUnited States; 5 MBIB Division, Lawrence Berkeley National LaboratoryBerkeleyUnited States; 6 Innovative Genomics Institute, University of California, BerkeleyBerkeleyUnited States; 7 Gladstone Institutes, University of California, San FranciscoSan FranciscoUnited States; Johns Hopkins University School of MedicineUnited States; Department of Biochemistry and Molecular Biology, Johns Hopkins Bloomberg School of Public HealthUnited States

**Keywords:** CRISPR, genome editing, RNA, deoxyribonuclease, R-loop, *E. coli*

## Abstract

Type V CRISPR-Cas interference proteins use a single RuvC active site to make RNA-guided breaks in double-stranded DNA substrates, an activity essential for both bacterial immunity and genome editing. The best-studied of these enzymes, Cas12a, initiates DNA cutting by forming a 20-nucleotide R-loop in which the guide RNA displaces one strand of a double-helical DNA substrate, positioning the DNase active site for first-strand cleavage. However, crystal structures and biochemical data have not explained how the second strand is cut to complete the double-strand break. Here, we detect intrinsic instability in DNA flanking the RNA-3′ side of R-loops, which Cas12a can exploit to expose second-strand DNA for cutting. Interestingly, DNA flanking the RNA-5′ side of R-loops is not intrinsically unstable. This asymmetry in R-loop structure may explain the uniformity of guide RNA architecture and the single-active-site cleavage mechanism that are fundamental features of all type V CRISPR-Cas systems.

## Introduction

**Figure 1.**
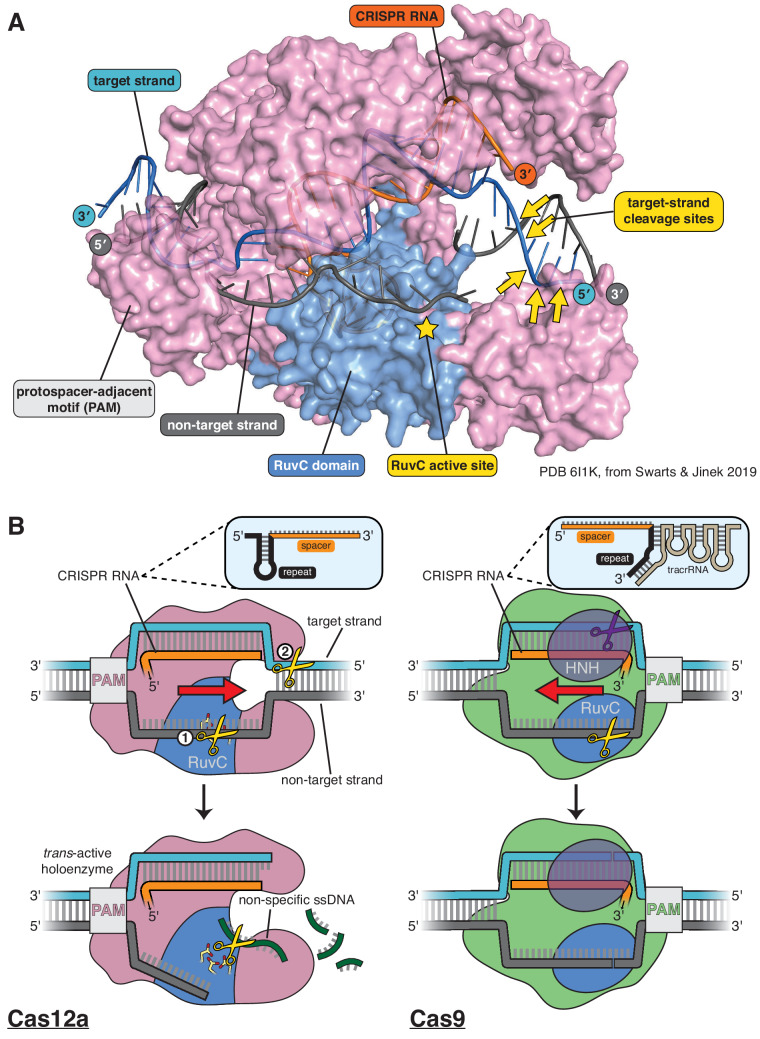
Structure of Cas12a and comparison of its DNA cleavage pathway to that of Cas9. (**A**) Crystal structure of the DNA-bound Cas12a interference complex from *Francisella novicida* (FnCas12a, PDB 61IK) (Swarts and Jinek, 2019). While the protein ortholog used for most experiments in this manuscript is from *Acidaminoccus species* (AsCas12a,~40% identity to FnCas12a), the FnCas12a crystal structure shown here represents the most complete structure of such a complex to date, most notably with respect to the DNA at the target-strand cleavage sites. We did not perform any experiments with the particular DNA sequence used by Swarts and Jinek in crystallization, so the scissile phosphodiesters indicated were determined for a different sequence (see Appendix 2—figure 1, Appendix 2—figure 1—figure supplement 6) and superimposed onto the structural model according to their distance from the PAM (in terms of number of nucleotides). The discontinuity modeled into the non-target strand corresponds to positions of weak electron density in the crystal structure, which could have been due to some combination of disorder of the (intact) intervening tract and/or *in crystallo* hydrolysis and dissociation of the intervening tract. (**B**) For Cas12a, successful R-loop formation results in activation of the RuvC DNase active site to cleave three classes of DNA substrates (yellow scissors): the non-target strand (in *cis*), the target strand (in *cis*), and non-specific ssDNA (in *trans*). Circled numbers indicate the required order of *cis* strand cleavage; three conserved active site carboxylates of the RuvC DNase are shown in yellow and red; ‘PAM’ indicates the protospacer-adjacent motif; red arrow indicates the direction in which the R-loop is opened. Cas9 contains two DNase domains: the RuvC domain cleaves the non-target strand, and the HNH domain cleaves the target strand.

CRISPR-Cas systems (clustered regularly interspaced short palindromic repeats, CRISPR-associated proteins) provide antiviral immunity to prokaryotes through the RNA-guided nuclease activity of enzymes including Cas9 and Cas12a (Barrangou et al., 2007; Jinek et al., 2012; Zetsche et al., 2015; Makarova et al., 2015), which are widely used for programmable genome editing (Pickar-Oliver and Gersbach, 2019). Both Cas9 and Cas12a use CRISPR RNA (crRNA) to recognize matching double-stranded DNA (dsDNA) sequences by forming an R-loop structure in which 20 nucleotides (nts) of the crRNA (the crRNA ‘spacer’) base pair with one strand of the target DNA (Figure 1A,B; Jinek et al., 2012; Jiang et al., 2016; Zetsche et al., 2015; Swarts et al., 2017). In addition, both protein families must bind to a protospacer-adjacent motif (PAM), a short DNA sequence next to the crRNA-complementary sequence, to initiate R-loop formation (Bolotin et al., 2005; Mojica et al., 2009; Sternberg et al., 2014; Singh et al., 2018).

Despite their functional similarities, Cas9 and Cas12a evolved independently (Koonin et al., 2017) and use distinct mechanisms of DNA cleavage. Cas9 employs two active sites to generate a blunt DNA double-strand break near the PAM (Jinek et al., 2012; Gasiunas et al., 2012). In contrast, Cas12a uses a single active site to make staggered cuts distant from the PAM, and the same active site can cleave free single-stranded DNA (ssDNA) non-specifically once the enzyme has been activated by specific target binding (Figure 1B; Zetsche et al., 2015; Swarts et al., 2017; Chen et al., 2018). Additionally, the position of the 20-nucleotide spacer sequence in Cas12a crRNAs is opposite to that in Cas9 crRNAs (3′ end versus 5′ end, respectively) (Zetsche et al., 2015; Deltcheva et al., 2011), and consequently, R-loop formation by each enzyme family occurs with opposing directionality (Figure 1B).

Cas12a belongs to the type V family of CRISPR effector proteins (whose names each begin with ‘Cas12’), a classification defined by the presence of a single RuvC DNase domain (Koonin et al., 2017). Notably, although this classification is not strictly phylogenetic—the ‘family’ is actually a paraphyletic assembly of several evolutionarily independent protein lineages (Koonin et al., 2017; Makarova et al., 2020)—a second shared feature of all Cas12 interference complexes is the previously mentioned spacer-3′ crRNA architecture. However, the reason for this evolutionary convergence is unknown. Furthermore, while structures of DNA-bound Cas12a are known (Yamano et al., 2016; Gao et al., 2016; Swarts et al., 2017; Stella et al., 2017; Stella et al., 2018; Swarts and Jinek, 2019) and an obligatory order of strand cleavage has been suggested biochemically (Swarts and Jinek, 2019), the mechanism by which type V CRISPR enzymes form double-strand breaks using one active site remains unclear.

The RuvC domain of Cas12a exhibits stringent specificity for single-stranded substrates when activated for cleavage of free DNA in *trans* (Appendix 1) (Chen et al., 2018), suggesting that substrates cleaved in *cis* (i.e., the two DNA strands of a protein-bound R-loop) are also single-stranded during each of their respective cleavage events. Consistent with this substrate preference, in the most complete crystal structures of the Cas12a-bound R-loop, displacement of the non-target strand (NTS) of the DNA allows its association with the RuvC active site as a single strand (Swarts et al., 2017; Swarts and Jinek, 2019). This conformation likely permits initial non-target-strand cleavage, which is a prerequisite of DNA target-strand (TS) cutting (Figure 1B; Swarts and Jinek, 2019).

However, in these same crystal structures, the target-strand cleavage site is located within an ordered DNA duplex outside the R-loop,~25 Å away from the RuvC active site and inverted with respect to the most probable catalytic orientation (Figure 1A; Swarts et al., 2017; Swarts and Jinek, 2019). To satisfy these geometric constraints and the RuvC substrate preference, the target strand likely needs to separate from the non-target strand and bend, accessing a conformation evoked by some structures of the type V CRISPR-Cas enzymes Cas12b (C2c1) and Cas12e (CasX) (Yang et al., 2016; Liu et al., 2019). It has been hypothesized that contortion of the DNA substrate enables Cas12a (and other Cas12 family members) to cleave the target strand and complete its double-strand break (Jeon et al., 2018; Zhang et al., 2019), but it is unknown what enables this contortion.

We show here that Cas12a cleaves the target strand within a tract of DNA that is destabilized by the adjacent R-loop. Using chemical and fluorescent probes to investigate DNA conformation in protein-bound and protein-free R-loops in solution, we find that DNA flanking the RNA-3′ side of the R-loop exhibits signatures of single-strandedness, despite this region’s potential for complete base pairing. The location of this DNA distortion controls the location of RuvC-catalyzed target-strand cleavage, suggesting that Cas12a exploits local duplex instability to complete its double-strand break. This cleavage mechanism is likely shared by other DNA-targeting CRISPR-Cas12 systems, which all use a single RuvC active site to cut the target strand within the DNA tract flanking the RNA-3′ side of the R-loop (Yang et al., 2016; Liu et al., 2019; Yan et al., 2019; Karvelis et al., 2020). Intriguingly, we find that nucleotides flanking the RNA-5′ side of protein-free R-loops remain stably paired and stacked in solution. This fundamental asymmetry in nucleic acid structure offers a functional explanation for the puzzling convergence of type V CRISPR-Cas systems on the 5′-repeat-spacer-3′ crRNA orientation.

## Results and discussion

### Cas12a binding to DNA distorts the target-strand cleavage site

**Figure 2.**
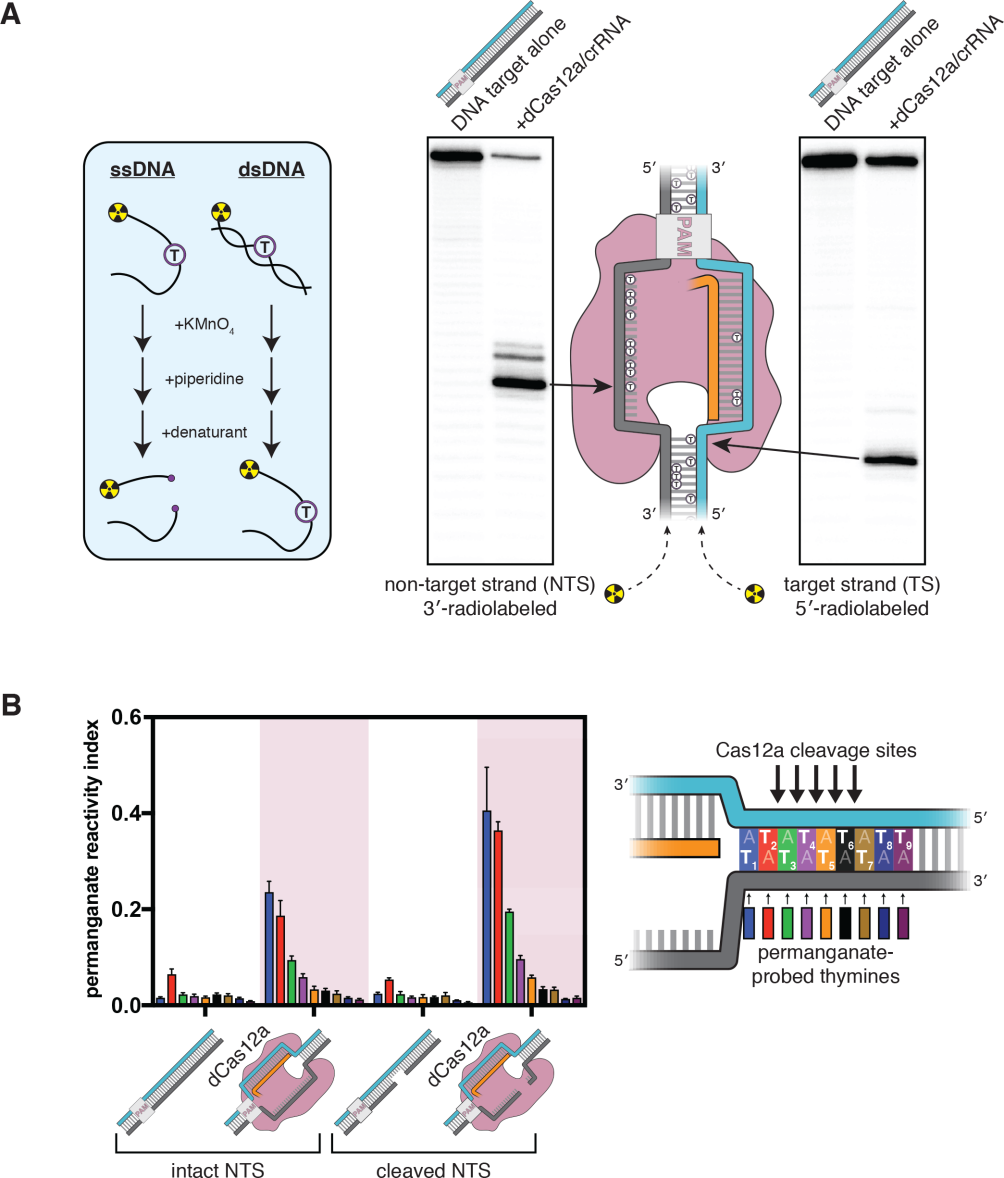
The target-strand cleavage site becomes distorted upon R-loop formation. (**A**) Denaturing PAGE phosphorimages of piperidine-treated permanganate oxidation products, demonstrating the assay’s ability to detect non-B-form DNA conformations within and adjacent to a dCas12a-generated R-loop. Permanganate reactions were quenched after 10 s at 30°C. Each thymine in the DNA substrate is shown as a circled T. (**B**) Permanganate reactivity of a PAM-distal R-loop flank whose sequence was changed (as compared to the native protospacer sequence that was probed in **A**) to contain more thymines, with an intact or cleaved non-target strand (‘cleaved NTS’ indicates that there is a 5-nt gap in the NTS—see Appendix 2). Permanganate reactions were quenched after 2 min at 30°C. A raw phosphorimage is shown in Figure 2—figure supplement 3. The permanganate reactivity index (PRI) is an estimate of the rate of oxidation at each thymine, normalized such that PRI = 1 for a fully single-stranded thymine (see Materials and methods). Columns and associated error bars indicate the mean and standard deviation of three replicates. The phosphodiester bonds normally cleaved by WT Cas12a are indicated with arrows on the substrate schematic for reference, but note that the complexes being probed with permanganate were formed with dCas12a. Figure 2—source data 1.Numerical data plotted in Figure 2 and accompanying figure supplements.

We suspected that dsDNA substrates of Cas12a would need to access a bent conformation to undergo target-strand cleavage. To chemically probe the structure of a Cas12a substrate in solution, we performed DNA permanganate footprinting on interference complexes containing a RuvC-inactivated mutant of a Cas12a ortholog from *Acidaminococcus species* (AsCas12a), hereafter called dCas12a. In this assay, permanganate selectively oxidizes thymines in non-B-form (e.g., locally melted or otherwise distorted) DNA structures, and oxidized positions are subsequently identified through piperidine-catalyzed strand cleavage (which occurs specifically at thymidine glycols) and denaturing polyacrylamide gel electrophoresis (PAGE) (Figure 2A; Bui et al., 2003). To enable sensitive detection of DNA fragments, we radiolabeled either the 5′ or 3′ end of each DNA strand (3′-end radiolabeling of DNA, which is not a common procedure, was achieved using a protocol developed for the present work, Figure 2—figure supplement 1). Consistent with previous applications of the permanganate assay to CRISPR-Cas-generated R-loops (Xiao et al., 2017), thymines within the portion of the non-target strand displaced by the crRNA were heavily oxidized, reflecting the single-strandedness of this DNA tract (Figure 2A).

**Figure 2—figure supplement 1.**
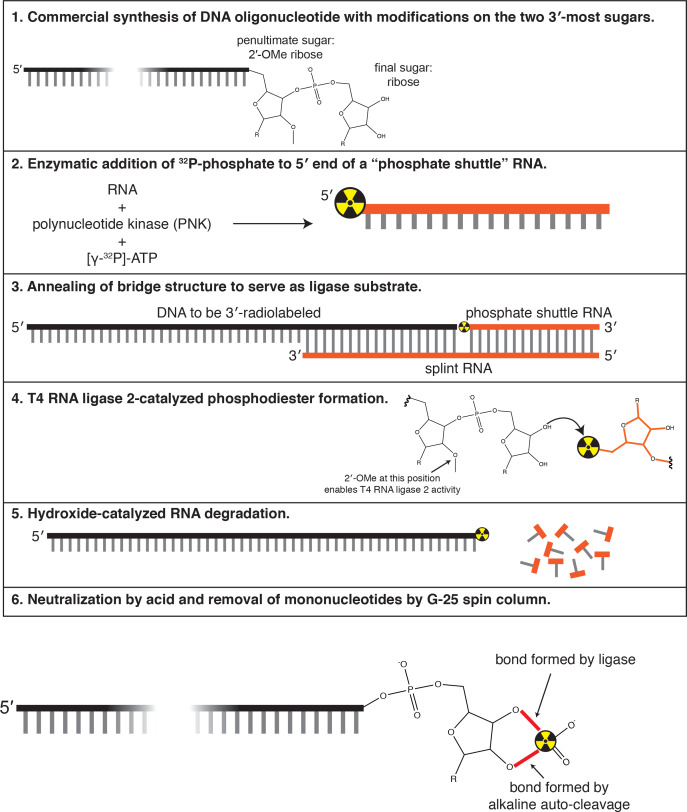
Method used to 3′-end radiolabel DNA oligonucleotides. See Materials and methods for details.

**Figure 2—figure supplement 2.**
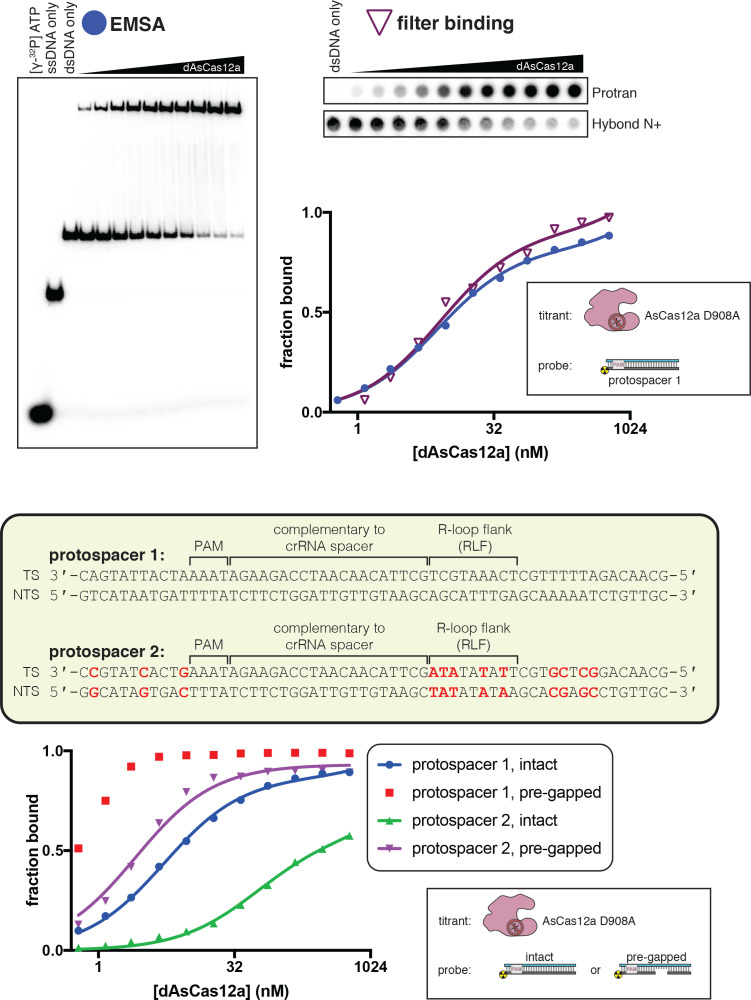
A gap in the non-target strand increases the affinity of dCas12a for its DNA target. *Top panel*: The affinity of dAsCas12a/crRNA for a cognate DNA target was assessed by an electrophoretic mobility shift assay (EMSA) and a filter-binding (FB) assay. dAsCas12a was titrated in a solution with fixed [crRNA] (750 nM) and [DNA probe] (100 pM), followed by separation of protein-bound DNA from free DNA. The EMSA indicated that the oligonucleotide annealing protocol yields 100% duplex DNA probe and that the binding conditions yield one major protein-bound species. ‘Fraction bound’ is defined as (background-subtracted volume of upper band)/(total background-subtracted lane volume) for the EMSA and (background-subtracted volume of Protran spot)/(total background-subtracted volume of Protran spot + Hybond N+ spot) for the filter-binding assay. The value of ‘fraction bound’ was 0 at [dAsCas12a]=0 for both assays (not shown in plot due to the logarithmic x-axis). When appropriate, data were fit to the sum of a hyperbola and a line (y = B_max_*x/(K_D_+x)+NS*x), where NS describes a non-specific binding mode. It is common to see B_max_ values below 1 in EMSAs and filter-binding assays, in which the process of physical separation can disrupt bound species. K_D_ for the EMSA was 8.2 nM (n = 1). K_D_ for the filter-binding assay was 8.1 nM ±0.8 (SD) (n = 3). *Bottom panel*: Using the filter binding assay, we assessed the affinity of dAsCas12a/crRNA for various cognate DNA targets. Protospacer 2 (used in Figure 2B) is the version of protospacer 1 (used in Figure 2A) modified for permanganate probing of the R-loop flank. Differences between protospacer 1 and protospacer 2 are highlighted in red (A/T base pairs substituted into the R-loop flank, G/C base pairs substituted elsewhere to maintain stable association between the two DNA strands). ‘Intact’ protospacers are as shown in the sequence schematic. ‘Pre-gapped’ protospacers are missing nt 14–18 of the NTS (as measured from the PAM, see Appendix 2). The value of ‘fraction bound’ was 0 at [dAsCas12a]=0 for all substrates (not shown due to the logarithmic x-axis). Data were analyzed as described for the top panel. K_D_ for protospacer 1 (intact) was 5 nM ±1 (SD) (n = 3). K_D_ for protospacer 2 (intact) was 54 nM ±12 (SD) (n = 3). K_D_ for protospacer 2 (pre-gapped) was 2.8 nM ±0.5 (SD) (n = 3). Data from the protospacer 1 (pre-gapped) experiment indicated that the K_D_ was near or below [DNA probe], preventing accurate K_D_ determination by hyperbolic fitting. The reason for the low observed affinity of dAsCas12a for protospacer 2 (intact) is unknown.

**Figure 2—figure supplement 3.**
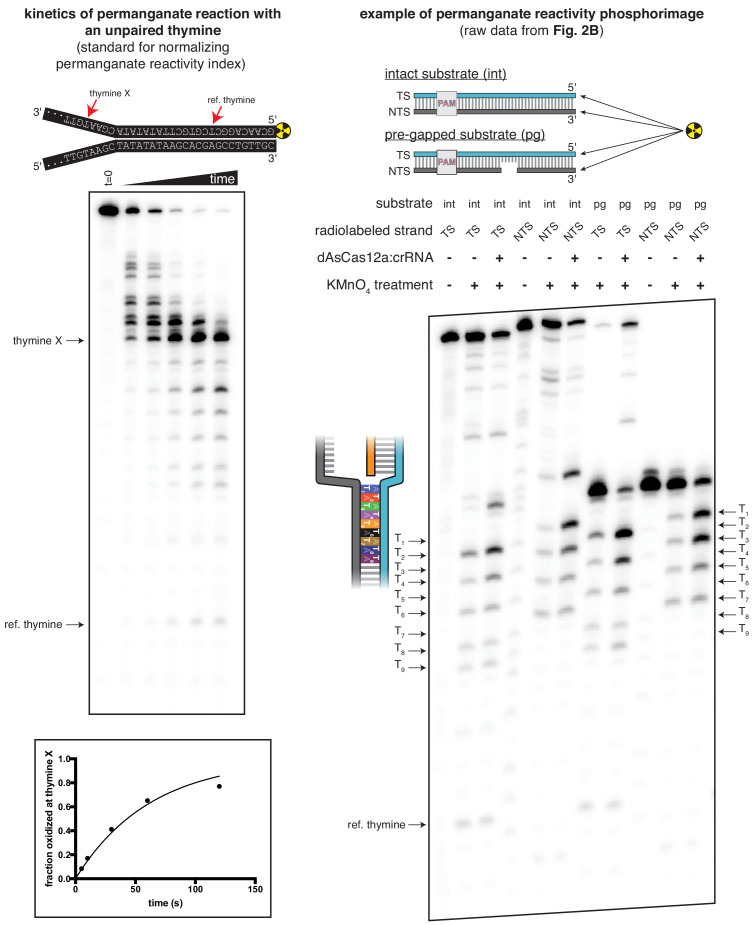
Translating raw phosphorimages into quantitative permanganate reactivity metrics. *Left panel*: Kinetics of permanganate reaction with an unpaired thymine. The depicted substrate was subject to the standard permanganate reaction protocol with quenching at 0, 5, 10, 30, 60, and 120 seconds. Black arrows indicate chemical cleavage fragments that resulted from oxidation of the annotated thymine. ‘Fraction oxidized at thymine X,’ plotted in the graph at the bottom, was determined as described in Materials and methods and is equivalent to the variable $p_{i}$. The phosphorimage and graph shown are from a single representative replicate (n = 3). Data were fit to an exponential decay (y = (y_0_-plateau)*exp(-k*x)+plateau), with y_0_ constrained to 0 and the plateau value constrained to 1. The value of k was determined to be 0.998 min^−1^ ±0.027 (SD) (n = 3), which, when corrected to the reference thymine, yielded the value of $k_{ss,corr}$=0.79 min^−1^ that was used for normalization of the permanganate reactivity index in all other permanganate experiments. *Right panel*: Raw phosphorimage of quantified data presented in Figure 2B. Black arrows indicate chemical cleavage fragments that resulted from oxidation of the annotated thymine. The method to determine the 'permanganate reactivity index' and 'fraction oxidized' metrics from a raw phosphorimage is described in Materials and methods.

Interestingly, we also observed significant oxidation at a thymine near the target-strand cleavage site (Figure 2A). To probe the region around the target-strand cleavage site more thoroughly, we adjusted the sequence of the DNA substrate to contain a stretch of A/T base pairs in the tract immediately adjacent to the R-loop, which we denote the R-loop flank (Figure 2B, Figure 2—figure supplement 2). We assessed the permanganate reactivity of the R-loop flank in three states of the Cas12a cleavage pathway: prior to Cas12a binding (apo DNA), after R-loop formation, and after the first set of cleavage events, which yield a 5-nt gap in the non-target strand (see Appendix 2 for details of the NTS gap). At each step, we observed an increase in permanganate reactivity on both strands of the DNA that persisted seven base pairs past the end of the crRNA, suggesting that Cas12a binding promotes distortion of DNA in the PAM-distal flank of the R-loop (Figure 2B, Figure 2—figure supplement 3, see Materials and methods).

In general, enhanced permanganate reactivity could reflect any of a variety of departures from B-form DNA duplex geometry, ranging from slight helical distortion to complete strand separation (Bui et al., 2003). As a result, the precise conformational ensemble of the R-loop flank cannot be absolutely determined from permanganate reactivity measurements. However, reactivity in the probed region was on the same order of magnitude as that of a fully single-stranded control, suggesting that the detected distortion involves substantial nucleobase unpairing and unstacking (Figure 2B, Figure 2—figure supplement 3, see Materials and methods). Additionally, permanganate reactivity decreased with distance from the R-loop edge (Figure 2B), consistent with NTS:TS fraying events that initiate from the R-loop edge (see Materials and methods). The increase in bulk permanganate reactivity in response to NTS cleavage may be due, at least in part, to increased binding occupancy of dCas12a/crRNA on the DNA substrate, as the NTS gap creates a high-energy interruption in the DNA rewinding pathway that boosts the stability of the ribonucleoprotein:DNA interaction (Figure 2—figure supplement 2; Knott et al., 2019). Thus, while the R-loop flank has the potential for complete base pairing, this DNA tract populates highly distorted, and probably unpaired, conformations when bound to Cas12a in solution.

### Distortion of the R-loop flank facilitates target-strand cleavage

DNA discontinuities as small as a nick or a single unpaired nucleotide are known to produce a ‘swivel-like’ point of flexibility within a duplex structure (Mills et al., 1994; Le Cam et al., 1994). We wondered whether the DNA distortion detected in the Cas12a R-loop flank may permit the global substrate bending and repositioning that is likely required for target-strand cleavage. A prediction of this model is that a change in the location of duplex instability should also change which phosphodiester bonds in the target strand are cleaved by the RuvC DNase. To test this prediction, we investigated the effect of R-loop size on the target-strand cut-site distribution. We reasoned that if DNA strand separation occurs at the R-loop edge, the location of the R-loop edge must define which nucleotides become unpaired and, consequently, which bonds can be accessed by the RuvC active site.

**Figure 3.**
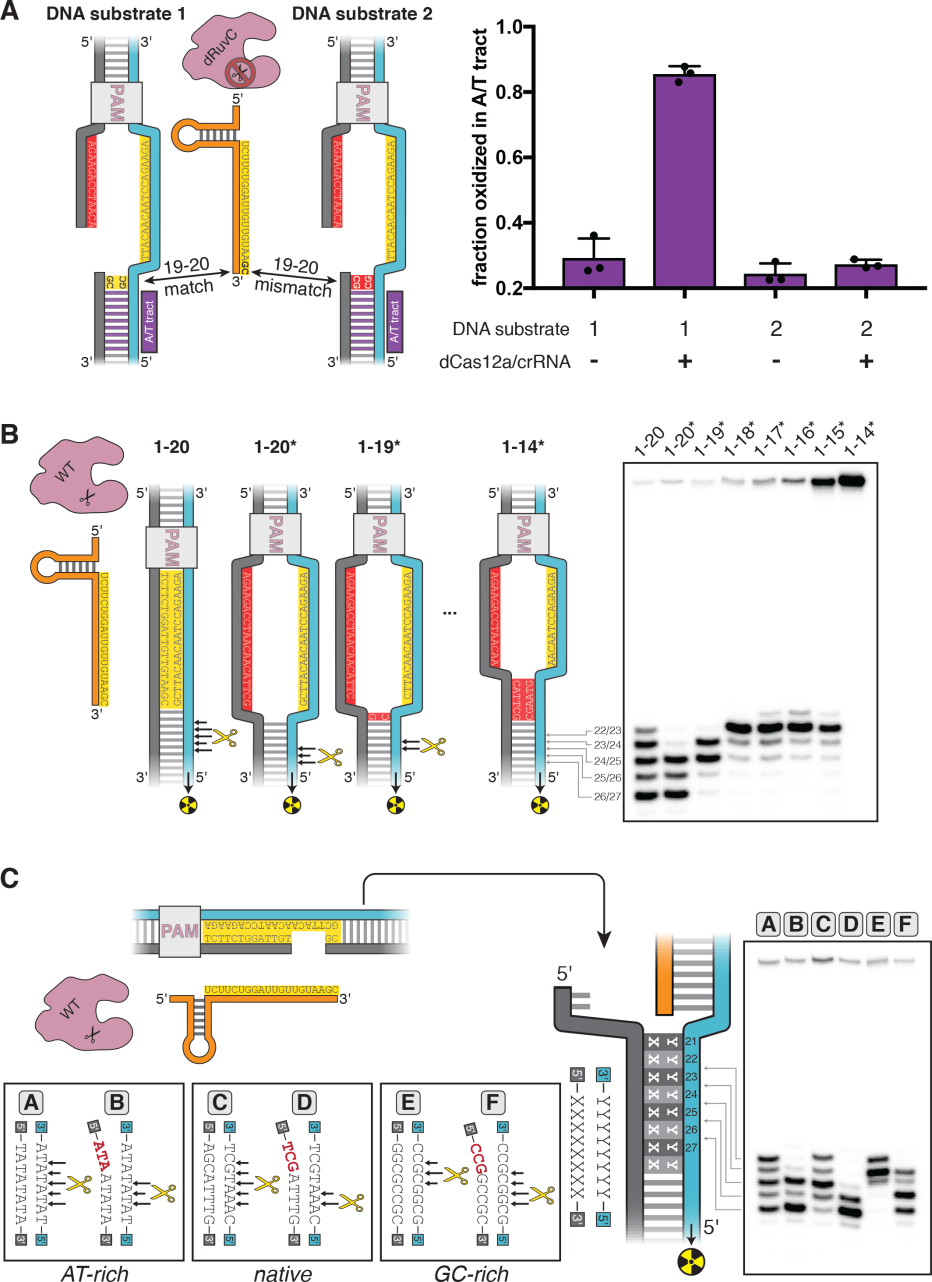
DNA distortion in the R-loop flank facilitates target-strand cleavage. (**A**) Permanganate reactivity of A/T tract in a 20-nt R-loop and an 18-nt R-loop. Permanganate experiments were conducted as in Figure 2B (2 minutes, 30°C). Purple rectangles alongside DNA schematics indicate the location of the tract of DNA whose permanganate reactivity is being quantified. The y-axis denotes the fraction of DNA molecules estimated to have been oxidized on at least one thymine within the A/T tract (see Materials and methods). Columns and associated error bars indicate the mean and standard deviation of three replicates. (**B**) Target-strand cut-site distribution with a shrinking R-loop, as resolved by denaturing PAGE and phosphorimaging (n = 3). 100 nM AsCas12a and 120 nM crRNA were incubated with 1 nM of DNA target at 37°C for 1 hr, prior to quenching and resolution by denaturing PAGE (kinetics shown in Figure 3—figure supplement 4). Each lane corresponds to a different DNA target, bearing varying numbers of PAM-distal mismatches with respect to the crRNA. Indicated above each lane is the number of base pairs of complementarity between the target strand and the crRNA spacer, starting with the base immediately adjacent to the PAM. For the lane lacking an asterisk, the DNA target was fully duplex. For the lanes that bear asterisks, the DNA target contained a bubble across the region of crRNA:TS complementarity, which stabilized the interaction of the DNA with the Cas12a/crRNA complex. Numbers to the left of the phosphorimage indicate the position (distance from the PAM, as numbered in **C**) of the dinucleotide whose phosphodiester was cleaved to yield the labeled band. Black arrows are drawn on the substrate diagrams to indicate cleaved phosphodiesters (as determined from the phosphorimage), and relative arrow lengths are roughly reflective of relative band intensities. (**C**) Target-strand cut-site distribution with various sequences in the R-loop flank (all with a 20-nt R-loop), as resolved by denaturing PAGE and phosphorimaging (n = 3). 100 nM AsCas12a and 120 nM crRNA were incubated with 1 nM of DNA target at 25°C for 10 min, prior to quenching and resolution by denaturing PAGE (kinetics shown in Figure 3—figure supplement 7). All DNA targets were 5'-radiolabeled on the target strand. The non-target strand contained a gap from positions 14–18 (see Appendix 2) but was complementary to the target strand at positions 1–13 and 19–20. In each lane, the DNA target was varied to contain different sequences in the R-loop flank, which either formed a perfect duplex (substrates A, C, and E) or contained a 3-bp NTS:TS mismatch (substrates B, D, and F). Black arrows are drawn on the substrate diagrams as in **B**. Figure 3—source data 1.Numerical data plotted in Figure 3 and accompanying figure supplements.

We first assessed our ability to shift the site of duplex instability by measuring permanganate reactivity in a truncated R-loop. To create a truncated R-loop, we mutated the DNA base pairs at positions 19 and 20, allowing crRNA strand invasion to progress only to the 18^th^ nucleotide of the target sequence (position numbers indicate distance from the PAM). Additionally, we used a pre-cleaved non-target strand to lock the substrate in the chemical state that exists immediately before target-strand cleavage (Appendix 2). Finally, we prevented NTS:TS base pairing in the PAM-proximal part of the DNA target sequence by mutating the non-target strand at positions 1–12 (Figure 3A). This modification eliminated the branch-migration-catalyzed DNA dissociation pathway, allowing for the formation of uniformly stable complexes irrespective of PAM-distal crRNA:TS mismatches that would otherwise modulate affinity (Strohkendl et al., 2018; Figure 3—figure supplement 1). As a result, variations in the bulk permanganate reactivity of these constructs reflect variations in DNA structure rather than differential Cas12a/crRNA binding occupancy.

**Figure 3—figure supplement 1.**
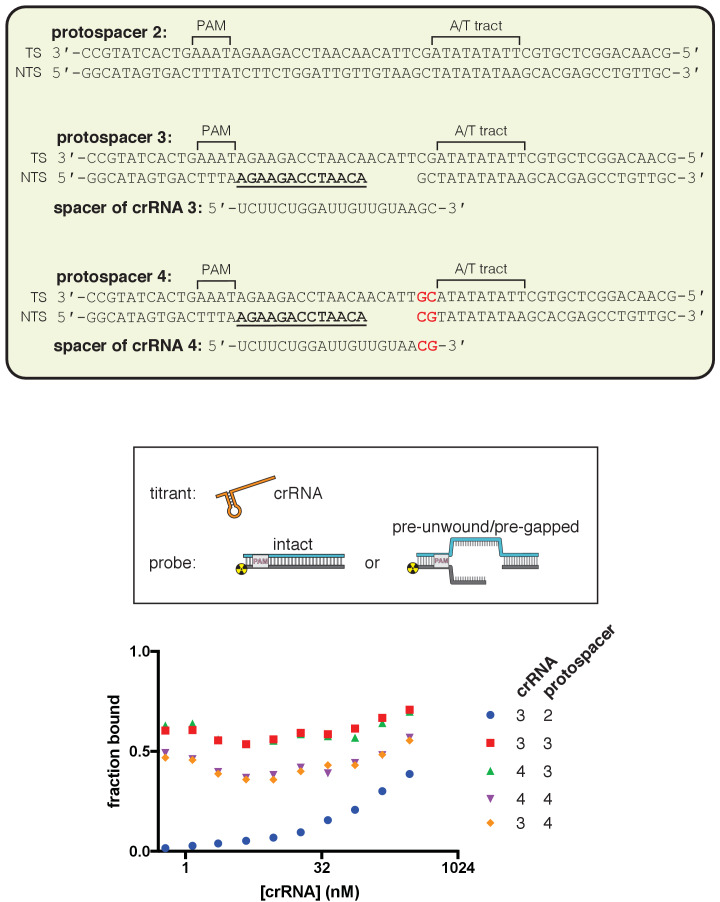
dCas12a ribonucleoprotein binds tightly to pre-gapped/pre-unwound targets despite PAM-distal mismatches. The affinity of dAsCas12a/crRNA for various cognate DNA targets was assessed by a filter-binding assay. ‘Pre-gapped’ indicates the presence of a 5-nt gap in the non-target strand (see Appendix 2). ‘Pre-unwound’ indicates the presence of a stretch of NTS:TS mismatches in the DNA substrate. In Figure 3A, protospacer 3 is annotated as ‘DNA substrate 1;’ protospacer 4 is annotated as ‘DNA substrate 2;’ and crRNA 3 is the depicted crRNA. For each combination of crRNA/DNA target, crRNA was titrated in a solution with fixed [dAsCas12a] (400 nM), [DNA probe] (100 pM), and [non-specific DNA competitor] (500 nM). The identities of the titrant/fixed component were inverted in this experiment (as compared to all other binding experiments) because crRNA can form a stable complex with pre-unwound DNA targets in the absence of protein. Keeping [dAsCas12a] at 400 nM favored the formation of (dAsCas12a/crRNA):DNA complexes over crRNA:DNA complexes (which would be indistinguishable from free DNA in the filter binding assay). In the presence of high [apo protein], 500 nM non-specific DNA competitor (a duplex with a short ssDNA overhang) was also included to disfavor non-specific interactions between radiolabeled DNA and apo protein. The value of ‘fraction bound’ was 0 at [crRNA]=0 for all substrates (not shown due to the logarithmic x-axis). For all pre-unwound DNA targets, the fraction bound was essentially concentration-independent across all nonzero concentrations tested, suggesting that the lowest concentration tested had already saturated the specific binding interaction being probed. The high stability is in line with thermodynamic expectations for an interaction involving hybridization of two complementary 18-nt or 20-nt oligonucleotides (T_m_ > 40°C) (Kibbe, 2007). The fact that the saturated bound fraction is less than 1 could be due to (1) a common feature of filter-binding assays in which the process of physical separation disrupts bound species or (2) a stable population of protein-free crRNA:DNA complexes. In any case, the important conclusion to be drawn from these data is that each protospacer exhibits the same fraction bound regardless of the presence of mismatches at positions 19 and 20 in the crRNA. Thus, the crRNA-dependent effects seen in Figure 3A and Figure 3—figure supplement 2 must emerge from fundamental differences in conformational dynamics and not from differences in binding occupancy of Cas12a/crRNA on the DNA probe.

**Figure 3—figure supplement 2.**
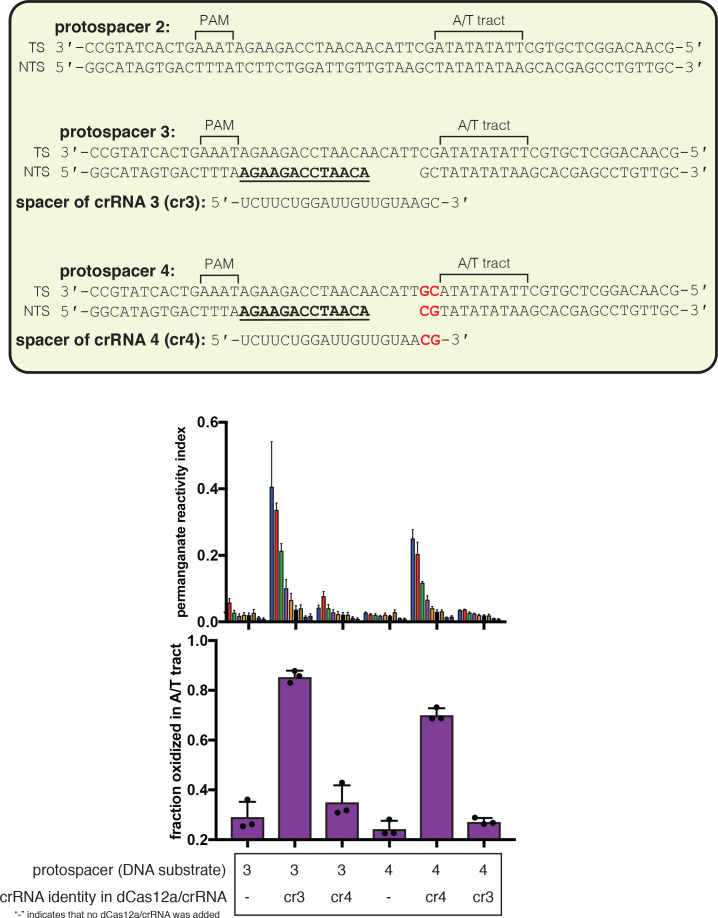
Effect of R-loop truncation on permanganate reactivity of the A/T tract. Permanganate reactivity of the A/T tract in a 20-nt R-loop and an 18-nt R-loop. In Figure 3A, protospacer 3 is annotated as ‘DNA substrate 1;’ protospacer 4 is annotated as ‘DNA substrate 2;’ and crRNA 3 is the depicted crRNA. Permanganate experiments were conducted as in Figure 2B (2 minutes, 30°C). See Materials and methods for description of the parameters plotted on the y-axis. Columns and associated error bars indicate the mean and standard deviation of three replicates. Columns 1, 2, 4, and 6 are equivalent to the data shown in Figure 3A. Columns 3 and 5 use a crRNA with compensatory mutations at positions 19–20, showing that the effect is dependent upon base pairing topology and not a particular sequence.

**Figure 3—figure supplement 3.**
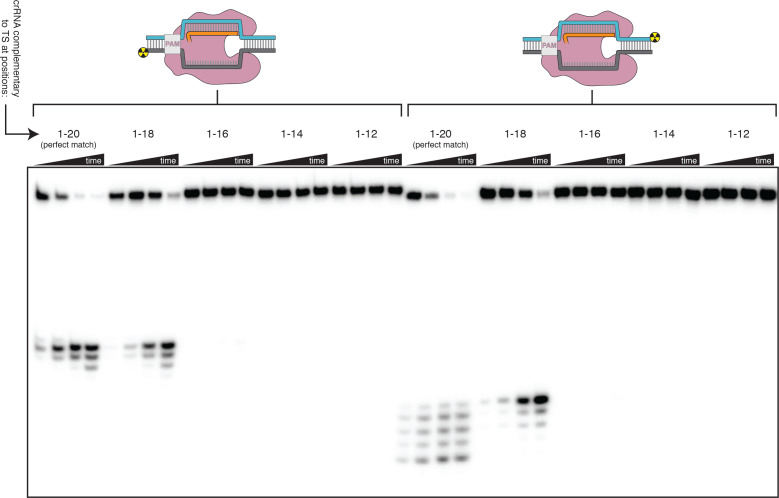
Effect of PAM-distal mismatches on non-target-strand and target-strand cleavage kinetics and position with fully duplex DNA targets. 100 nM AsCas12a and 120 nM crRNA were incubated with 1 nM of DNA target at 37°C for 20 s, 1 min, 5 min, and 30 min, prior to quenching and resolution by denaturing PAGE. Each group of four lanes corresponds to a different DNA target, with varying numbers of PAM-distal mismatches with respect to the crRNA. Indicated above each group of four lanes is the number of base pairs of complementarity between the TS and the crRNA spacer, starting with the base immediately adjacent to the PAM. All DNA targets in this gel were fully duplex (not pre-unwound/bubbled), resulting in enhanced discrimination against PAM-distal mismatches as compared to the bubbled DNA targets in Figure 3B and Figure 3—figure supplement 4.

**Figure 3—figure supplement 4.**
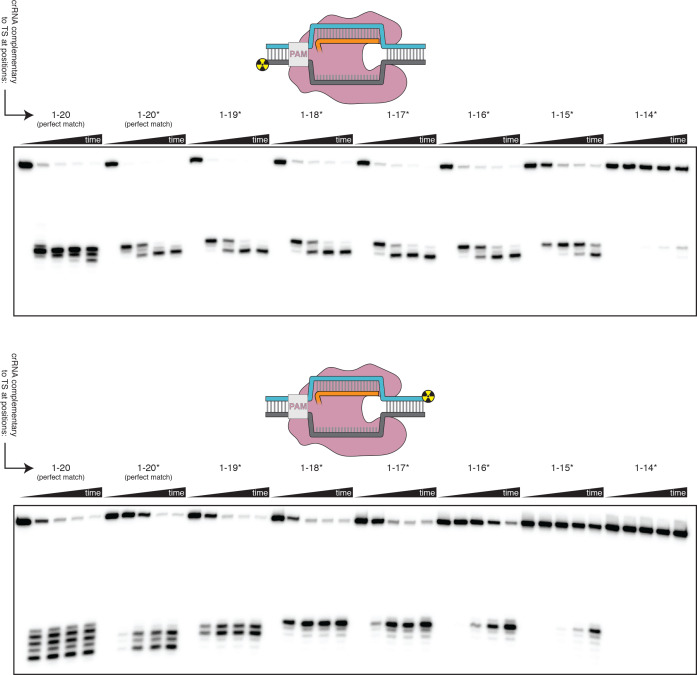
Effect of PAM-distal mismatches on non-target-strand and target-strand cleavage kinetics and position with bubbled DNA targets. 100 nM AsCas12a and 120 nM crRNA were incubated with 1 nM of DNA target at 37°C for 0 s, 15 s, 2 min, 10 min, and 1 hr, prior to quenching and resolution by denaturing PAGE. Each time series corresponds to a different DNA target, bearing varying numbers of PAM-distal mismatches with respect to the crRNA. Indicated above each time series is the number of base pairs of complementarity between the TS and the crRNA spacer, starting with the base immediately adjacent to the PAM. For the time series lacking an asterisk, the DNA target was fully duplex (as in Figure 3—figure supplement 3). For the time series that bear asterisks, the DNA target contained a bubble across the region of crRNA:TS complementarity (as illustrated in Figure 3B), which stabilized the R-loop. In the top panel, the NTS was 5'-radiolabeled. In the bottom panel, the TS was 5'-radiolabeled.

**Figure 3—figure supplement 5.**
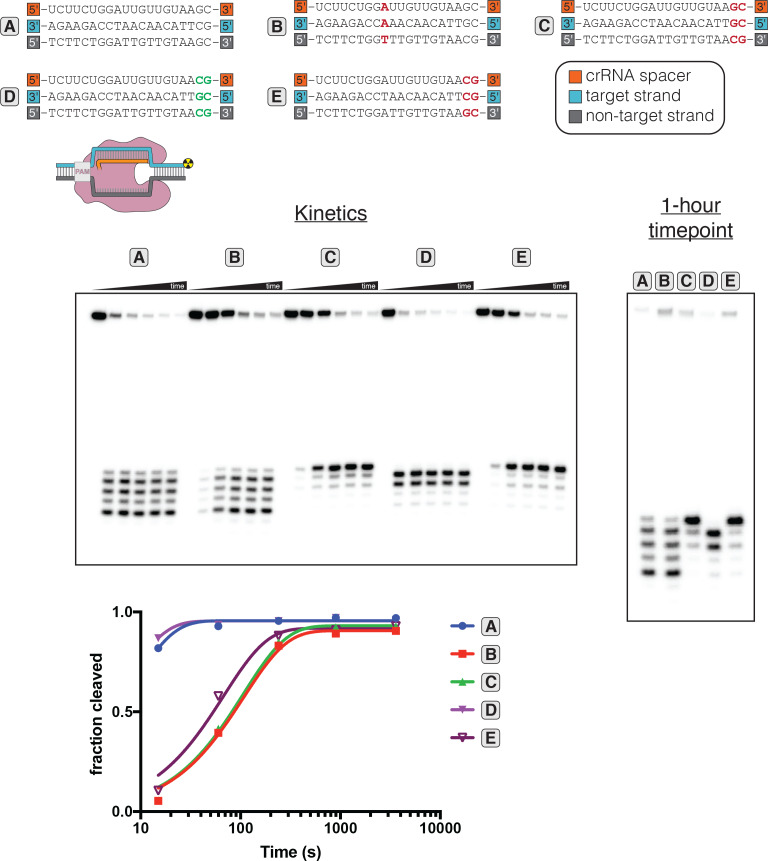
Determinants of altered target-strand cleavage kinetics and position. 100 nM AsCas12a and 120 nM crRNA were incubated with 1 nM duplex DNA target radiolabeled on the 5' end of the target strand at 37°C for 0 s, 15 s, 1 min, 4 min, 15 min, or 1 hr, prior to quenching and resolution by denaturing PAGE. The 20-nt target sequence immediately adjacent to the PAM is shown below the crRNA spacer sequence used in each experiment. Red letters indicate TS:crRNA mismatches. Green letters indicate compensatory changes in the crRNA to restore a 20-nt match. The final timepoint of each reaction is reproduced in the gel on the right, for side-by-side comparison of the cleavage site distributions. ‘Fraction cleaved’ is defined as (sum of the volume of all bands below the uncleaved band)/(total volume in lane). Data were fit to an exponential decay (y = (y_0_-plateau)*exp(-k*x)+plateau), with y_0_ constrained to 0 and the plateau value constrained to ≤1. A representative replicate is shown. The value of k_obs_ for each time course is as follows: A (0.12 s^−1^), B (0.0093 s^−1^), C (0.0094 s^−1^), D (0.16 s^−1^), E (0.015 s^−1^). The precise value of k_obs_ for A and D should be interpreted with caution due to poor sampling of informative timepoints.

**Figure 3—figure supplement 6.**
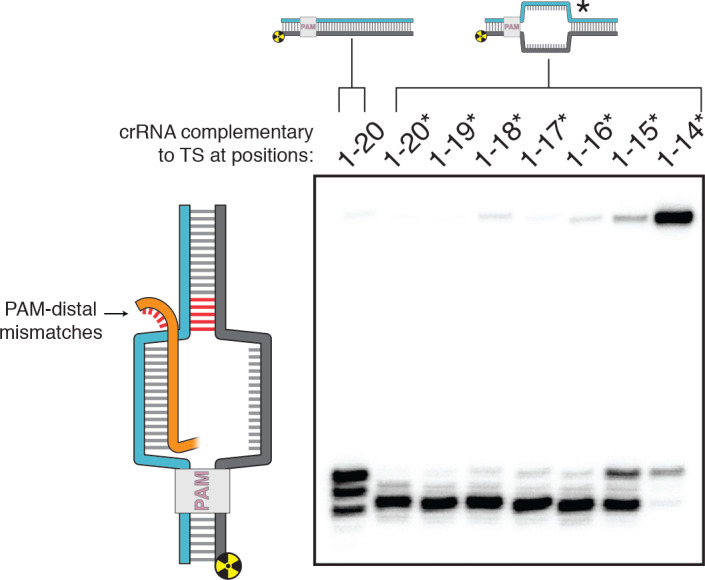
Non-target-strand cut-site distribution with a shrinking R-loop. Final timepoint (1 hr) of each time series in the non-target-strand gel shown in the top panel of Figure 3—figure supplement 4, shown side-by-side for visual comparison—analogous to the final timepoints for the target strand shown in Figure 3B.

**Figure 3—figure supplement 7.**
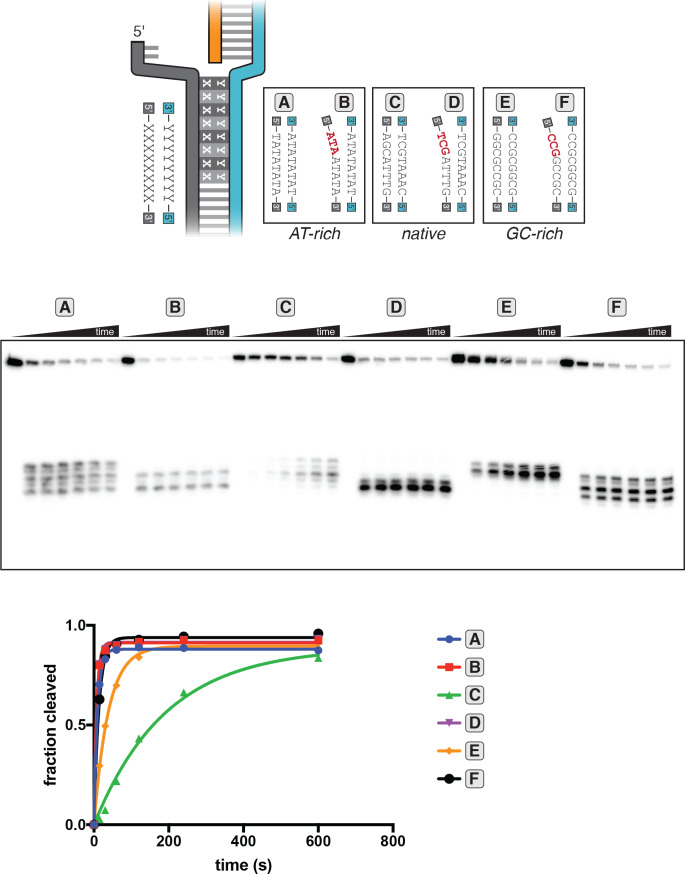
Kinetics of target-strand cleavage in DNA targets with various sequences in the R-loop flank. Experiment performed as described in legend to Figure 3C. 100 nM AsCas12a and 120 nM crRNA were incubated with 1 nM of DNA target at 25°C for 0 s, 15 s, 30 s, 1 min, 2 min, 4 min, or 10 min, prior to quenching and resolution by denaturing PAGE. All DNA targets were 5'-radiolabeled on the TS. The NTS was pre-gapped from positions 14–18 but complementary to the TS at positions 1–13 and 19–20. In each lane, the DNA target was varied to contain different sequences in the R-loop flank, which either formed a perfect duplex (substrates A, C, and E) or contained a 3-bp NTS:TS mismatch (substrates B, D, and F). ‘Fraction cleaved’ is defined as (sum of the volume of all bands below the uncleaved band)/(total volume in lane). Data were fit to an exponential decay (y=(y_0_-plateau)*exp(-k*x)+plateau), with y_0_ constrained to 0. A representative replicate (n = 3) is shown. The value of k_obs_ ± SD for each time course is as follows: A [0.092 ± 0.012 s^−1^], B [0.145 ± 0.007 s^−1^], C [0.0059 ± 0.0006 s^−1^], D [0.137 ± 0.002 s^−1^], E [0.024 ± 0.002 s^−1^], F [0.061 ± 0.013 s^−1^]. The rate constants for B and D should be interpreted with caution due to poor sampling of informative timepoints.

By implementing the permanganate assay on these complexes, we found that the A/T tract was highly reactive in the full R-loop but had limited reactivity in the truncated R-loop, suggesting that the distorted region had migrated with the edge of the R-loop (Figure 3A). We also observed this effect for a second set of crRNA/DNA sequences with equivalent base-pairing topology, demonstrating that this result is not unique to the originally tested sequence (Figure 3—figure supplement 2). Positions 19 and 20 of the DNA substrates were G/C base pairs, so DNA conformation at these nucleotides could not be assessed by permanganate reactivity. Nonetheless, these results show that nucleotide unpairing near the target-strand cleavage site depends not only on Cas12a binding and stable R-loop formation, but also on the extent of crRNA strand invasion (i.e., the size of the R-loop). Thus, by altering R-loop size, we can manipulate which nucleotides become unpaired upon Cas12a binding.

To test the hypothesis that distortion in the R-loop flank is linked to RuvC-mediated target-strand cleavage, we assembled wild type (WT) Cas12a with R-loops of various sizes and determined the distribution of target-strand cut sites. For these experiments, we used DNA substrates with an intact non-target strand that was mismatched with respect to the target strand throughout the region of crRNA complementarity (Figure 3B; DNA constructs containing such a mismatched tract are indicated with an asterisk—for example, 1–18* indicates a DNA construct whose target-strand sequence matches the crRNA sequence at positions 1–18 and whose non-target-strand is mismatched with respect to the target strand at the same positions). Preventing NTS:TS base pairing in this region allowed observation of DNA cleavage in enzyme-substrate complexes that would otherwise be too unstable to yield detectable rates of catalysis (Strohkendl et al., 2018; Figure 3—figure supplement 3).

As the R-loop edge was shifted toward the PAM, the target-strand cut-site distribution shifted toward the new R-loop edge (Figure 3B, Figure 3—figure supplement 4). When compensatory mutations were made in the crRNA to restore the original R-loop size, the target-strand cut sites moved back toward their original distribution (Figure 3—figure supplement 5). The observed shifts in the cut sites were not due to general destabilization of the R-loop, as a single crRNA:TS mismatch at an internal position of the target sequence (position 9) slowed cleavage without affecting cut-site distribution (Figure 3—figure supplement 5). The cut-site distribution shared by the 1–17*, 1–16*, and 1–15* substrates, along with the lack of cleavage of the 1–14* substrate, may reveal a geometric limit on bent DNA conformations that still permit active site association (Figure 3B). Additionally, the broader target-strand cut-site distribution in the DNA target lacking NTS:TS mismatches (labeled ‘1–20’ in Figure 3B) could reflect bending events that initiated from partially rewound R-loop conformations. Notably, the non-target-strand cut-site distribution did not change markedly as the R-loop was truncated, suggesting that non-target-strand cleavage is unrelated to nucleotide unpairing in the R-loop flank (Figure 3—figure supplement 6). These results imply that the site of Cas12a-mediated target-strand cleavage is tied to, and perhaps dictated by, the location of weakened base pairing. Consistent with this idea, a previous study found a linkage between Cas12a cut-site distribution and nucleic acid conformation as measured by Förster resonance energy transfer (Zhang et al., 2019).

These principles predict that R-loop flank sequences with greater nucleobase stacking energy should limit the depth of fraying and, consequently, favor target-strand cleavage events that are closer to the PAM. In agreement with this prediction, of three DNA targets that differed only in the sequence of their R-loop flank—native protospacer, AT-rich, or GC-rich—the GC-rich substrate was cleaved most PAM-proximally (Figure 3C). Additionally, eliminating NTS:TS base pairing at positions 21–23 led to fast and PAM-distally shifted cleavage of the target strand in all cases (Figure 3C, Figure 3—figure supplement 7). Together, these results suggest that DNA distortion in the R-loop flank is an important enabler of Cas12a-catalyzed target-strand cleavage.

### Duplex instability is intrinsic to DNA in the RNA-3′ flank of R-loops

**Figure 4.**
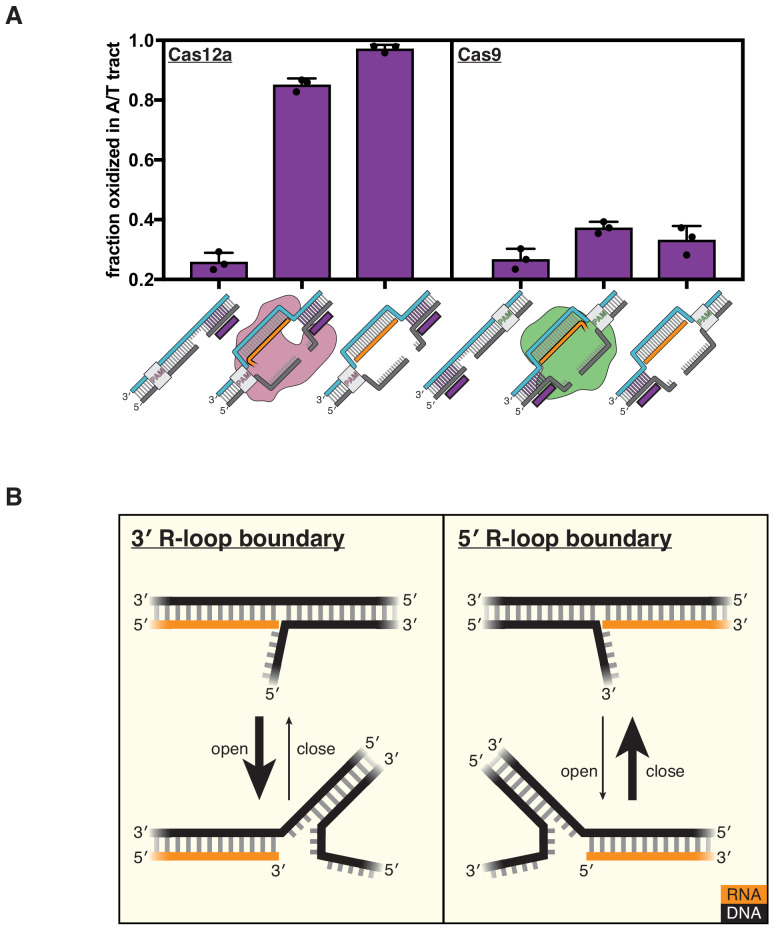
DNA distortion is protein-independent and unique to 3' R-loop flanks. (**A**) Permanganate reactivity of the A/T tract in a dCas12a R-loop, a dCas9 R-loop, and their protein-free mimics. The y-axis denotes the fraction of DNA molecules estimated to have been oxidized on at least one thymine within the A/T tract (see Materials and methods). Purple rectangles alongside DNA schematics indicate the location of the tract of DNA whose permanganate reactivity is being quantified. Columns and associated error bars indicate the mean and standard deviation of three replicates. (**B**) Model for the relative conformational dynamics of 3' and 5' R-loop boundaries, as suggested by permanganate reactivity experiments. The depth of fraying shown (three base pairs) was chosen arbitrarily for the schematic and should not be interpreted as a uniquely stable ‘open’ structure (see Materials and methods). Figure 4—source data 1.Numerical data plotted in Figure 4 and accompanying figure supplements.

Next, we wondered what role the Cas12a protein plays in distortion of the R-loop flank. To assess the contribution of the protein, we formed a protein-free mimic of the nucleic acid structure immediately prior to target-strand cleavage. This artificial R-loop contained a pre-cleaved non-target strand that was mismatched with respect to the target strand in the 20-nt stretch adjacent to the PAM, and the same stretch of the target strand was hybridized to a complementary 20-nt RNA oligonucleotide (Figure 4A). When we probed the permanganate reactivity of this protein-free R-loop, we found that the A/T tract was slightly more reactive than in the Cas12a-generated R-loop experiment (Figure 4A, Figure 4—figure supplement 1).

**Figure 4—figure supplement 1.**
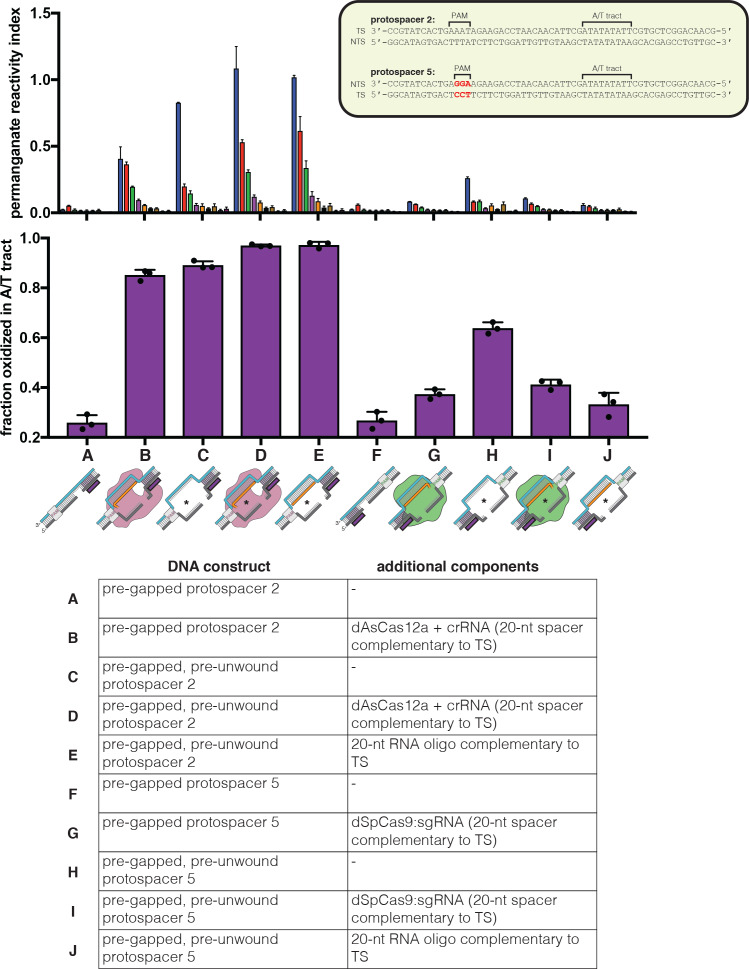
Permanganate reactivity of the A/T tract in R-loops formed by dCas12a or dCas9. Permanganate experiments were conducted as in Figure 2B (2 minutes, 30°C). ‘Pre-gapped’ indicates the presence of a 5-nt gap in the non-target strand (see Appendix 2) (the NTS gap in the dCas9 target is unrelated to the cut that would normally be formed by a nuclease-active Cas9—instead, it was designed to be analogous to the NTS gap formed by AsCas12a in an R-loop of the opposite topology, at positions 14–18). ‘Pre-unwound’ indicates the presence of a stretch of NTS:TS mismatches in the DNA substrate (20-nt bubble throughout the region of RNA complementarity); asterisks highlight the constructs that contain these NTS:TS mismatches. The sequence of protospacer 5 is identical to that of protospacer 2 except for a change in the PAM, which is not expected to affect conformational dynamics at the A/T tract (besides in permitting dCas9 binding). See Materials and methods for description of the parameters plotted on the y-axis. Columns and associated error bars indicate the mean and standard deviation of three replicates. Experiments A, B, E, F, G, and J are equivalent to the data shown in Figure 4A.

**Figure 4—figure supplement 2.**
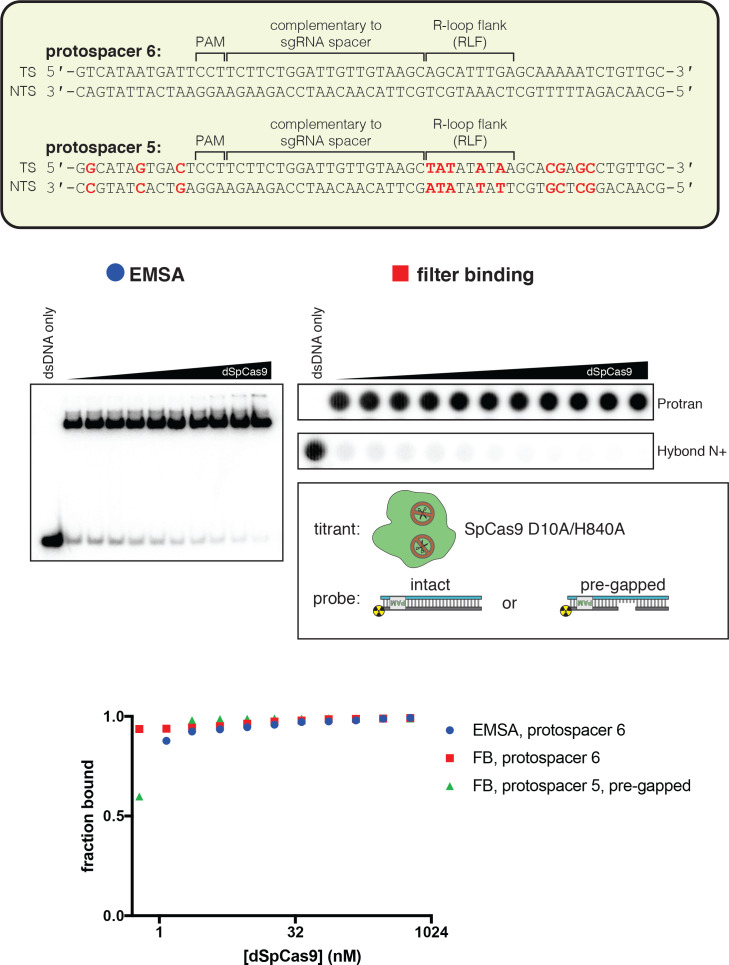
dCas9 binds tightly to pre-gapped DNA targets. The affinity of dSpCas9/sgRNA for a cognate DNA target was assessed by an electrophoretic mobility shift assay (EMSA) and a filter-binding (FB) assay. dSpCas9 was titrated in a solution with fixed [sgRNA] (750 nM) and [DNA probe] (100 pM), followed by separation of protein-bound DNA from free DNA. The EMSA indicated that the binding conditions yield one major protein-bound species. ‘Fraction bound’ is defined as (background-subtracted volume of upper band)/(total background-subtracted lane volume) for the EMSA and (background-subtracted volume of Protran spot)/(total background-subtracted volume of Protran spot + Hybond N+ spot) for the filter-binding assay. The value of ‘fraction bound’ was 0 at [dSpCas9]=0 for both substrates and both assays (not shown on plot due to the logarithmic x-axis). All data shown are from one representative replicate (n = 1 for EMSA, n = 3 for FB). Protospacer 6 is identical to protospacer 1 (used for AsCas12a), except the PAM has been substituted with a SpCas9 PAM. Protospacer 5 is a modified version of protospacer 6, with differences highlighted in red (equivalent to the protospacer-1-to-protospacer-2 modifications). The ‘intact’ protospacer is as shown in the sequence schematic. The ‘pre-gapped’ protospacer is missing nt 14–18 of the NTS (as measured from the PAM). The NTS gap is unrelated to the cut that would normally be formed by a nuclease-active Cas9—instead, it was designed to be analogous to the NTS gap formed by AsCas12a in an R-loop of the opposite topology, at positions 14–18. This DNA substrate binds tightly to dSpCas9. Thus, the failure of dSpCas9 to significantly distort the R-loop flank in Figure 4A is due to a fundamental difference in conformational dynamics and not to a failure to bind.

**Figure 4—figure supplement 3.**
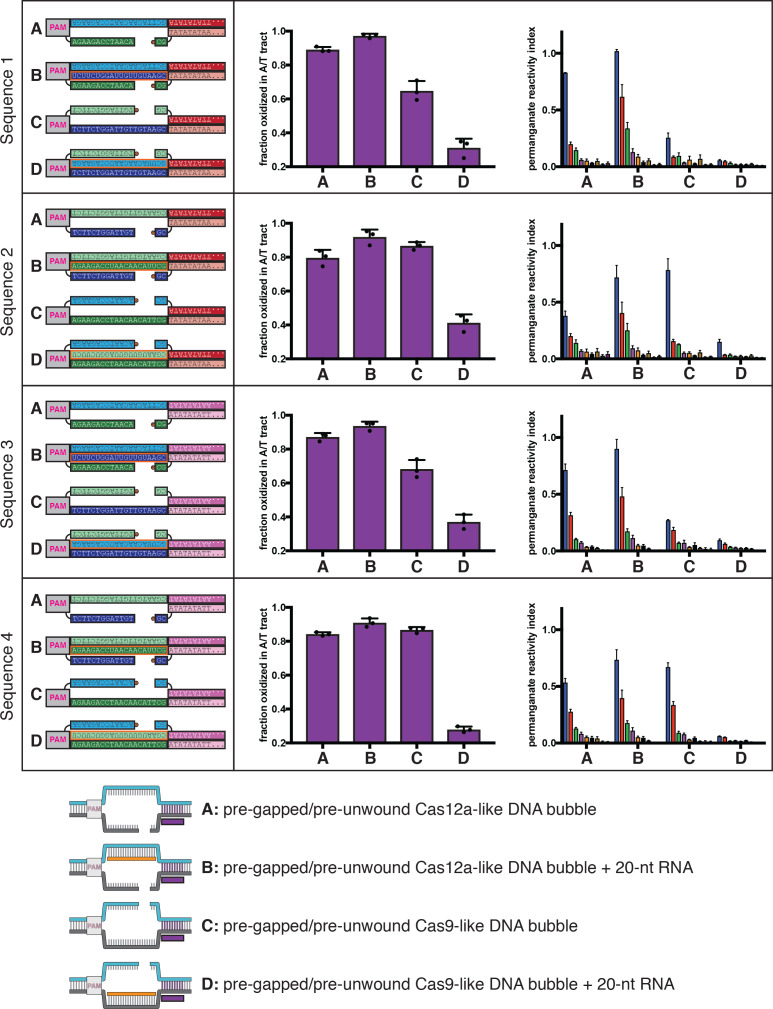
Permanganate reactivity of the A/T tract in protein-free R-loops of various sequences. Permanganate experiments were conducted as in Figure 2B (2 minutes, 30°C). See Materials and methods for description of the parameters plotted on the y-axis. Columns and associated error bars indicate the mean and standard deviation of three replicates. In all schematics, RNA molecules are outlined in orange, and DNA molecules are outlined in black. Circled ‘P’ indicates a 5′-phosphate. All sequences, when read right-side up, go from 5′ on the left to 3′ on the right. The terms ‘Cas12a-like’ and ‘Cas9-like’ are descriptors only of each substrate’s R-loop topology (the end of the RNA next to the boundary of interest is a 3′ end or a 5′ end, respectively)—both kinds of substrates contain a Cas12a PAM and a Cas12a-like NTS gap. These results imply that the asymmetry in R-loop-flank stability is a fundamental feature of R-loop structure and not a peculiarity of the original tested sequence.

**Figure 4—figure supplement 4.**
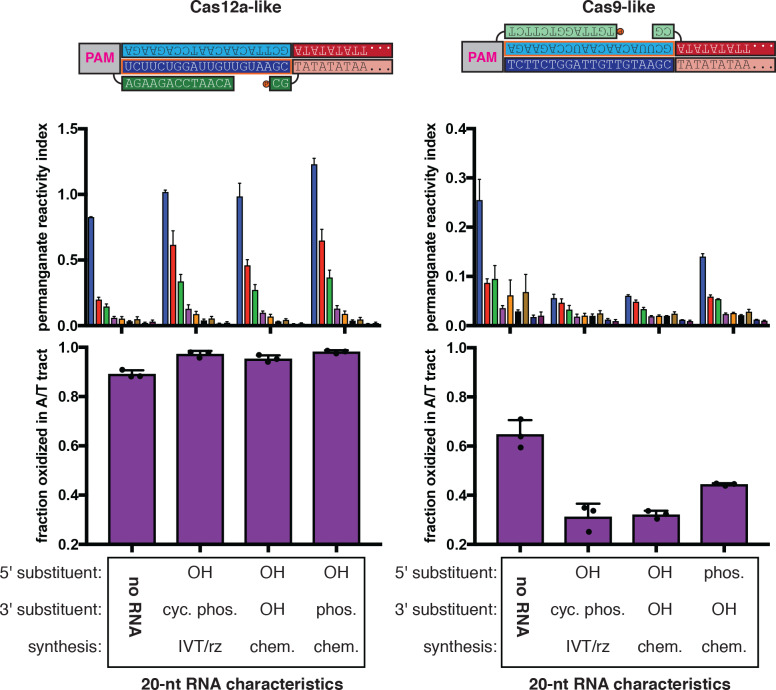
Effect of RNA end chemistry on permanganate reactivity of the A/T tract in protein-free R-loops. Permanganate experiments were conducted as in Figure 2B (2 minutes, 30°C), varying only the nature of the RNA molecule added to the pre-gapped/pre-unwound DNA substrate. See Materials and methods for description of the parameters plotted on the y-axis. Columns and associated error bars indicate the mean and standard deviation of three replicates. In both schematics, RNA molecules are outlined in orange, and DNA molecules are outlined in black. Circled ‘P’ indicates a 5′-phosphate. All sequences, when read right-side up, go from 5′ on the left to 3′ on the right. ‘OH’ indicates a hydroxyl. ‘Phos.’ indicates a phosphate. ‘Cyc. phos.’ indicates a 2′/3′-cyclic phosphate. ‘IVT/rz’ indicates that the RNA oligo was synthesized in an enzymatic *in vitro* transcription reaction, with ribozymes on both ends that cleaved to yield homogeneous termini. ‘Chem.’ indicates that the RNA oligo was chemically synthesized by a commercial source. The terms ‘Cas12a-like’ and ‘Cas9-like’ are descriptors only of each substrate’s R-loop topology (the end of the RNA next to the boundary of interest is a 3′ end or a 5′ end, respectively)—both kinds of substrates contain a Cas12a PAM and a Cas12a-like NTS gap.

**Figure 4—figure supplement 5.**
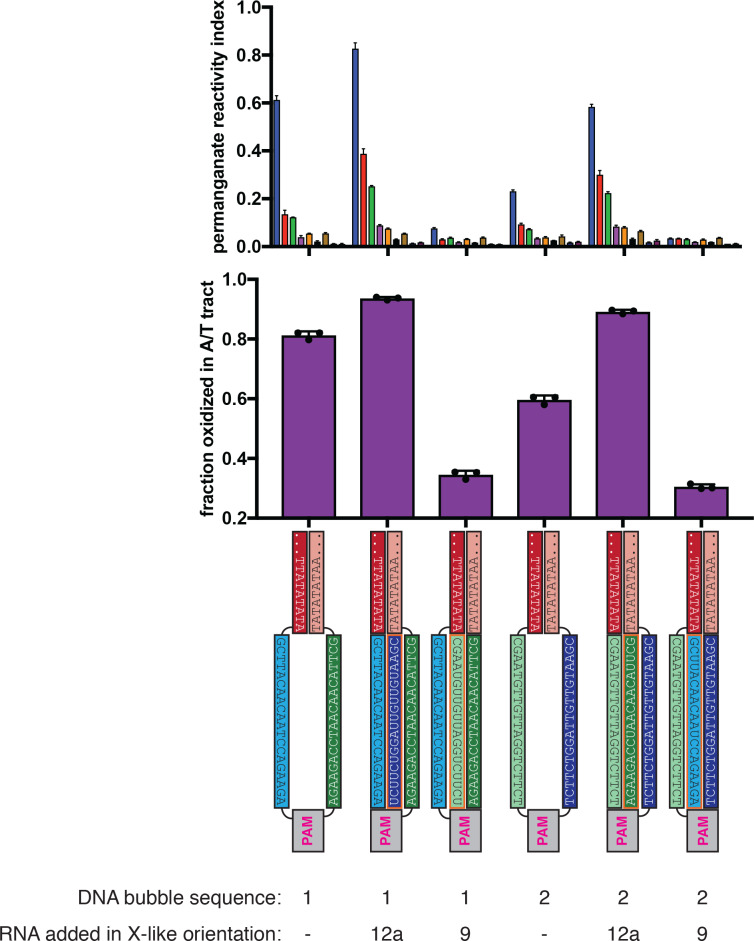
Asymmetry in R-loop flank stability is also a feature of intact R-loops. Permanganate experiments were conducted as in Figure 2B (2 minutes, 30°C). See Materials and methods for description of the parameters plotted on the y-axis. Columns and associated error bars indicate the mean and standard deviation of three replicates. In all schematics, RNA molecules are outlined in orange, and DNA molecules are outlined in black. All sequences, when read right-side up, go from 5′ on the left to 3′ on the right. These experiments provide the strongest point of comparison between the Cas12a-like and Cas9-like R-loop architecture, as the only component varied across conditions is which strand of an identical DNA bubble is hybridized to RNA (compare the cleaved R-loops, in which the position of the gap must be moved to the opposite strand, yielding slightly different baseline permanganate reactivity).

**Figure 4—figure supplement 6.**
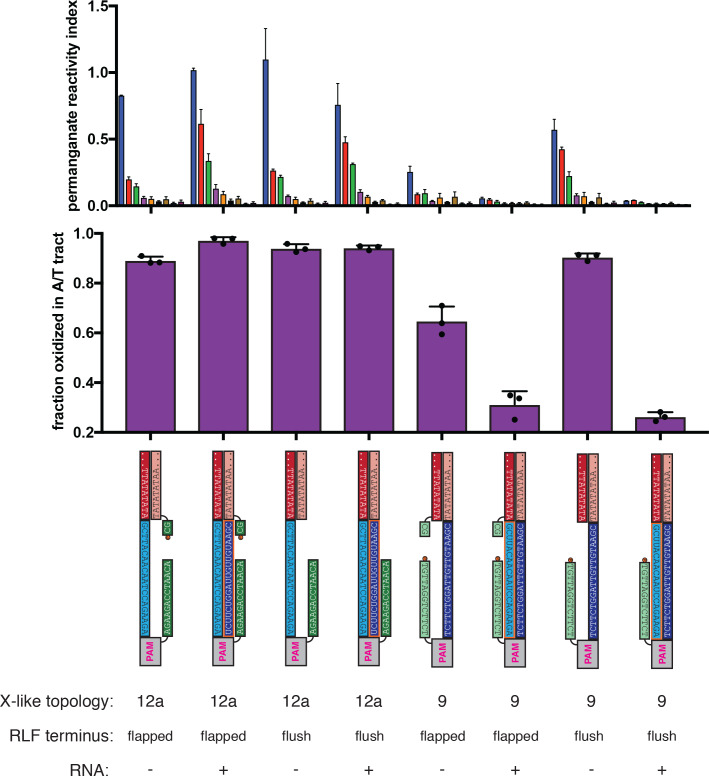
Effect of overhanging non-target-strand nucleotides on permanganate reactivity of the A/T tract in protein-free R-loops. Permanganate experiments were conducted as in Figure 2B (2 minutes, 30°C). See Materials and methods for description of the parameters plotted on the y-axis. Columns and associated error bars indicate the mean and standard deviation of three replicates. In all schematics, RNA molecules are outlined in orange, and DNA molecules are outlined in black. Circled ‘P’ indicates a 5′-phosphate. All sequences, when read right-side up, go from 5′ on the left to 3′ on the right. These experiments probe the role of the 2-nt NTS overhang (immediately adjacent to the R-loop flank) in distortion of the A/T tract. When present, this dinucleotide results in a ‘flapped’ R-loop flank (RLF) terminus, and when absent, the RLF terminus is ‘flush.’ The terms ‘(Cas)12a-like’ and ‘(Cas)9-like’ are descriptors only of each substrate’s R-loop topology (the end of the RNA next to the boundary of interest is a 3′ end or a 5′ end, respectively)—both kinds of substrates contain a Cas12a PAM and a Cas12a-like NTS gap. These results show that the presence of the overhang can affect the absolute magnitude of distortion, but the nature of the asymmetry is unaffected.

**Figure 4—figure supplement 7.**
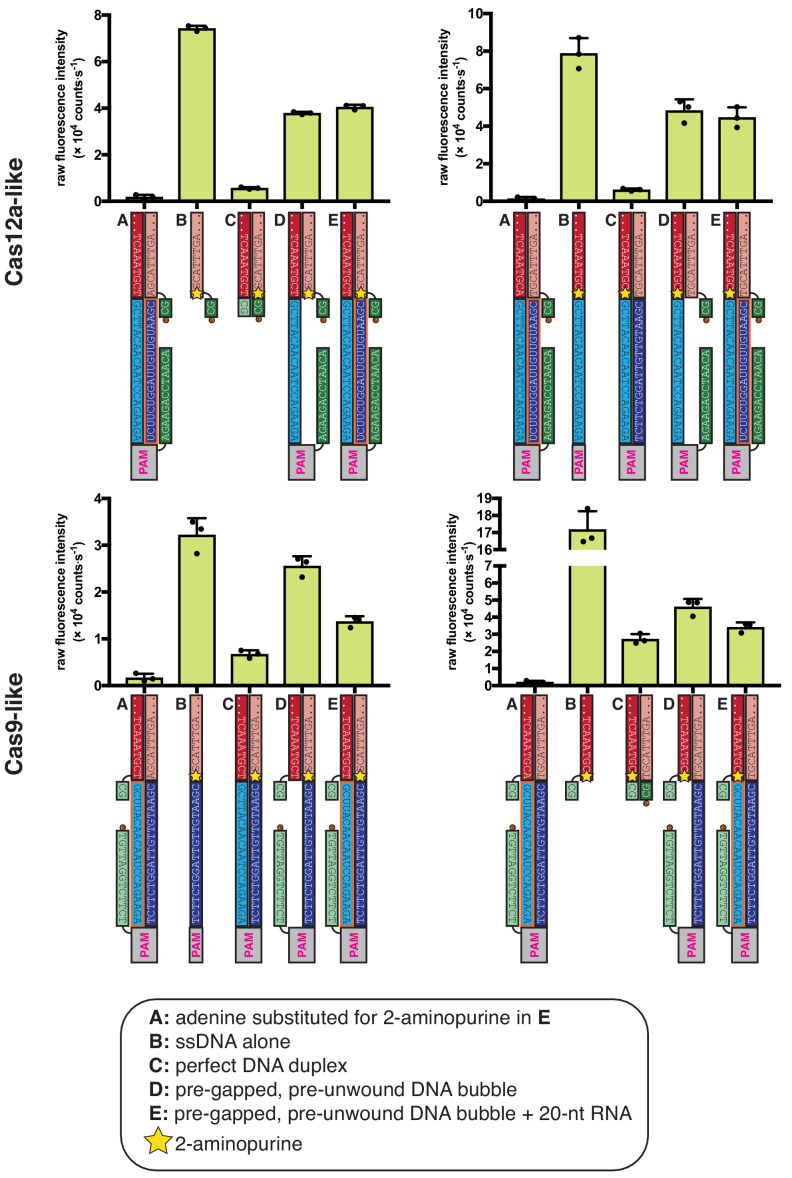
2-aminopurine fluorescence measurements confirm asymmetry in conformational dynamics of R-loop flanks. Columns and error bars show mean and standard deviation of three replicates. In all schematics, RNA molecules are outlined in orange, and DNA molecules are outlined in black. Circled ‘P’ indicates a 5′-phosphate. All sequences, when read right-side up, go from 5′ on the left to 3′ on the right. The terms ‘Cas12a-like’ and ‘Cas9-like’ are descriptors only of each substrate’s R-loop topology (the end of the RNA next to the boundary of interest is a 3′ end or a 5′ end, respectively)—both kinds of substrates contain a Cas12a PAM and a Cas12a-like NTS gap. The absolute values of 2-AP fluorescence intensity have a wide range across different DNA probes, likely due to local sequence context or, in the case of the ssDNA control in the bottom right panel, perhaps the stable population of a conformation that enhances 2-AP fluorescence. Given this variation, it is important to use the perfect DNA duplex (C) and the pre-gapped/pre-unwound DNA bubble (D) conditions as benchmarks—on the continuum from C to D, where does E lie? For condition E in the Cas12a-like topologies, the 2-AP fluoresces as if the RNA were absent. For condition E in the Cas9-like topologies, 2-AP fluorescence is quenched and approaches the intensity of the fully duplex control.

To interpret the reactivity of an R-loop mimic, we used the RNA-free DNA bubble control as a point of comparison. This bubbled DNA substrate reveals that permanganate reactions occur readily at the edge of the bubble, that is, the terminus of the DNA homoduplex (substrate C in Figure 4—figure supplement 1, also see Materials and methods). We therefore asked whether the adjacent RNA:DNA hybrid protects or sensitizes the DNA:DNA terminus as compared to the RNA-free control. The naïve prediction is that a DNA duplex terminus should become more stable (i.e., less prone to end fraying) when another duplex is stacked on top of it (Häse and Zacharias, 2016). It is thus surprising that the RNA oligonucleotide in this experiment sensitizes the DNA:DNA terminus to oxidation by permanganate. This result indicates that distortion of the R-loop flank is a phenomenon intrinsic to the R-loop boundary itself and that the protein only needs to hold the R-loop open to promote flexibility in the adjacent DNA.

### Duplex instability is not a feature of DNA in the RNA-5′ flank of R-loops

While Cas9 also conducts R-loop-dependent DNA cleavage, its R-loop topology is inverted with respect to that of Cas12a as a result of their opposing crRNA architectures—Cas12a crRNAs occur as 5′-repeat-spacer-3′, whereas Cas9 crRNAs occur as 5′-spacer-repeat-3′ (Figure 1B). Given the instability of the Cas12a R-loop flank (referred to as a 3′ R-loop flank because it contains a 3′ RNA terminus), we wondered whether the PAM-distal flank of the Cas9 R-loop (a 5′ R-loop flank) would also be unstable.

To test this question, we assayed flank distortion in an R-loop created by a catalytically inactive mutant of Cas9 from *Streptococcus pyogenes* (dCas9) and in the corresponding protein-free mimic (the non-target strand was pre-cleaved analogously to a Cas12a substrate). Remarkably, we found that the flank experienced nearly background oxidation levels both in the protein-bound R-loop (with dCas9 at a saturating concentration, Figure 4—figure supplement 2) and in the protein-free mimic, suggesting that unlike 3′ R-loop flanks, 5′ R-loop flanks are not naturally unstable (Figure 4A, Figure 4—figure supplement 1). The 5′ R-loop flank behaved consistently with expectations about coaxial duplex stacking, as the RNA oligonucleotide protected the DNA:DNA duplex terminus as compared to the bubbled control (Figure 4—figure supplement 1). Thus, an RNA:DNA hybrid can either stabilize or destabilize a juxtaposed DNA:DNA duplex terminus, depending on whether the hybrid terminus contains a 5′ RNA end or a 3′ RNA end, respectively. These results suggest a fundamental energetic difference in the conformational landscapes of 3′ versus 5′ R-loop flanks (Figure 4B).

The conformational difference between 3′ and 5′ R-loop flanks is intrinsic to strand polarity, as the trends in permanganate reactivity were robust to changes in nucleic acid sequence, end chemistry, and non-target-strand cleavage state (Figure 4—figure supplements 3–6). Additionally, we detected the same polarity dependence when we measured fluorescence intensity of a single 2-aminopurine nucleotide present at position 21 of the original protospacer sequence, indicating that the conformational difference is not an artifact of the permanganate reactivity assay or of the AT-rich sequence of the modified protospacer (Figure 4—figure supplement 7). Therefore, while Cas12a does not seem to actively destabilize the R-loop flank, the protein forms R-loops with the topology that natively yields a greater degree of flexibility in the region beyond the end of the crRNA.

### Differences in interhelical stacking energy may underlie asymmetric R-loop flank stability

**Figure 5.**
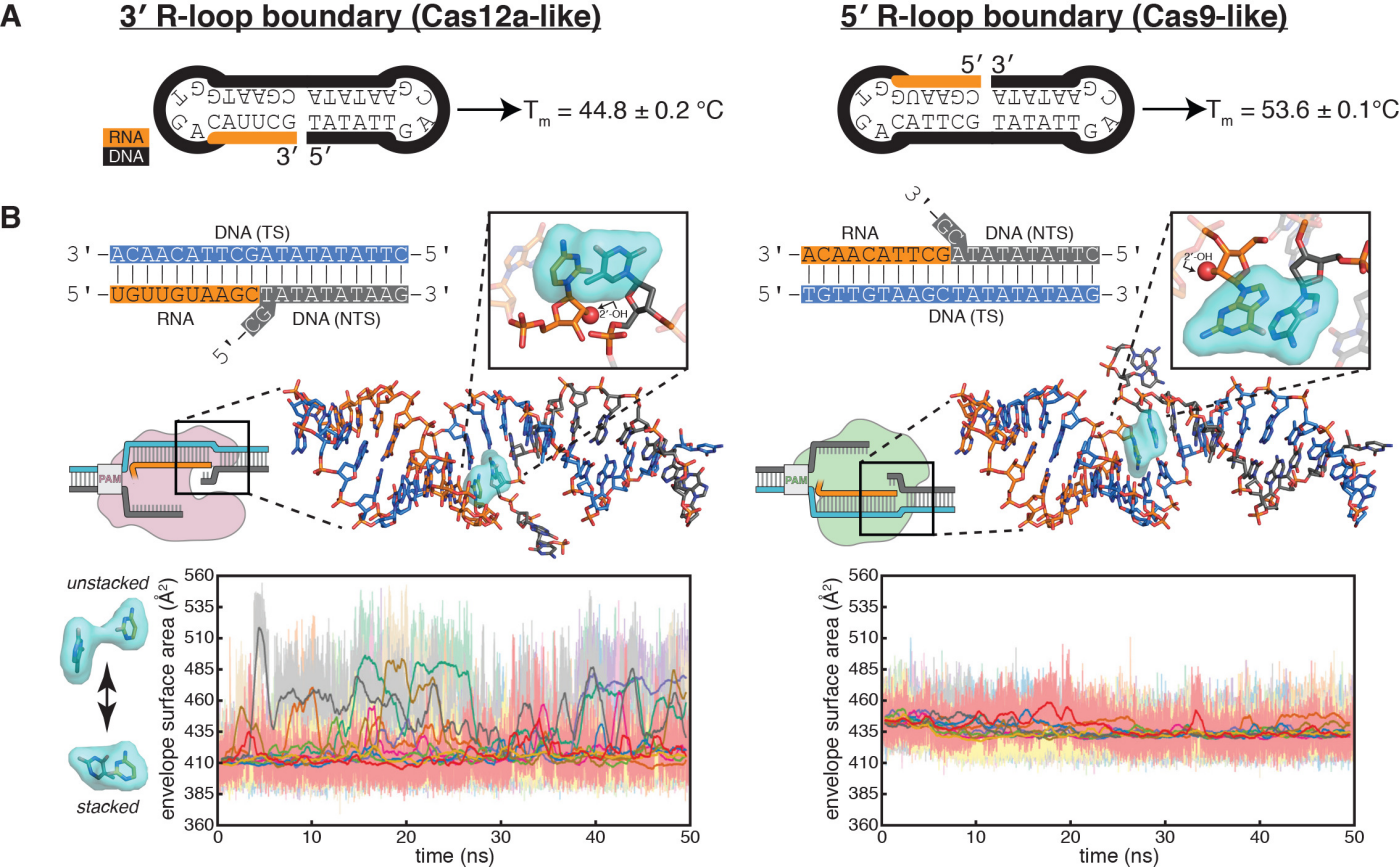
Energetics of base stacking at the R-loop boundary probed by optical measurements and molecular dynamics simulations. (**A**) Melting temperatures of nicked-dumbbell constructs that recapitulate each type of R-loop boundary, determined by monitoring absorbance of ultraviolet light while slowly cooling samples from 95°C to 2°C. Reported values show mean and standard deviation of three replicates. See Figure 5—figure supplement 1 for refolding curves and control constructs. (**B**) Molecular dynamics simulations reveal nucleobase unstacking in 3' R-loop boundaries but not in 5' R-loop boundaries. At the top left is a schematized version of the true structural model shown immediately below (this coaxially stacked conformation is the starting structure that was used for simulation); hydrogens were present in the simulated model and analyses but are omitted from representations here for clarity. The simulated model contained only the nucleic acid molecules shown in stick representation; the protein and remainder of the R-loop are drawn in a schematic only to orient the reader as to where the simulated structure would fit into a full DNA-bound CRISPR interference complex; the Cas9-orientation R-loop is drawn with a Cas12a-like NTS gap to reflect the simulated model. The inset is a closeup of the two nucleotides on the ‘flapped’ side of the junction in the structural model; the 2'-OH is shown as a red sphere. Envelope surface area (ESA) was determined by isolating two nucleobases of the interhelical stack—that on the RNA terminus and that stacked upon it from the NTS—and calculating the surface area of the volume they jointly occupy over the course of each trajectory (envelope shown in cyan). High ESA values reflect unstacking of nucleobases, whereas low ESA values reflect a stacked architecture similar to that of the starting conformation. Pale lines are absolute ESA values, and bold lines are moving averages (1-ns sliding window). Data from ten independent 50-ns trajectories are shown in different colors. Simulations of a second set of sequences are described in Figure 5—figure supplement 2. Figure 5—source data 1.Numerical data plotted in Figure 5 and accompanying figure supplements. Figure 5—source data 2.Example molecular dynamics trajectories.

Seeking a mechanistic explanation for the unequal stability of 3′ versus 5′ R-loop flanks, we hypothesized that the asymmetry may emerge from energetic differences in the coaxial stacking of a DNA homoduplex on either end of an RNA:DNA hybrid (Figure 5). Because interhelical junctions are known to explore both stacked and unstacked conformations in solution (Roll et al., 1998; Protozanova et al., 2004), they are expected to populate each state to a degree that depends on the free energy change associated with coaxial duplex stacking. Weaker stacking energy, then, encourages exploration of motions that initiate preferentially from unstacked duplex termini, such as DNA bending (Protozanova et al., 2004) and fraying (Häse and Zacharias, 2016).

**Figure 5—figure supplement 1.**
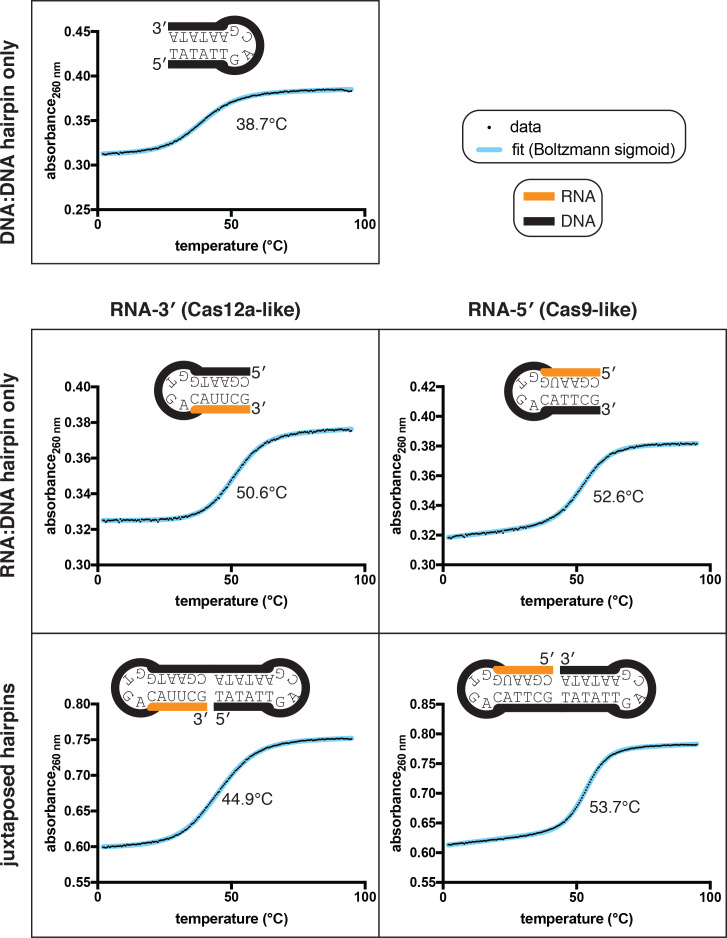
Thermal stability determination for nicked dumbbell substrates and their constituent hairpins. Data from representative replicates of refolding experiments (small black dots) are overlaid on a best-fit curve (thick blue line) comprising a Boltzmann sigmoid with inclined baselines (y = (m*x+b)+((n*x+c)-(m*x+b))/(1+exp((T_m_-x)/slope))). Because the dumbbells contain two separate duplexes that can, in principle, fold and unfold independently of each other, each of these molecules likely has more than two states. Thus, while there is no obvious visual sign of multiple transitions in the refolding curves, we did not attempt to extract thermodynamic parameters from the slope, and the T_m_ (written in the center of each plot) should only be used as a point of comparison rather than as a determinant of a defined conformational ensemble. While the two RNA:DNA hairpins have slightly different T_m_ values, the much larger discrepancy in T_m_ of the nicked dumbbells is probably mostly due to the nature of duplex juxtaposition.

**Figure 5—figure supplement 2.**
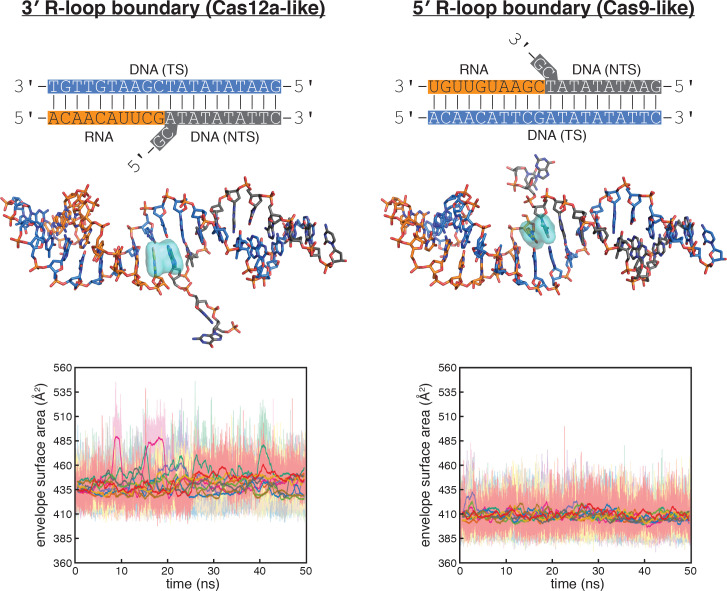
Molecular dynamics simulations of the Cas12a-like and Cas9-like interhelical junctions, Sequence 2. Schematics and data are presented as described in the legend to Figure 5B, which depicted simulation of Sequence 1. For Sequence 2, the nucleobases probed at the 3' R-loop boundary frequently exhibited unstacking or poorly stacked conformations (although to a lesser extent than in Sequence 1), while those probed at the 5' R-loop boundary were stably stacked over the course of the simulation. These results suggest that the difference in stacking instability detected for the interhelical junctions (with Sequence 1 or Sequence 2) are due to the difference in junction topology rather than the identities of the monitored nucleobases (pyrimidine/pyrimidine versus purine/purine).

To investigate whether differences in interhelical stacking energy could explain the difference in flank stability of the two R-loop topologies, we designed dumbbell substrates that reduced each type of R-loop boundary to a single chimeric oligonucleotide that contains both an RNA:DNA hybrid and a DNA:DNA homoduplex (Figure 5A, Figure 5—figure supplement 1). A stronger interhelical stack in these dumbbells should increase the thermal stability of the folded state (Erie et al., 1987). Through temperature-dependent hyperchromicity measurements, we determined that the RNA-5′ dumbbell (resembling the PAM-distal R-loop edge of Cas9) had a melting temperature 9°C higher than that of the RNA-3′ dumbbell (resembling the PAM-distal R-loop edge of Cas12a) (Figure 5A, Figure 5—figure supplement 1). The observed difference in melting temperature supports the idea that the resistance of 5′ R-loop flanks to permanganate oxidation may emerge from a more stable interhelical stack.

To probe the structural and energetic features of the interhelical stacks in atomic detail, we built models of coaxially stacked interhelical junctions of the two types: one containing an RNA-3′ end and one containing an RNA-5′ end. We performed a total of 500 nanoseconds of molecular dynamics simulation on each model, and we performed a second set of simulations on models of a different nucleotide sequence. Strikingly, the 3′ R-loop junctions frequently unstacked over the course of these short simulations, while the 5′ R-loop junctions remained relatively stable (Figure 5B, Figure 5—figure supplement 2). Observation of large-scale conformational transitions relevant to Cas12a-mediated DNA cleavage, such as fraying or bending events, would likely require much longer simulations (Leroy et al., 1988), but these short simulations suggest that the experimentally observed instability of 3′ R-loop flanks may arise from frequent unstacking events that comprise an early step in the DNA bending pathway. We speculate that different levels of interhelical stacking energy may emerge from certain sequence-independent features of the two duplexes juxtaposed in each type of R-loop boundary, such as helical geometry. The difference in the stacking equilibrium then leads to an unequal propensity for base pairing in the flanking DNA homoduplex. However, whether a difference in interhelical stacking energy can fully explain the observed difference in fraying propensity will require more detailed experimentation and analysis.

### Concluding remarks

**Figure 6.**
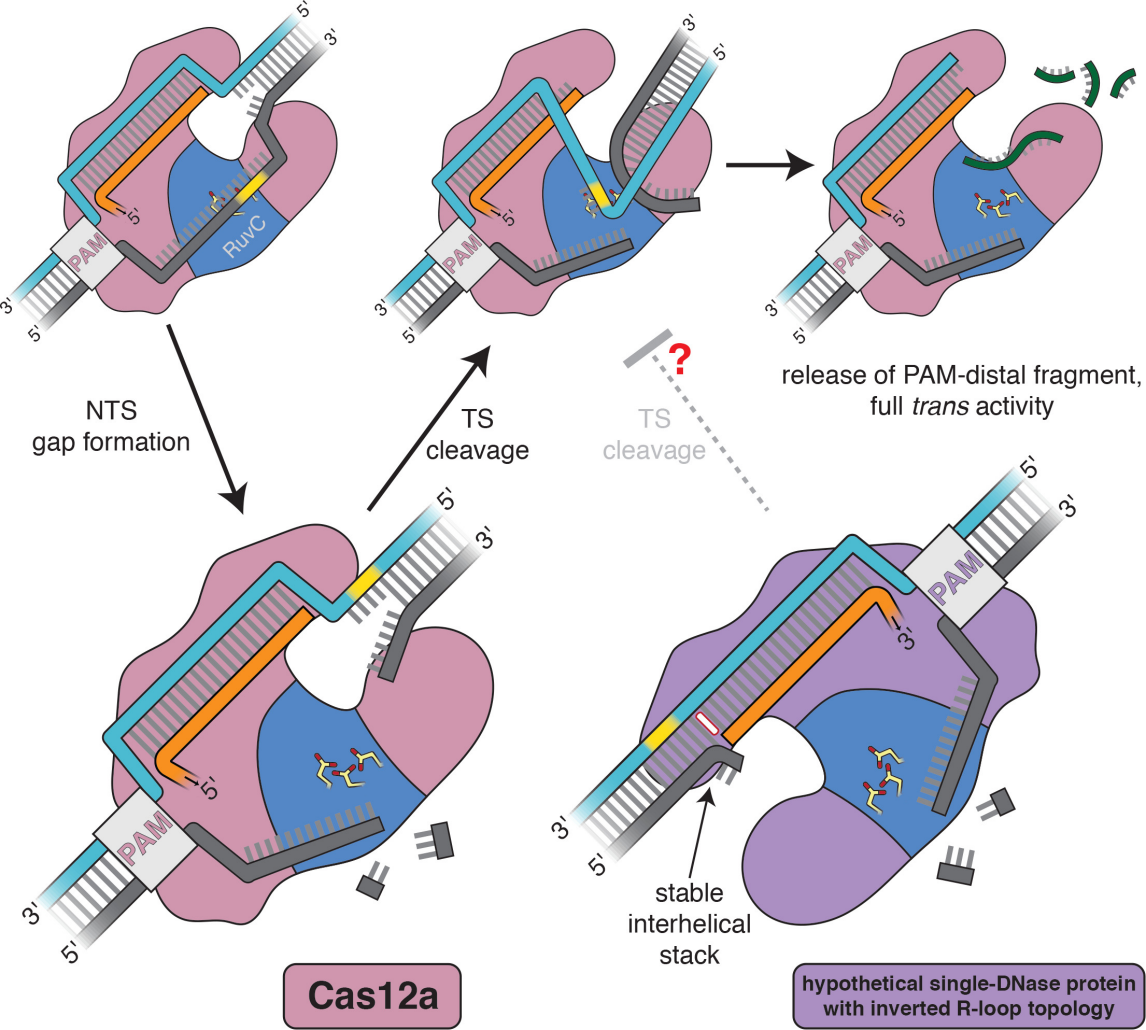
Model for the double-strand-break formation pathway of Cas12a and that of an analogous (hypothetical) enzyme with inverted R-loop topology. Scissile DNA tracts are shown in yellow. The stable interhelical stack in the hypothetical inverted complex is highlighted in white.

Our analyses have uncovered a fundamental asymmetry in the structure of the two kinds of R-loop flanks—RNA-3′ flanks exhibit a distorted conformation, whereas RNA-5′ flanks resemble standard B-form DNA (Figure 4B)—that is directly relevant to the mechanism of Cas12a-mediated DNA double-strand break formation. Specifically, the ability of the Cas12a RuvC active site to capture the target strand may rely on the active site’s proximity to the uniquely malleable 3′ R-loop flank, which relies in turn upon the topology of the Cas12a R-loop (Figure 6).

This peculiar asymmetry in nucleic acid conformational dynamics may also have consequences for non-CRISPR-associated R-loops. R-loops are common byproducts of transcription in eukaryotic genomes, and their dysregulation has been linked to a number of diseases (Crossley et al., 2019). It has been proposed that the mode of pathogenesis, in some cases, may emerge from the aberrant activity of general DNA repair nucleases on R-loop boundaries, whose scission leads to the formation of toxic double-strand breaks (Sollier et al., 2014). Notably, in the earliest biochemical exploration of this process, purified XPG (a human DNA repair nuclease) and nuclease P1 (a secreted fungal nuclease commonly used to selectively degrade single-stranded nucleic acid structures) robustly cleaved a 3′ R-loop flank but left the 5′ R-loop flank largely untouched (Tian and Alt, 2000). While this result was originally attributed to an idiosyncrasy of the tested sequence, our results suggest that the flank preference may have been polarity-dependent, not sequence-dependent. The experiments of Tian and Alt could represent an independent observation of the phenomenon dissected in the present work, implying that the asymmetric structure of R-loop boundaries may affect DNase sensitivity and genome stability in multiple domains of life.

Cas12a’s mode of dsDNA targeting contrasts with the established DNA cleavage mechanisms of other CRISPR interference complexes, which do not rely on instability in DNA flanking the R-loop. Cas9 forms R-loops with the topology that yields a stably base-paired PAM-distal R-loop flank (Figure 4A), but its second nuclease domain obviates the need to cleave outside the R-loop (Figure 1B). In type I CRISPR interference complexes, which have the same R-loop topology as Cas12a, the single-strand-specific DNase Cas3 is used to nick the displaced portion of the non-target strand after R-loop formation (Westra et al., 2012), similar to the initial non-target-strand nicking event in Cas12a. Cas3 eventually gains access to the PAM-proximal R-loop flank for processive DNA degradation, but, importantly, it uses an ATP-driven helicase to do so (Mulepati and Bailey, 2013). Thus, DNA cleavage in the 3′ R-loop flank of the Cas12a interference complex seems to be a solution that maximizes the utility of its minimal enzymatic machinery.

Intriguingly, all known DNA-targeting Cas12 enzymes, which are taxonomically defined by their minimal enzymatic machinery (a single RuvC DNase domain), form R-loops of the topology that allows target-strand cleavage within a PAM-distal 3′ R-loop flank (Yang et al., 2016; Liu et al., 2019; Yan et al., 2019; Karvelis et al., 2020) (for target-strand cleavage properties of Cas12i, see Appendix 2). Given the importance of DNA bending in enabling a single RuvC active site to form double-strand breaks, we propose that the universal type V R-loop topology may be the product of biophysically imposed selective pressures. If a single-DNase Cas enzyme were to form R-loops of the Cas9-like topology, the target strand would be cut, by analogy, within a 5′ R-loop flank. Our results indicate that 5′ R-loop flanks resist fraying and bending, which may lead to slower target-strand cleavage in the hypothetical topology-inverted enzyme (Figure 6). We hypothesize that, under the strong evolutionary pressure for immune responses that efficiently produce double-strand breaks (Heitman et al., 1999), ancestral enzymes with PAM-distal 3′ R-loop flanks outperformed topology-inverted variants, perhaps leading to the modern ubiquity of the Cas12a-like R-loop architecture in type V enzymes. Importantly, the extant diversity of Cas12 proteins likely did not emerge from a single CRISPR-associated ancestor but rather from many distinct transposon-encoded TnpB proteins (Koonin et al., 2017; Makarova et al., 2020), suggesting that several independent evolutionary trajectories have converged on the same R-loop topology.

While these evolutionary speculations cannot be experimentally verified at present, our findings also provide valuable mechanistic information that will support the development of Cas12-based genome manipulation technologies. For instance, it has been proposed that Cas12-family proteins could be used as RNA-guided DNA ‘nickases’ through selective removal of target-strand cleavage activity, but those nickases reported so far are catalytically inefficient and do not totally eliminate target-strand cutting (Appendix 2) (Yamano et al., 2016; Yan et al., 2019). Our permanganate reactivity results revealed that target-strand distortion in Cas12a-generated R-loops, as probed by our experimental techniques, can be explained by conformational dynamics intrinsic to the nucleic acids (Figure 4A). Thus, engineering a type V CRISPR nickase that performs fast non-target-strand cleavage but undetectable target-strand cleavage will likely require modifications that shield or distance the RuvC active site from the intrinsically labile 3′ R-loop flank. Looking further ahead, if *de novo* design of Cas-like RNA-guided nucleases (Rauch et al., 2019) someday enters the realm of dsDNA-targeting, knowledge of the asymmetry in R-loop flank stability may be useful in tailoring the architecture of a designed enzyme to a specific application. Our results suggest that placing a DNase domain near a 3′ R-loop flank would encourage fast target-strand cleavage (for applications requiring double-strand breaks), while placing one near a 5′ R-loop flank would likely inhibit target-strand cleavage (for nickase applications). Complex engineering feats will become more attainable through continued investigation into the functional, structural, and evolutionary features of natural dsDNA-targeting CRISPR systems.

## Materials and methods

**Key resources table:** 

Reagent type (species) or resource	Designation	Source or reference	Identifiers	Additional information
Recombinant DNA reagent	pMBP-AsCas12a expression plasmid	Chen et al. (2018)	RRID:Addgene_113430	See Supplementary file 1 for the sequences of all plasmids and oligonucleotides
Peptide, recombinant protein	T4 polynucleotide kinase	New England Biolabs	M0201S	
Peptide, recombinant protein	T4 RNA ligase 2	New England Biolabs	M0239S	
Peptide, recombinant protein	AsCas12a protein	this paper		All Cas proteins purified from *E. coli* BL21 Star(DE3) cells
Chemical compound, drug	[γ-^32^P]-ATP	PerkinElmer	BLU502A001MC	
Chemical compound, drug	potassium permanganate	Sigma-Aldrich	223468	
Software, algorithm	ImageQuant TL	GE Healthcare	RRID:SCR_014246	

### Protein expression and purification

Expression plasmids were cloned as previously described (Chen et al., 2018; Knott et al., 2019). Briefly, protein-coding DNA segments were shuttled into custom pET-based vectors by Gibson assembly, and catalytic mutants were generated through site-directed mutagenesis by polymerase chain reaction (PCR) with blunt-end ligation (plasmid sequences are in Supplementary file 1). The parent plasmid from which the Cas12i1 expression plasmid was cloned (pET28a-mH6-Cas12i1) was a gift from Arbor Biotechnologies (Addgene plasmid #120882). AsCas12a protein expression and purification was performed as previously described (Chen et al., 2018) with the following modifications. The cells used for expression were *E. coli* BL21 Star(DE3). Lysis buffer was 50 mM HEPES (pH 7.5), 500 mM NaCl, 1 mM TCEP, 0.5 mM PMSF, 10 tablets/L cOmplete EDTA-free protease inhibitor cocktail (Roche), and 0.25 mg/mL chicken egg white lysozyme (Sigma-Aldrich). Ni-NTA wash buffer was 50 mM HEPES (pH 7.5), 500 mM NaCl, 1 mM TCEP, 5% glycerol, 20 mM imidazole. Ni-NTA elution buffer was 50 mM HEPES (pH 7.5), 500 mM NaCl, 1 mM TCEP, 5% glycerol, 300 mM imidazole. TEV protease cleavage was performed overnight while dialyzing against dialysis buffer (50 mM HEPES (pH 7.5), 250 mM NaCl, 1 mM TCEP, 5% glycerol). Low-salt ion exchange buffer was 50 mM HEPES (pH 7.5), 250 mM KCl, 1 mM TCEP, 5% glycerol. High-salt ion exchange buffer was 50 mM HEPES (pH 7.5), 1 M KCl, 1 mM TCEP, 5% glycerol. Gel filtration buffer was 20 mM HEPES (pH 7.5), 200 mM KCl, 1 mM TCEP, 5% glycerol. Cas12i1 was expressed and purified with the same protocol as for AsCas12a. FnCas12a and SpCas9 were expressed with the same protocol as for AsCas12a. Purification of FnCas12a only differed from that of AsCas12a in the lack of a TEV protease cleavage step, as the FnCas12a expression construct lacked a cleavable tag. Purification of SpCas9 only differed from that of AsCas12a in the ion exchange buffers and gel filtration buffer, which contained 10% glycerol instead of 5% glycerol. Each protein was expressed and purified once to create a single set of stock aliquots that were used for all experiments reported within this manuscript.

### *In vitro* transcription of RNA

Guide RNAs and some short RNAs used in R-loop mimics were produced by *in vitro* transcription (see Supplementary file 1). Double-stranded DNA templates for T7 RNA polymerase transcription were assembled from several overlapping DNA oligonucleotides (synthesized by IDT) by PCR. Transcription occurred in 40 mM Tris-Cl (pH 7.9 at 25°C), 25 mM MgCl_2_, 10 mM dithiothreitol, 0.01% (v/v) Triton X-100, and 2 mM spermidine, with 5 mM of each NTP and 100 µg/mL T7 RNA polymerase. Transcription was allowed to proceed for 2.5 hr at 37°C. Cas9 sgRNAs were transcribed with a hammerhead ribozyme on the 5′ end to allow an arbitrary sequence on the 5′ end of the final sgRNA. Cas12a and Cas12i crRNAs were transcribed with a hepatitis delta virus ribozyme on the 3′ end to avoid the impurities associated with non-templated nucleotide addition in the final crRNA. 20-nt spacers used in R-loop mimics were transcribed with both a 5′-HHrz and a 3′-HDVrz. For those RNA transcripts that contained hammerhead ribozyme, which is prone to misfolding, an additional 5 mM MgCl_2_ was added to the transcription products, and the reaction was placed on a thermocycler for iterative annealing ({80°C for 2 min, 37°C for 10 min} x 8, hold at 10°C). DNA in all transcription reactions was then digested with DNase I (RQ1 RNase-Free DNase, Promega) (0.05 U/µL, manufacturer’s units) for 30 min at 37°C. RNA fragments released by the ribozymes were then purified by denaturing PAGE (10% acrylamide:bis-acrylamide 29:1, 7 M urea, 0.5X TBE), ethanol-precipitated, and resuspended in RNA storage buffer (0.1 mM EDTA, 2 mM sodium citrate, pH 6.4).

### Nucleic acid and interference complex preparation

All DNA oligonucleotides and some RNA oligonucleotides (as indicated in Supplementary file 1) were ordered from Integrated DNA Technologies. DNA oligonucleotides used in biochemical experiments were PAGE-purified in house and resuspended in water. A_260_ was measured on a NanoDrop (Thermo Scientific), and concentration was estimated according to extinction coefficients determined by OligoCalc (Kibbe, 2007). DNA substrates were annealed in annealing buffer (10 mM Tris-Cl, pH 7.9 at 25°C, 50 mM KCl, 1 mM EDTA) by heating to 95°C and cooling to 25°C over the course of 40 min on a thermocycler. This annealing reaction was always performed with 40 nM radiolabeled DNA strand. When the DNA substrate comprised just two complementary strands, the unlabeled (complementary) strand was included in the annealing reaction at 80 nM. When the DNA substrate comprised more than two distinct strands, strands with the same sense as the radiolabeled strand were included at 80 nM, and strands complementary to the radiolabeled strand were included at 60 nM. For protein-free R-loop mimics, the RNA (or other spacer mimic) was included in the annealing reaction at 400 nM, to match the guide RNA concentration of a protein-containing experiment. The substrate concentrations reported in figure legends refer to the concentration of the radiolabeled strand. All crRNA and sgRNA molecules were annealed in RNA storage buffer (0.1 mM EDTA, 2 mM sodium citrate, pH 6.4) prior to use (80°C for 1 min, then moved directly to ice). To form CRISPR surveillance complexes (Cas protein + guide RNA), crRNA or sgRNA was combined with Cas protein (both at 2X final concentration in 1X reaction buffer) and allowed to equilibrate for 5 min at 37°C. To form complete interference complexes (Cas protein + guide RNA + DNA target), 1 vol of 2X surveillance complex (in 1X reaction buffer) was combined with 1 vol of 2X DNA substrate (in 1X reaction buffer) and allowed to equilibrate for 5 min at 37°C (if applicable).

### DNA oligonucleotide radiolabeling

Standard 5′ radiolabeling was performed with T4 polynucleotide kinase (New England Biolabs) at 0.2 U/µL (manufacturer’s units), 1X T4 PNK buffer (New England Biolabs), 400 nM DNA oligonucleotide, and 200 nM [γ-^32^P]-ATP (PerkinElmer) for 30 min at 37°C, followed by a 20-min heat-killing incubation at 65°C. Radiolabeled oligos were then buffer exchanged into water using a Microspin G-25 spin column (GE Healthcare). For 3′ radiolabeling (see Figure 2—figure supplement 1), which was based on the mechanistic work of Nandakumar and Shuman (2004), the DNA oligonucleotide to be radiolabeled was synthesized by Integrated DNA Technologies with two modifications: the sugar moiety of the 3′-most nucleotide was a ribose, and the sugar moiety of the penultimate nucleotide was a 2′-O-methyl ribose. A ‘phosphate shuttle’ RNA oligonucleotide underwent a high-yield 5′-radiolabeling procedure (0.5 U/µL T4 PNK, 1X T4 PNK buffer, 1 µM RNA oligonucleotide, 500 nM [γ-^32^P]-ATP, 2 hr at 37°C, 20 min at 65°C, buffer exchanged into water). A T4 RNA ligase 2 substrate was then formed by hybridizing the phosphate shuttle (363 nM) and the DNA oligo to be radiolabeled (303 nM) to a ‘splint’ RNA (333 nM) in annealing buffer (10 mM Tris-Cl, pH 7.9 at 25°C, 50 mM KCl, 1 mM EDTA) by heating to 95°C and cooling to 25°C over the course of 40 min on a thermocycler. T4 RNA ligase 2 (New England Biolabs) at 0.5 U/µL (manufacturer’s units), 1X T4 RNA ligase 2 reaction buffer, and 1 mM MgCl_2_ were added to this annealed structure. Ligation was allowed to proceed overnight at 37°C. The phosphate shuttle and splint RNA oligonucleotides were degraded by adding 150 mM NaOH and incubating at 95°C for 10 min. The degradation reaction was stopped by adding a stoichiometric amount of HCl and placing on ice. The 3′-radiolabeled DNA oligonucleotide was then buffer-exchanged into 20 mM Tris-Cl (pH 7.9 at 25°C) using a Microspin G-25 spin column. This protocol has ~75% yield in terms of transfer of radioactivity from the phosphate shuttle RNA to the DNA oligonucleotide 3′ end. The hot hydroxide treatment causes slight accumulation of depurination products, but such products comprise a trivial fraction of the total population of radiolabeled DNA and do not interfere with downstream analysis. See Supplementary file 1 for the identities and sequences of oligonucleotide reagents used in 3′-radiolabeling procedures.

### Permanganate reactivity experiments

The permanganate footprinting protocol was based on Pul et al. (2012). In 40 µL permanganate reaction buffer (20 mM Tris-Cl, pH 7.9 at 25°C, 150 mM KCl, 5 mM MgCl_2_), DNA (10 nM radiolabeled strand, 20 nM unlabeled strand), guide RNA or spacer mimic (100 nM), and protein (120 nM) were combined (omitting components as indicated for each experiment) and allowed to equilibrate to 30°C for >5 min. 4 µL 160 mM KMnO_4_ (solution prepared in permanganate reaction buffer immediately before reaction) was added and allowed to react for 2 min (unless otherwise indicated) at 30°C. Reactions were quenched with 4.8 µL β-mercaptoethanol and moved to ice. 5.3 µL 500 mM EDTA was added. 45.9 µL water was added, and samples were extracted once with 100 µL 25:24:1 phenol:chloroform:isoamyl alcohol (pH 8) in 5PRIME Phase Lock Heavy tubes (Quantabio). The aqueous phase was isolated and combined with 10 µL 3 M sodium acetate (pH 5.2), 1 µL GlycoBlue coprecipitant (Invitrogen), and 300 µL ethanol, and left at −20°C for >2 hr. DNA was precipitated by centrifugation, and supernatant was decanted. Wet ethanol pellets were resuspended in 10% piperidine and incubated at 90°C for 30 min. Solvent was evaporated in a SpeedVac (ThermoFisher Scientific). Approximate yield was determined by measuring radioactivity of the pellet-containing tube in a benchtop radiation counter (Bioscan QC-4000), and pellets were resuspended in an appropriate volume of loading solution (50% water, 50% formamide, 0.025% w/v bromophenol blue) to normalize signal across samples prior to resolution by denaturing PAGE. Oligonucleotide identities and sequences are shown in Supplementary file 1.

### Denaturing polyacrylamide gel electrophoresis and phosphorimaging

Radiolabeled DNA oligonucleotides were denatured (95°C in 50% formamide for 3 min) and resolved on a denaturing polyacrylamide gel (15% acrylamide:bis-acrylamide 29:1, 7 M urea, 0.5X TBE). Gels were dried (4 hr, 80°C) on a gel dryer (Bio-Rad) and exposed to a phosphor screen. Phosphor screens were imaged on an Amersham Typhoon phosphorimager (GE Healthcare). Phosphorimages were quantified using ImageQuant software (GE Healthcare).

### Electrophoretic mobility shift assay and filter-binding assay

In both kinds of binding assays, complexes were formed in 1X binding buffer (20 mM Tris-Cl, pH 7.9 at 25°C, 150 mM KCl, 5 mM MgCl_2_, 1 mM TCEP, 50 µg/mL heparin, 50 µg/mL bovine serum albumin, 5% glycerol). Cas protein was first diluted in series in binding buffer, added to a fixed concentration of guide RNA, and incubated at 37°C for 5 min, then 25°C for 25 min. This complex was then added to the radiolabeled DNA probe and incubated at 37°C for 5 min, then 25°C for 1 hr. When the titrant was crRNA instead of Cas protein, the Cas12a:crRNA complex was incubated at 25°C for 30 min, added to DNA probe, and incubated at 25°C for an additional 1 hr. For the EMSA, samples were then resolved on a native PAGE gel (8% acrylamide:bis-acrylamide 29:1, 0.5X TBE, 5 mM MgCl_2_), which was dried and phosphorimaged. For the filter-binding assay, HT Tuffryn (Pall), Amersham Protran, and Amersham Hybond-N+ (GE Healthcare) membranes were equilibrated in 1X membrane wash buffer (20 mM Tris-Cl, pH 7.9 at 25°C, 150 mM KCl, 5 mM MgCl_2_, 1 mM TCEP, 5% glycerol) and assembled on a vacuum dot-blot apparatus. Radioactive samples were applied to the membranes, and each spot was washed once with 40 µL 1X wash buffer. Membranes were air-dried and phosphorimaged. For assays testing complex assembly in calcium-containing buffer, 5 mM CaCl_2_ was substituted for MgCl_2_ in the binding buffer. Oligonucleotide identities and sequences are shown in Supplementary file 1.

### Enzymatic DNA cleavage assays

To initiate DNA cleavage, 1 vol of 2X surveillance complex, *trans*-active interference complex, or other nuclease (in 1X cleavage buffer) was combined with 1 vol of 2X radiolabeled DNA substrate (in 1X cleavage buffer) at 37°C (unless specified otherwise). For Cas12a, standard cleavage buffer was 10 mM Tris-Cl, pH 7.9 at 25°C, 150 mM KCl, 5 mM MgCl_2_, 1 mM TCEP. ‘Calcium-containing’ cleavage buffer contained 5 mM CaCl_2_ instead of MgCl_2_. For Cas12i1, cleavage buffer was 50 mM Tris-Cl, pH 8.0 at 25°C, 50 mM NaCl, 10 mM MgCl_2_. For S1 nuclease (ThermoScientific), cleavage buffer was the 1X reaction buffer provided by the manufacturer. At each timepoint, 1 vol of reaction was quenched with 1 vol of 2X quench buffer (94% formamide, 30 mM EDTA, 0.025% w/v bromophenol blue). For reactions catalyzed by Cas12i1, the 2X quench buffer also included 400 µg/mL heparin and 0.2% sodium dodecyl sulfate to prevent aggregation in gel wells. For ‘t = 0’ timepoints, surveillance complex was first added to quench buffer and mixed, followed by addition of DNA substrate. Products were then resolved by denaturing PAGE and phosphorimaging. Oligonucleotide identities and sequences are shown in Supplementary file 1.

### 2-aminopurine fluorescence intensity measurements

All oligonucleotides used in these experiments were first ethanol-precipitated and resuspended to remove impurities from commercial synthesis that interfered with the optical spectra of interest. Oligonucleotides were combined to their final concentration in 1X nucleic acid spectroscopy buffer (10 mM K_2_HPO_4_/KH_2_PO_4_, pH 6.7, 150 mM KCl, 0.1 mM EDTA) and annealed on a thermocycler (95°C to 25°C over the course of 40 min). Final concentrations were 5 µM 2-AP-containing oligonucleotide, 5.5 µM complementary oligonucleotide (if present), 6 µM same-stranded oligonucleotide (if present), and 6 µM RNA oligonucleotide (if present). Samples were placed in a 1.5-mm fluorescence cuvette (Hellma Analytics) and allowed to equilibrate inside the temperature-controlled (30°C) cell of a QuantaMaster spectrofluorometer (Photon Technology International) for 3 min. The lamp power was set to 74 W, and the slit widths were set as follows (excitation slit 1: 0.5 mm; excitation slit 2: 1 mm; emission slit 1: 2 mm; emission slit 2: 0.9 mm). Fluorescence intensity (λ_ex_=310 nm (4 nm bandpass), λ_em_=370 nm (3.6 nm bandpass)) was measured for 30 s, and the average across those 30 s was reported. Oligonucleotide identities and sequences are shown in Supplementary file 1.

### Dumbbell/hairpin melting temperature determination

All oligonucleotides used in these experiments were first ethanol-precipitated to remove impurities from commercial synthesis that interfered with the optical spectra of interest. Oligonucleotides were resuspended to an estimated 2.25 µM (the extinction coefficient of a highly stacked nucleic acid structure is difficult to estimate, but the unimolecular physical processes being probed are concentration-independent, in theory) in 1X nucleic acid spectroscopy buffer (10 mM K_2_HPO_4_/KH_2_PO_4_, pH 6.7, 150 mM KCl, 0.1 mM EDTA). Samples were placed in a 1-cm CD-grade quartz cuvette (Starna Cells) with a stir bar and cap, which was placed in the sample cell of a temperature-controlled spectrophotometer (Cary UV-Vis 100). An equivalent cuvette containing only nucleic acid spectroscopy buffer was placed in the reference cell. The stir apparatus was turned on, the block was heated to 95°C, and the samples were allowed to equilibrate for 3 min. The system was cooled to 2°C at 1 °C/minute, collecting an A_260_ measurement every 0.5°C (averaging time = 2 s, slit bandwidth = 1 nm). Refolding of the Cas12a-like dumbbell at a slower temperature ramp rate (0.3 °C/minute) yielded results similar to those pictured, indicating that the faster ramp rate (1 °C/minute) was still slow enough that the absorbance measurements approached their equilibrium values. Oligonucleotide identities and sequences are shown in Supplementary file 1.

### Molecular dynamics simulations

The starting conformation of each junction was based on a relaxed structure of a chimeric RNA:RNA/DNA:DNA duplex. A 10-bp A-form RNA:RNA duplex and a 10-bp B-form DNA:DNA duplex were each built in x3DNA (Lu and Olson, 2003). These duplexes were manually placed in a coaxially stacked conformation using PyMOL (DeLano, 2010), and the junction was sealed on both strands using Coot (Emsley et al., 2010) (with slight adjustment of neighboring dihedrals to accommodate the introduced bonds). Using VMD (Humphrey et al., 1996), this system was solvated with TIP3 waters (Jorgensen et al., 1983) to a cube that stretched 15 Å past each edge of the nucleic acid duplex in its widest dimension. 150 mM NaCl and 5 mM MgCl_2_ were added to the solvent using VMD. The system was minimized for 2000 steps with the nucleic acid atoms held fixed, then minimized for an additional 2000 steps while allowing all atoms to move. The system was then equilibrated for 1 ns, and the final structure of the chimeric duplex was used as the basis for building the two kinds of junctions.

For the RNA-3′ junction, the bond between the internal 3′ end of the RNA tract and the internal 5′ end of the DNA tract was removed, and a 2-nt DNA flap was modeled in an arbitrary conformation. On the opposite strand, all ribonucleotides were changed to 2′-deoxyribonucleotides, and uracils were changed to thymines. The RNA-5′ junction was built analogously. For both junctions, the outer termini of each duplex contained 5′-OH and 3′-OH. The internal DNA flap contained a 5′-phosphate (RNA-3′ junction) or a 3′-OH (RNA-5′ junction), in keeping with the chemical products of RuvC-catalyzed DNA cleavage. The internal RNA end contained a 3′-OH (RNA-3′ junction) or a 5′-OH (RNA-5′ junction). These systems were solvated and minimized as before. The systems were then equilibrated for 1 ns with the nucleic acid atoms held fixed. This system served as the starting state for 10 separate production trajectories that were each run for 50 ns with all atoms free. All equilibration and production runs were carried out in the NPT ensemble at a temperature of 300 K and pressure of 1 atm.

The simulations were performed on XSEDE computing resources (Towns et al., 2014) using the NAMD (Phillips et al., 2005) package with the CHARMM36m forcefield (Huang et al., 2017) and an integration timestep of 2 fs. The Particle Mesh Ewald approximation was used to calculate long-range electrostatic interactions (Darden et al., 1993) with a grid size of 1 Å. Van der Waals interactions were truncated at 12 Å. Hydrogen atoms bonded to heavy atoms were constrained with the ShakeH algorithm (Ryckaert et al., 1977). The Langevin thermostat was used to control the temperature with a damping coefficient of 1/ps, applied to non-hydrogen atoms. Pressure was controlled with the Nose-Hoover Langevin method (Martyna et al., 1994; Feller et al., 1995), with a Langevin piston period of 200 fs and a piston decay time of 50 fs.

For each trajectory, the coordinates of the two nucleobases at the junction on the flapped strand were isolated for further analysis at a sampling rate of 1/ps. Envelope surface area (ESA), defined as the solvent-exposed surface area of the two isolated nucleobases, was determined in PyMOL and serves as a metric of the degree of base stacking (bases that are well-stacked have a low ESA, whereas bases that are unstacked have a high ESA). All figures were prepared in PyMOL.

### DNA size standard preparation

To identify a known specific cleavage site within the protospacer, radiolabeled DNA duplexes were digested with TseI (New England Biolabs) (0.025 U/µL final concentration, manufacturer’s units) in DNase buffer (10 mM Tris-Cl, pH 7.9 at 25°C, 150 mM KCl, 5 mM MgCl_2_, 1 mM TCEP) for 10 min at 65°C. To generate a single-nucleotide ladder, the same radiolabeled DNA oligonucleotides were separately digested with nuclease P1, DNase I, *trans*-active AsCas12a, and T5 exonuclease (which all leave 5′ phosphate and 3′-OH on their cleavage products, chemically equivalent to products of Cas12a *cis* cleavage), and products were pooled at a 1:1:1:1 ratio (T5 exonuclease was not used for 5′-radiolabeled oligonucleotides) prior to loading on the gel. Nuclease P1 (New England Biolabs) digests were performed with single-stranded radiolabeled DNA oligonucleotide and 0.5 U/µL enzyme (manufacturer’s units) in DNase buffer for 3 min at 37°C. DNase I (RQ1 RNase-Free DNase, Promega) digests were performed with radiolabeled DNA duplex and 0.01 U/µL enzyme (manufacturer’s units) in DNase buffer for 3 min at 37°C. AsCas12a digests were performed with single-stranded radiolabeled DNA oligonucleotide, 100 nM AsCas12a, 120 nM crRNA, and 50 nM pre-cleaved DNA activator in DNase buffer for 5 min at 37°C. T5 exonuclease (New England Biolabs) digests were performed with radiolabeled DNA duplex and 0.01 U/µL enzyme (manufacturer’s units) in DNase buffer for 10 min at 37°C. All reactions were stopped by addition of 1 vol 2X quench buffer (94% formamide, 30 mM EDTA, 0.025% w/v bromophenol blue).

### Steady-state Cas12a *trans* DNA cleavage kinetic analysis

Kinetics of ssDNA cleavage were assessed by monitoring the rate of dequenching of a fluorophore-DNA-quencher substrate, as in Chen et al. (2018). Briefly, *trans*-active holoenzyme (final concentrations: 100 nM AsCas12a, 120 nM crRNA, 10 nM pre-cleaved duplex activator) was added to various concentrations of fluorophore-DNA-quencher substrate, and fluorescence (excitation filter: 485 nm/20 bandpass, emission filter: 528 nm/20 bandpass) was monitored over time at 37°C on a Cytation5 fluorescence plate-reader (BioTek). V_0_ was determined as (slope_all components_ - slope_no DNA activator_)×(fluorescence intensity:[product] conversion factor). The fluorescence intensity:[product] conversion factor was determined empirically for each separate concentration of fluorescent reporter (by equilibrium titration of purified cleaved/uncleaved reporter), as the relationship departed from linearity at higher substrate concentrations. Oligonucleotide identities and sequences are shown in Supplementary file 1.

### Model fitting

All models were fit by the least-squares method in Prism 7 (GraphPad Software). The model used for each dataset is described in the corresponding figure legend.

### Analysis and interpretation of permanganate reactivity data

In this work, data describing permanganate reactivity are presented in three ways:

1. Raw phosphorimages of denaturing PAGE analysis of DNA substrates treated with permanganate and piperidine.
2. ‘Permanganate reactivity index’ (PRI) of individual thymine nucleobases. This metric is determined from the raw phosphorimages. It is an approximation of the absolute rate of oxidation at a given thymine, linearly normalized such that PRI = 1 describes a thymine that is fully single-stranded. Thus, a thymine with PRI = 0.4 is estimated to have been oxidized twice as fast as a thymine with PRI = 0.2.
3. ‘Fraction oxidized in A/T tract’ (FO). This metric is a mathematical transformation/combination of the PRI of all thymines in the R-loop flank of a given DNA substrate. It is an approximation of the total fraction of DNA molecules (the two strands of DNA forming the R-loop flank are referred to here as a single ‘molecule’) that, at the moment of quenching, have been oxidized on at least one of the nine thymines within the R-loop flank.

While visually inspecting phosphorimages from permanganate experiments, note that there are occasionally faint bands corresponding to strand cleavage at cytosines (permanganate oxidizes cytosines, albeit much more slowly than thymines) and at purines (which occurs during hydroxide treatment in the 3′-radiolabeling protocol). Such bands constituted a trivial fraction of the total lane volume and did not meaningfully affect analysis.

Additionally, visual inspection of the raw phosphorimages can be informative but should be approached with caution because the absolute volume of a given band is meaningless without considering other bands that may have detracted from its signal. For example, a strongly oxidized thymine (thymine 1) may yield only a faint band if another strongly oxidized thymine (thymine 2) lies between thymine 1 and the radiolabeled terminus of the DNA oligonucleotide. If all thymine oxidation events are independent of each other (i.e., thymine 1 has the same oxidation probability irrespective of whether thymine 2, or any other thymine, has been oxidized or not), the oxidation probability of thymine 1 can be reconstructed by considering the thymine-1 band only as a subpopulation of the bands above it on the gel. In other words, out of all the DNA molecules on the gel for which oxidation of thymine 1 would have been observable (i.e., cleavage fragments *at or above* the thymine-1 fragment on the gel), what fraction of those molecules *were* in fact oxidized at thymine 1? In reality, clusters of thymines have been observed to mutually enhance oxidation probability (Nomura and Okamoto, 2008), so perfect independence *cannot* be assumed. Thus, the parameters described below are imperfect measures of the true rate of oxidation at each thymine.

Beyond uncertainty in the measurement, it is also unknown to what extent the probing technique is changing the fundamental biophysical features of the DNA structures. Notably, thymine’s reaction with permanganate breaks the planarity of the nucleobase and, consequently, its capacity to stack normally. In an A/T-rich sequence like our R-loop flank (Figure 2B), an oxidation event at thymine 1 could, in principle, begin a chain reaction of oxidation events as each adjacent thymine successively loses planarity and unstacks, exposing its neighbor to the oxidant. If such chain reactions occurred quickly as compared to the timescale of the assay (2 min), the distribution of band volumes would be skewed toward thymine 9. In reality, the band volume distributions are skewed sharply toward thymine 1 (Figure 2—figure supplement 3), suggesting that, on the assayed timescale, the majority of oxidation events do *not* lead to additional oxidation events. Still, the possibility of chain reactions should be kept in mind when interpreting the observed permanganate reactivity patterns, in which reactivity decreases with distance from the R-loop edge (Figure 2B). While these patterns are consistent with fraying duplex termini, the apparent ‘depth’ of the fraying events should be interpreted as an upper limit on what would occur in a substrate unexposed to permanganate.

Finally, the structural determinants of permanganate reactivity should be considered carefully when using these data to draw conclusions about DNA conformation. While high permanganate reactivity is often associated with ‘single-strandedness’ or ‘lack of base pairing,’ the reaction is more precisely dependent upon the ability of a permanganate molecule to approach the C5=C6 bond of the thymine nucleobase. This approach could be facilitated by assumption of a non-B-form helical geometry, global melting of the DNA duplex, or ‘flipping’ of a thymine out of the duplex without dramatically affecting the helical geometry (Bui et al., 2003). Furthermore, a thymine lying on a duplex terminus could, in principle, be approached and attacked while base paired, albeit from a restricted angle. This possibility is especially important to consider for thymine 1 of our A/T-rich R-loop flank (Figure 2B). The reactivity of this thymine varies in RNA-free DNA bubble controls that have different bubble sequences (Figure 4—figure supplement 3), perhaps reflecting differences in the propensities of individual (unpaired) neighboring bases to stack on the duplex-terminal thymine. Finally, because thymines within the RNA:DNA hybrid of R-loop structures have two possible base pairing partners (DNA versus crRNA), the conformational ensemble at these positions is highly complex, and we did not attempt to draw any structural conclusions from their oxidation rates.

Given the aforementioned caveats, the PRI and FO metrics described below should be interpreted as estimates rather than accurate measurements of rate and extent of reaction. Additionally, permanganate reactivity data should be considered alongside the orthogonal techniques used in this work to assess the structure, energetics, and conformational dynamics of interhelical junctions. The definitions of permanganate reactivity index (PRI) and fraction oxidized (FO) are as follows:

Let $v_{i}$ denote the volume of band i in a lane with n total bands (band 1 is the shortest cleavage fragment, band n is the topmost band corresponding to the starting/uncleaved DNA oligonucleotide). The probability of oxidation at thymine i is defined as:

$$p_{i}=\frac{v_{i}}{\sum_{j=i}^{n}v_{j}}$$

Note that this relationship allows determination of $p_{i}$ even if the values of $p_{1\leq x<i}$ are unavailable (e.g., if the shortest cleavage products have been run off the bottom of the gel). Assuming thymine oxidation occurs with a uniform probability across the time course of permanganate application (see exponential curve in Figure 2—figure supplement 3) the rate constant associated with oxidation probability $p_{i}$ is defined as:

$$k_{i}=\frac{\ln\left(\frac{1}{1-p_{i}}\right)}{t}$$

where t is the time of quenching. We found that across experimental replicates there was systematic variation in k (e.g., k was universally smaller in replicate 2 than in replicate 1 for any given thymine), likely due to variability in the oxidation activity of each new preparation of the potassium permanganate solution. To allow comparison across replicates, we normalized all values of k to that of a reference thymine ($k_{ref}$) whose conformational dynamics were not expected to be affected by R-loop formation or associated substrate variations (the thymine 10 nt from the end of the 5′-radiolabeled oligo, present in all DNA substrates tested). For every set of replicate experiments, which each involved a new preparation of potassium permanganate solution, we determined the average rate constant of the reference thymine across all substrates (${\overset{-}{k}}_{ref}$). The global average of $k_{ref}$ across all experiments and all replicates ($\mu_{ref}$) was taken to be the true value of $k_{ref}$. The corrected value of $k_{i}$ for each thymine was then taken to be:

$$k_{i,corr}=\frac{\mu_{ref}}{{\overset{-}{k}}_{ref}}k_{i}$$

The permanganate reactivity index was then calculated as:

$${PRI}_{i}=\frac{k_{i,corr}}{k_{ss,corr}}$$

where $k_{ss,corr}$ is the reference-corrected oxidation rate constant for a thymine unassociated with a stable base-pairing partner. The value of $k_{ss,corr}$ used in our calculations was 0.79 min^−1^, empirically determined for an arbitrarily chosen thymine within a DNA bubble (Figure 2—figure supplement 3).The estimated fraction of DNA molecules oxidized on at least one thymine within the R-loop flank (correcting to the value expected if the potassium permanganate solution had its average oxidation activity) was then calculated as:

$$p_{i,corr}=1-e^{-k_{i,corr}t}$$

$$FO=1-\prod_{a\in RLF}\left(1-p_{a,corr}\right)=1-\exp\left[-\left(\sum_{a\in RLF}k_{a,corr}\right)t\right]$$

where RLF denotes the set of band indices corresponding to the thymines labeled T_1_ through T_9_ in Figure 2B, combining data from both the NTS-radiolabeled and TS-radiolabeled experiments. Note that the PRI metric is subject to increased uncertainty as $p_{i}$ approaches 1, where the slope of $\ln\left(\frac{1}{1-p_{i}}\right)$ approaches infinity.

## Figures and Tables

**Appendix 1—figure 1.**
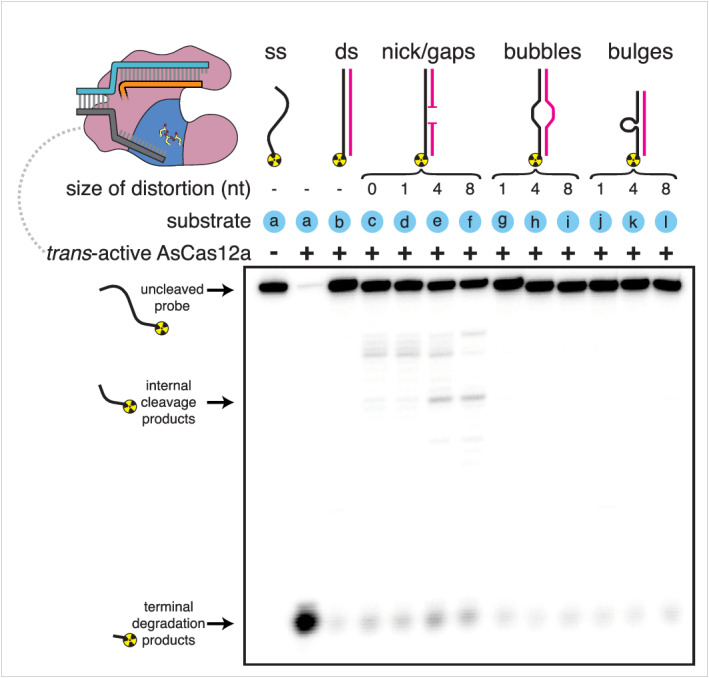
Substrate specificity of Cas12a *trans*-active holoenzyme. Phosphorimage of AsCas12a cleavage products, resolved by denaturing PAGE. *Trans*-active AsCas12a holoenzyme (115 nM of each component: protein, crRNA, pre-cleaved activator) was incubated with 1 nM of the indicated substrate for 2 hours at 30°C prior to quenching. Substrate (a) was a single-stranded DNA oligonucleotide with no homology to the crRNA. To generate substrates (b) through (l), substrate (a) was hybridized to a variety of unlabeled complementary DNA oligonucleotides. Substrate (c) contained a nick. Substrates (d), (e), and (f) contained gaps of 1, 4, and 8 nt, respectively. Substrates (g), (h), and (i) contained bubbles of 1, 4, and 8 nt, respectively. Substrates (j), (k), and (l) contained bulges of 1, 4, and 8 nt, respectively. Appendix 1—figure 1—source data 1.Numerical data plotted in Appendix 1—figure 1—figure supplement 1.

**Appendix 1—figure 1—figure supplement 1.**
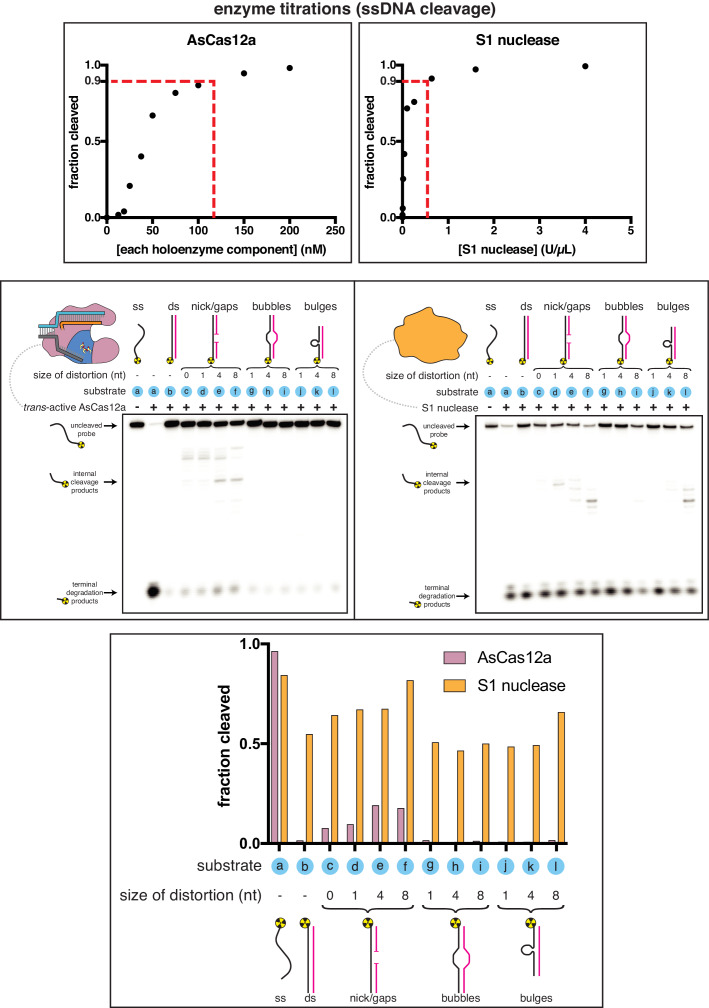
Comparing the substrate specificities of Cas12a *trans*-active holoenzyme and S1 nuclease. *Top panel*: Enzymes were first titrated in an activity assay with a radiolabeled ssDNA substrate to determine what concentration to use in substrate specificity assays. A ssDNA oligo (the same one shown in the other panels) was 5'-radiolabeled and incubated with varying concentrations of either AsCas12a/crRNA/pre-cleaved DNA activator (all components held equimolar at the indicated concentration) or S1 nuclease for 30 minutes at 30°C, followed by quenching. Products were resolved by denaturing PAGE and quantified from the phosphorimage. “U” on the x-axis of S1 nuclease refers to the units defined by the enzyme manufacturer. To achieve 90% cleavage of the ssDNA substrate in the given time course, 115 nM AsCas12a holoenzyme or 0.513 U/µL S1 nuclease was required. *Middle panels*: Phosphorimage of AsCas12a or S1 nuclease cleavage products, resolved by denaturing PAGE. *Trans*-active AsCas12a holoenzyme (115 nM of each component: protein, crRNA, pre-cleaved activator) or S1 nuclease (0.513 U/µL) was incubated with 1 nM of the indicated substrate for 2 hours at 30°C prior to quenching. Substrate (a) was a single-stranded DNA oligonucleotide with no homology to the crRNA. To generate substrates (b) through (l), substrate (a) was hybridized to a variety of unlabeled complementary DNA oligonucleotides. Substrate (c) contained a nick. Substrates (d), (e), and (f) contained gaps of 1, 4, and 8 nt, respectively. Substrates (g), (h), and (i) contained bubbles of 1, 4, and 8 nt, respectively. Substrates (j), (k), and (l) contained bulges of 1, 4, and 8 nt, respectively. *Bottom panel*: Quantifications of cleavage from phosphorimages. “Fraction cleaved” is defined as (sum of the volume of all bands below the uncleaved band)/(total volume in lane).

**Appendix 2—figure 1.**
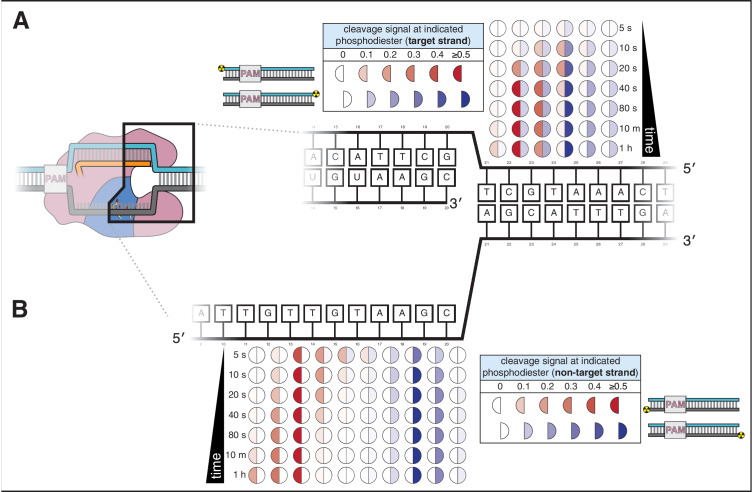
Cas12a forms a gap in the non-target strand and cleaves the target strand outside the R-loop. (**A**) Target-strand cleavage products over time, as quantified by denaturing PAGE. 100 nM AsCas12a and 120 nM crRNA were incubated with 5 nM radiolabeled DNA target at 37°C for the indicated timepoints, followed by quenching and resolution by denaturing PAGE. Representative phosphorimages are shown in Appendix 2—figure 1—figure supplement 1. Data shown here are the average of three replicates. Each circle denotes a phosphodiester at which cleavage was observed. The intensity of color in each half-circle (‘cleavage signal’) reflects the fraction (band volume for a given cleavage product) / (total volume in lane). The left half of each circle (red) corresponds to the cleavage product detected with a PAM-proximal radiolabel. The right half of each circle (blue) corresponds to the cleavage product detected with a PAM-distal radiolabel. (**B**) Non-target-strand cleavage products over time, as quantified by denaturing PAGE (phosphorimage in Appendix 2—figure 1—figure supplement 1). Data representation as in **A**. Appendix 2—figure 1—source data 1.Numerical data plotted in Appendix 2—figure 1 and accompanying figure supplements.

**Appendix 2—figure 1—figure supplement 1.**
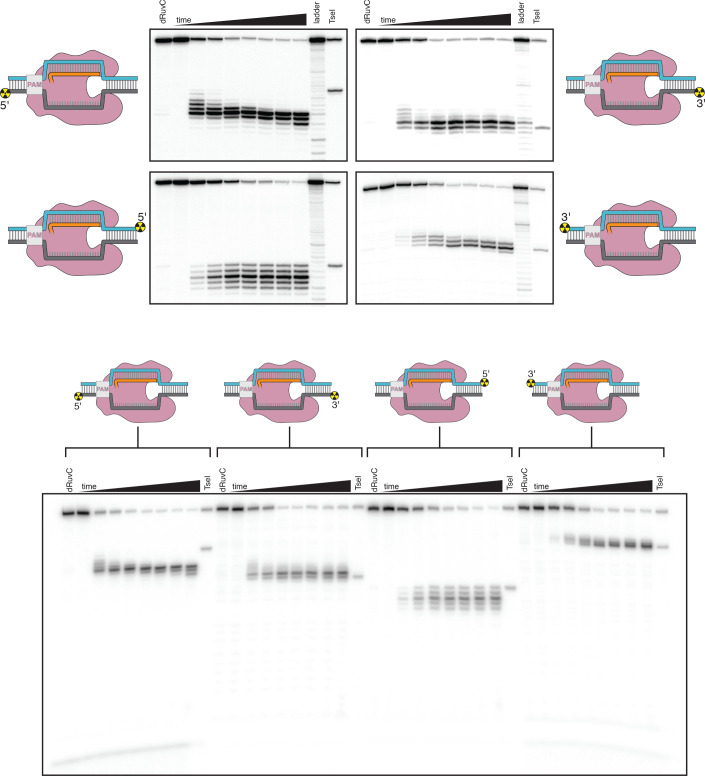
Denaturing PAGE analysis of AsCas12a cleavage products. *Top panel*: The leftmost lane in each gel is a 1-hr timepoint with AsCas12a D908A (mutation in the RuvC active site). The timepoints shown for the reactions with WT enzyme are 0 s, 5 s, 10 s, 20 s, 40 s, 80 s, 10 min, 1 hr. The ladder was made by digestion of the relevant substrate with a cocktail of non-specific DNases (see Materials and methods). The rightmost lane is the same probe cleaved by the site-specific restriction enzyme TseI. *Bottom panel*: The same products run on a gel for a shorter amount of time to demonstrate the absence of faster-migrating species. This gel lacks the ladder samples.

**Appendix 2—figure 1—figure supplement 2.**
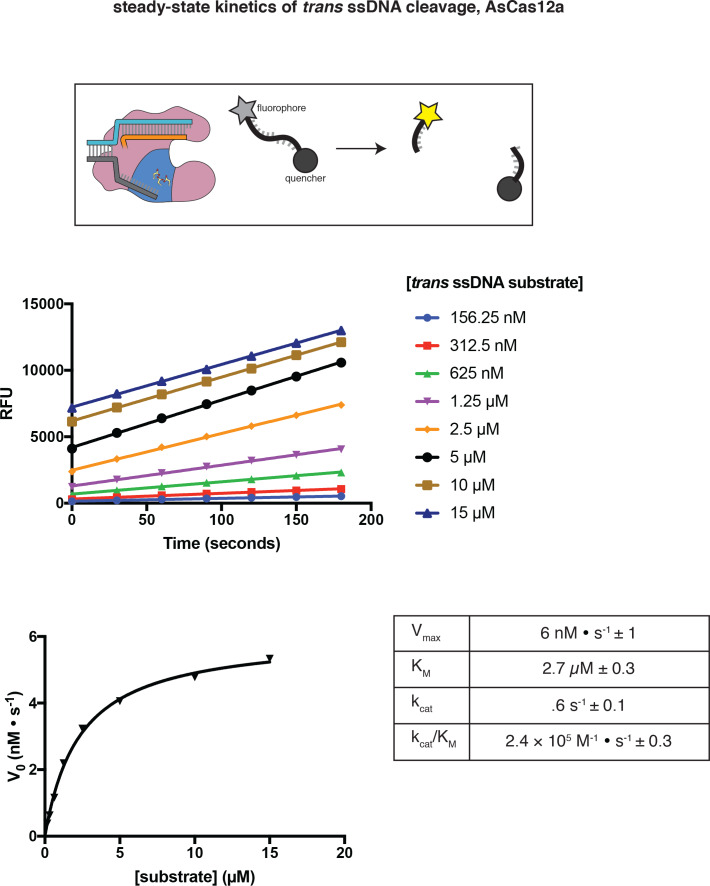
Steady-state kinetic analysis of AsCas12a *trans* DNase activity, as measured by fluorophore dequenching. Raw data of a representative replicate are shown in the middle panel. The V_0_ values of the representative replicate are plotted in the bottom panel, along with a hyperbolic fit (y = v_max_*x/(K_M_+x)). The table shows the average and standard deviation of kinetic parameters, determined from three independent replicates. The k_cat_ value reported in the table is based on an upper limit of 10 nM *trans*-active AsCas12a holoenzyme. See the figure legend to Appendix 2—figure 1—figure supplement 3 for a comparison of *trans* and *cis* cleavage kinetics.

**Appendix 2—figure 1—figure supplement 3.**
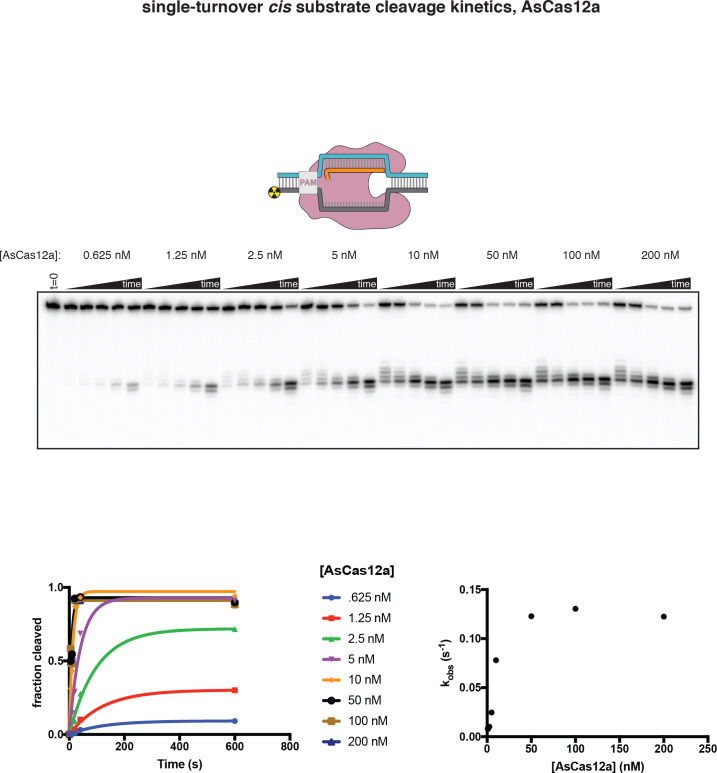
Enzyme-concentration dependence of AsCas12a *cis* DNA cleavage kinetics. 250 pM of a *cis* duplex DNA target, radiolabeled on the 5' end of the NTS, was incubated with 240 nM crRNA and various concentrations of AsCas12a at 37°C for the following timepoints: 0 s, 5 s, 10 s, 20 s, 40 s, 10 m. ‘Fraction cleaved’ is defined as (sum of the volume of all bands below the uncleaved band)/(total volume in lane). Data were fit to an exponential decay (y = (y_0_-plateau)*exp(-k*x)+plateau), with y_0_ constrained to 0. At the lower values of [AsCas12a], an early plateau indicates an underestimation of the true enzyme concentration, due to loss of enzyme across extensive serial dilution. Despite this experimental deficiency, these data show that enzyme-substrate association is not rate-limiting when [AsCas12a] is 100 nM—that is, when [AsCas12a] is 100 nM, association can be approximated as instantaneous, and cleavage rate constants reflect unimolecular processes that follow binding. Based on these experiments, initial *cis* cleavage events occur at ~0.12 s^−1^ at the reagent concentrations used for cleavage mapping experiments. Considering the steady-state kinetic measurements in Appendix 2—figure 1—figure supplement 2, an upper limit on *trans* cleavage rate can be calculated as (k_cat_/K_M_)*[E], where [E] has an approximate upper limit of 10 nM (for radiolabeled-NTS experiments, in which cold TS is present at 10 nM, and assuming ssDNA-activated complexes have similar catalytic efficiency to dsDNA-activated complexes), yielding a rate of ~0.002 s^−1^ (>50 fold less than the *cis* cleavage rate). This *trans* cleavage rate estimate is an upper limit because radiolabeled DNA is mostly duplex and likely protected by the protein in non-duplex regions (Swarts et al., 2017). Thus, *trans* cleavage probably only occurs to a detectable extent on PAM-distal DNA fragments that are released from the enzyme following *cis* cleavage.

**Appendix 2—figure 1—figure supplement 4.**
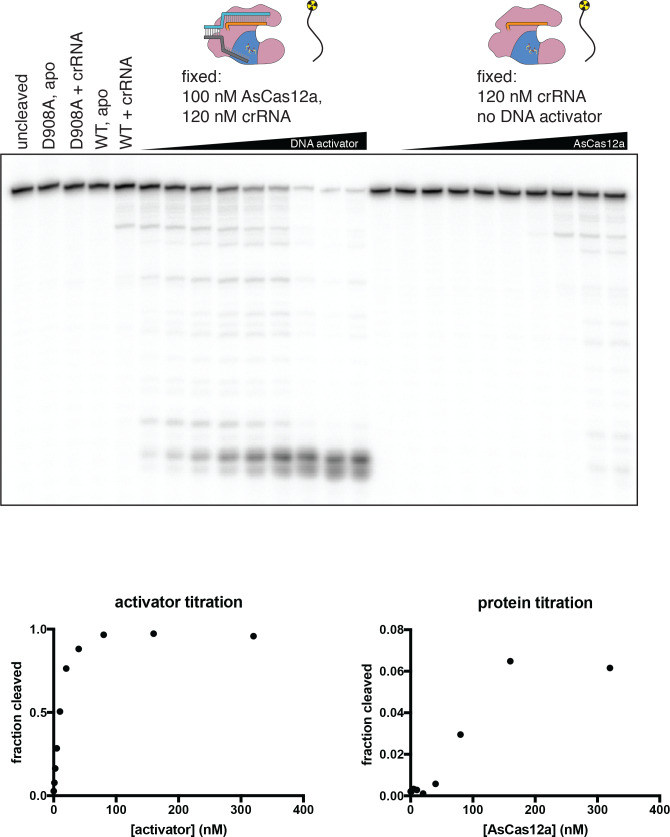
Concentration dependence of various modes of DNase activity. In biochemical reactions containing DNA, crRNA, and AsCas12a, a cut in a DNA molecule could be attributed to one of three distinct modes of RuvC DNase activity: *cis* cleavage of an enzyme’s own bound R-loop; *trans* cleavage (by an R-loop-activated complex) of free DNA or DNA in a different complex; or *trans* cleavage by an excess of DNA-free Cas12a/crRNA complex (which is shown in this figure to be catalytically competent, albeit inefficient). Various concentrations of crRNA, AsCas12a, and DNA activator were incubated with 2 nM of a radiolabeled single-stranded DNA oligonucleotide for 1 hr at 37°C prior to quenching and resolution by denaturing PAGE. ‘Fraction cleaved’ is defined as (sum of the volume of all bands below the uncleaved band)/(total volume in lane). We show here that both DNA-activated and DNA-free modes of *trans* cleavage are dependent upon enzyme concentration in the nanomolar range—that is, unlike *cis* cleavage, which occurs with saturated binding kinetics at the concentrations used (see Appendix 2—figure 1—figure supplement 3), *trans* cleavage remains concentration-dependent in this range. Therefore, DNase cleavage products that appear with kinetics that are independent of enzyme concentration can be uniquely attributed to PAM-dependent *cis* cleavage (see Appendix 2—figure 1—figure supplement 5).

**Appendix 2—figure 1—figure supplement 5.**
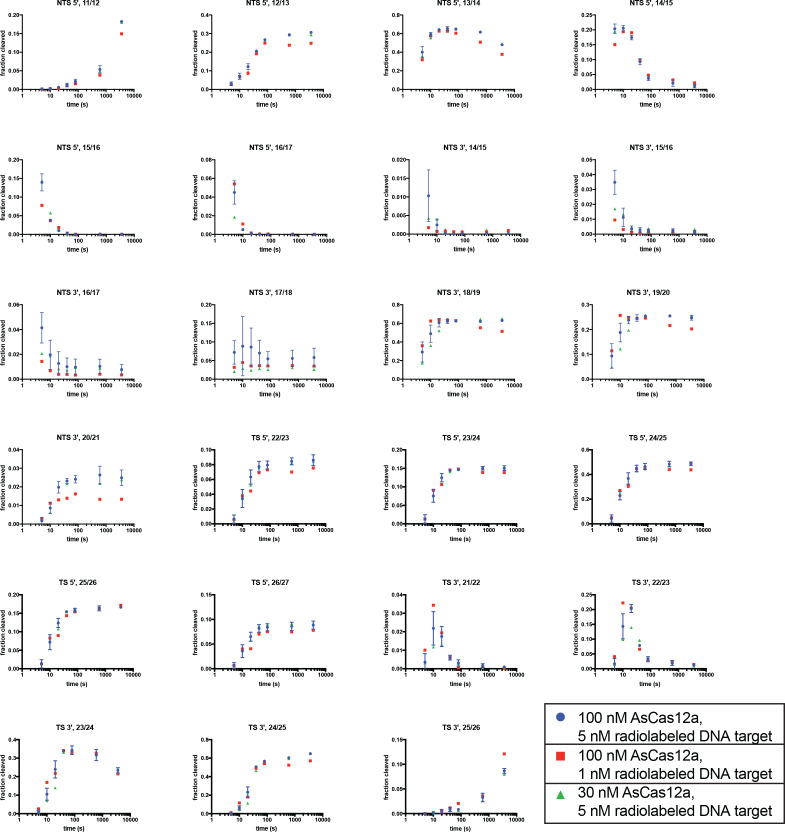
Concentration dependence of phosphodiester-mapped cleavage events. Each graph follows the kinetics of appearance/disappearance of a cleavage product at the indicated phosphodiester. ‘Fraction cleaved’ is defined as (band volume for a given cleavage product)/(total volume in lane). For example, ‘NTS 5', 11/12’ displays the fraction of 5'-radiolabeled NTS that was detected to have been cleaved at the 11/12 dinucleotide for each timepoint. Blue circles are equivalent to the data shown in Appendix 2—figure 1, with error bars denoting standard deviation across three replicates. Red squares and green triangles indicate variants of this experiment in which the total concentration of either DNA-bound holoenzyme (red squares) or AsCas12a/crRNA (green triangles) was decreased. The fact that almost all species exhibit equivalent kinetics of appearance/disappearance in all three conditions (with the potential exception of the phosphodiesters on the portion of the PAM-distal NTS fragment that remains single-stranded after release by the enzyme, which exhibit slight concentration-dependence on longer timescales) indicates that we are observing mostly *cis* cleavage events, as explained in Appendix 2—figure 1—figure supplement 4.

**Appendix 2—figure 1—figure supplement 6.**
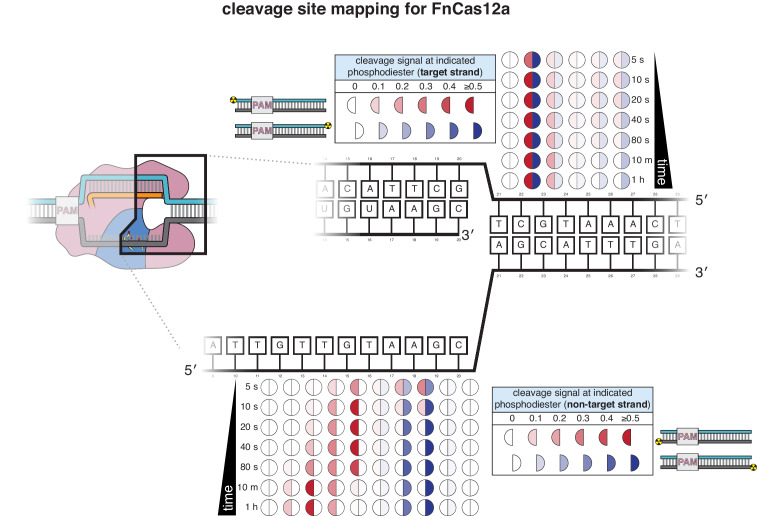
Cleavage product mapping for FnCas12a. The evolution of the pattern of *cis* cleavage over time for FnCas12a was assayed analogously to that of AsCas12a, as in Appendix 2—figure 1. The pattern of cleavage is largely similar to that of AsCas12a. However, the NTS gap of FnCas12a widens more gradually than that of AsCas12a, and the TS cut-site distribution is more tightly constrained to a single phosphodiester. No DNA cleavage was detected on either strand when a RuvC-inactivated FnCas12a mutant (D917A) was used.

**Appendix 2—figure 2.**
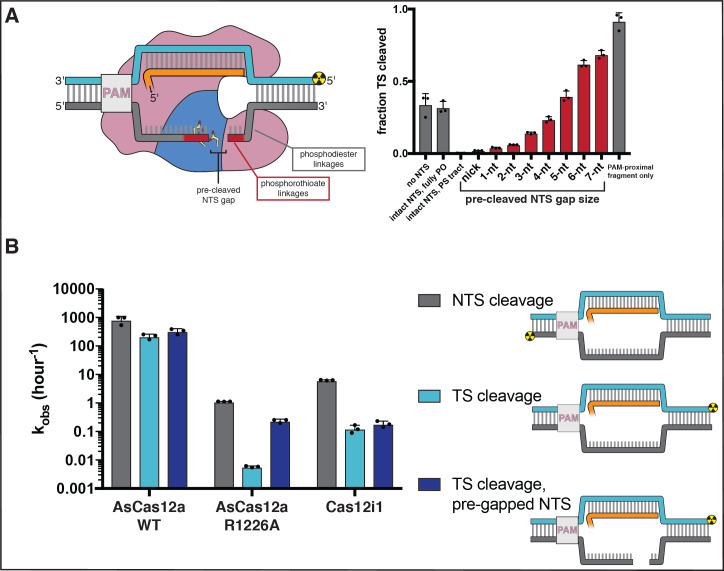
Non-target-strand gap formation poses a kinetic barrier to target-strand cleavage for AsCas12a. (**A**) Extent of target-strand cleavage by wild type AsCas12a in the presence of various non-target-strand variants, as resolved by denaturing PAGE (phosphorimage in Appendix 2—figure 2—figure supplement 5). Cas12a surveillance complex (100 nM AsCas12a, 120 nM crRNA) was added to 1 nM pre-hybridized target DNA radiolabeled on the 5' end of the TS and allowed to incubate in cleavage buffer with 5 mM CaCl_2_ for 1 hr at 37°C prior to quenching. In the schematic, the red portion of the NTS denotes phosphorothioate (PS) linkages; the gray portion denotes phosphodiester (PO) linkages. In the graph, red bars denote reactions with a PS-containing NTS variant; gray bars denote reactions either with no NTS or with an NTS variant containing only PO-linkages. From left to right (omitting the no-NTS control), the NTS variants used were A, B, D, G, J, N, Q, T, W, Y, Z, as schematized in Appendix 2—figure 2—figure supplement 4. Columns and associated error bars indicate the mean and standard deviation of three replicates. (**B**) Cleavage kinetics of NTS, TS, and TS complexed with a pre-gapped NTS (NTS contains a 5-nt gap). 100 nM protein and 120 nM cognate crRNA were incubated with 2 nM DNA target with a 5' radiolabel on the indicated strand at 37°C for various timepoints, followed by quenching and resolution by denaturing PAGE. Representative phosphorimages and quantifications are shown in Appendix 2—figure 2—figure supplement 8. Columns and associated error bars indicate the mean and standard deviation of three replicates. Appendix 2—figure 2—source data 1.Numerical data plotted in Appendix 2—figure 2 and accompanying figure supplements.

**Appendix 2—figure 2—figure supplement 1.**
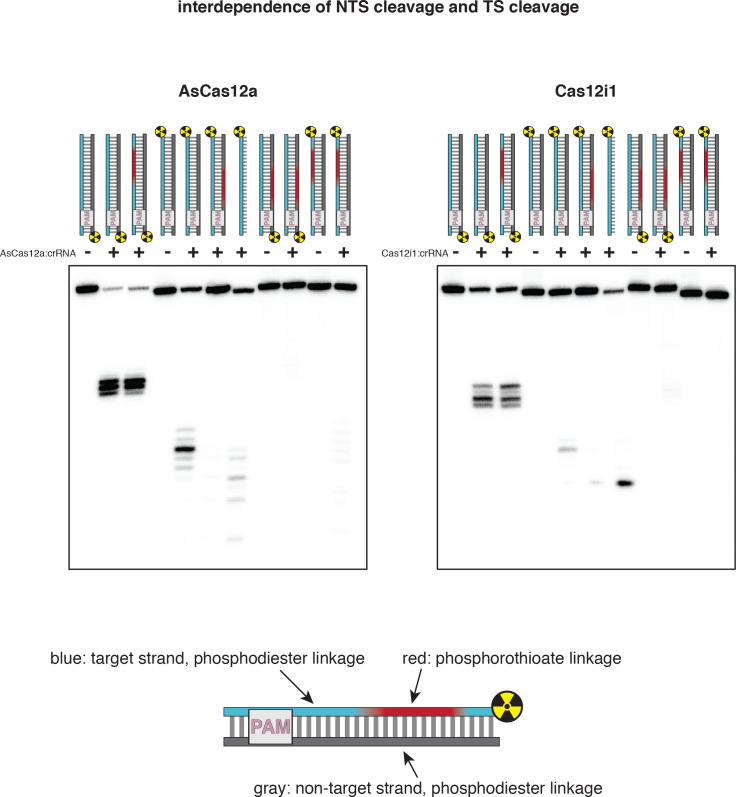
Non-target-strand cleavage precedes target-strand cleavage for AsCas12a and Cas12i1. 100 nM AsCas12a or Cas12i1 was incubated with 120 nM cognate crRNA and 2 nM radiolabeled duplex DNA target for 1 hr at 37°C, followed by quenching, denaturing PAGE, and phosphorimaging. For AsCas12a, the reaction was conducted in 5 mM CaCl_2_. For Cas12i1, the reaction was conducted in 5 mM MgCl_2_. In the duplex diagrams, red shading indicates the presence of a phosphorothioate (PS) tract across the standard cleavage sites. Blue indicates phosphodiester (PO) linkages within the TS. Gray indicates phosphodiester linkages within the NTS. For both AsCas12a and Cas12i1, the nature of the linkages in the TS has no apparent effect on NTS cleavage. However, the presence of phosphorothioates in the NTS inhibits cleavage of both the NTS and the TS. Trace TS cleavage is observed for Cas12i1 in the PS-NTS condition (lane 6)—it is unclear whether this is due to TS cleavage prior to NTS cleavage or to incomplete duplex formation, as the trace cleavage event is shifted to the site cleaved during ssDNA-targeting (lane 7).

**Appendix 2—figure 2—figure supplement 2.**
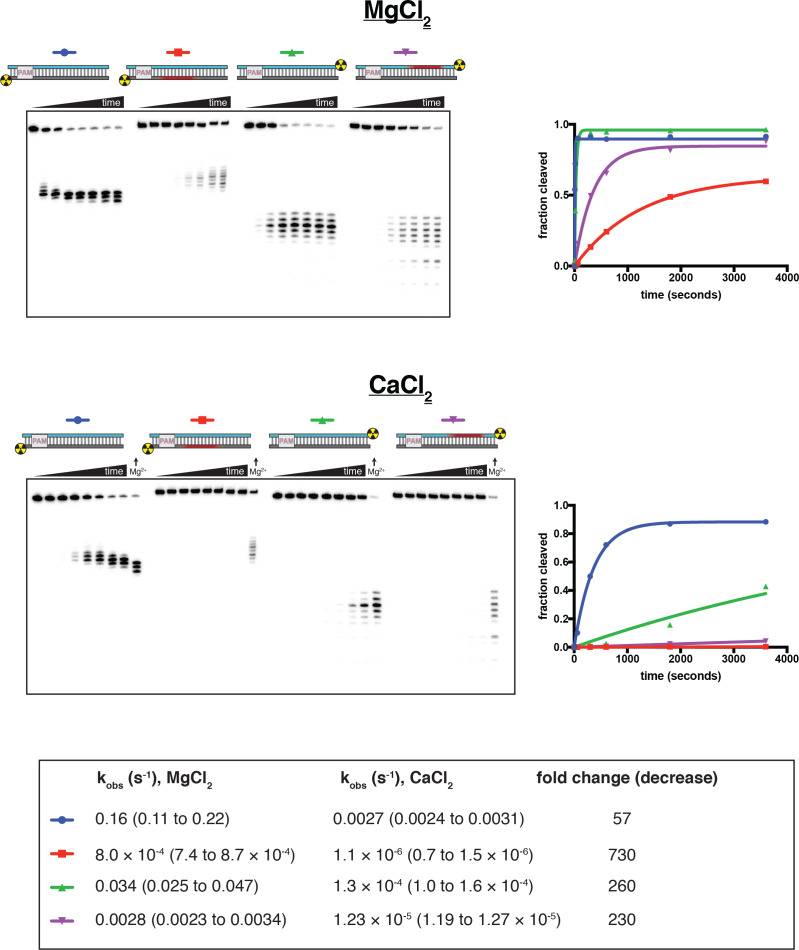
Cleavage at phosphorothioates can be selectively slowed by substitution of CaCl_2_ for MgCl_2_. 100 nM AsCas12a and 120 nM crRNA were incubated with 1 nM radiolabeled duplex DNA target at 37°C, followed by quenching (at timepoints 0, 5 s, 15 s, 1 min, 5 min, 10 min, 30 min, 1 hr) and resolution by denaturing PAGE. Substrate diagrams are colored as in Appendix 2—figure 2—figure supplement 1. The top panel shows the experiment done in cleavage buffer with 5 mM MgCl_2_. The bottom panel shows the experiment done in cleavage buffer with 5 mM CaCl_2_—at the end of each time course on this gel, the 1-hr timepoint of the MgCl_2_ experiment is included for visual comparison. ‘Fraction cleaved’ is defined as (sum of the volume of all bands below the uncleaved band)/(total volume in lane). Data were fit to an exponential decay (y = (y_0_-plateau)*exp(-k*x)+plateau), with y_0_ constrained to 0. The plateau value was constrained to 1 for those time courses that did not exceed fraction cleaved = 0.5 by the 1-hr timepoint. Rate constants (with 95% confidence intervals) are shown in the table below the gels. It is unclear why cleavage of a phosphorothioated TS occurs more rapidly than cleavage of a phosphorothioated NTS, although it is conceivable that this is an intrinsic feature of the enzyme cleavage pathway when the chemical transformation is rate-limiting. Considering only effects on the NTS, the calcium substitution decreases the phosphodiester cleavage rate by a factor of 57 and decreases the phosphorothioate cleavage rate by a factor of 730, resulting in a 13-fold increase in selectivity for phosphodiesters over phosphorothioates and yielding kinetics slow enough to resolve by manual pipetting.

**Appendix 2—figure 2—figure supplement 3.**
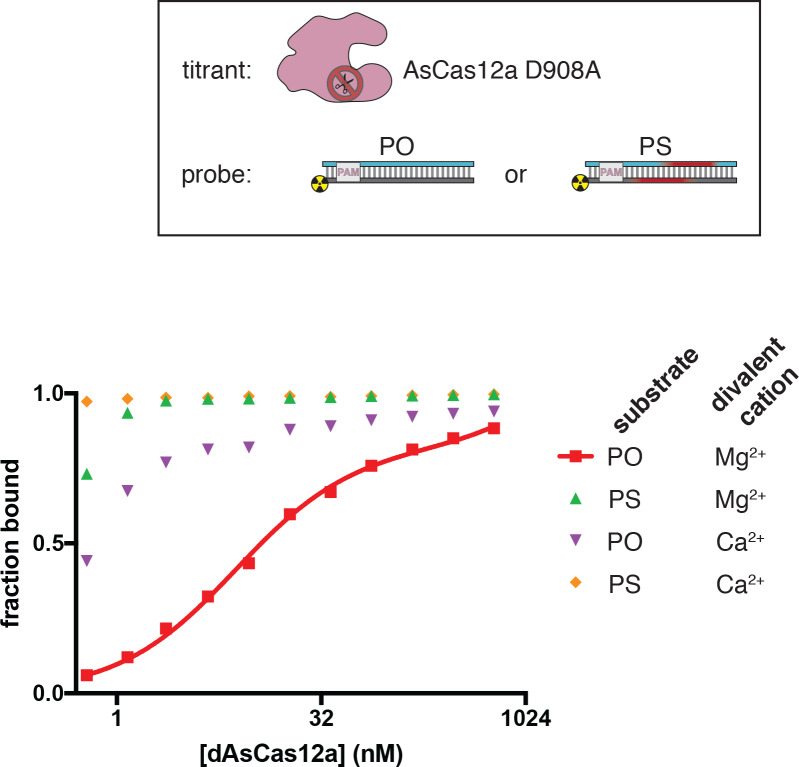
Interference complexes are stable in the presence of CaCl_2_ and with a phosphorothioated DNA target. Using a filter-binding assay, we assessed the affinity of dAsCas12a/crRNA for cognate DNA targets, either fully phosphodiester (PO) or containing phosphorothioate (PS) linkages across the NTS and TS cleavage sites, in the presence of either 5 mM MgCl_2_ or 5 mM CaCl_2_. Substrate diagrams are colored as in Appendix 2—figure 2—figure supplement 1. ‘Fraction bound’ is defined as (background-subtracted volume of Protran spot)/(total background-subtracted volume of Protran spot + Hybond N+ spot). The value of ‘fraction bound’ was 0 at [dAsCas12a]=0 for both substrates and both assays (not shown due to the logarithmic x-axis). All data shown are from a representative replicate (n = 3). When appropriate, data were fit to the sum of a hyperbola and a line (y = B_max_*x/(K_D_+x)+NS*x), where NS describes a non-specific binding mode. It is common to see B_max_ values below 1 in filter binding assays, in which the process of physical separation can disrupt bound species. K_D_ for the PO substrate in MgCl_2_ was 8.1 nM ±0.8 (SD) (n = 3). Data from the other binding conditions indicated that the K_D_ was near or below [DNA probe], preventing accurate K_D_ determination by hyperbolic fitting. For unknown reasons, the Mg^2+^→Ca^2+^ and the PO→PS substitutions stabilized the ribonucleoprotein:DNA interaction both separately and together.

**Appendix 2—figure 2—figure supplement 4.**
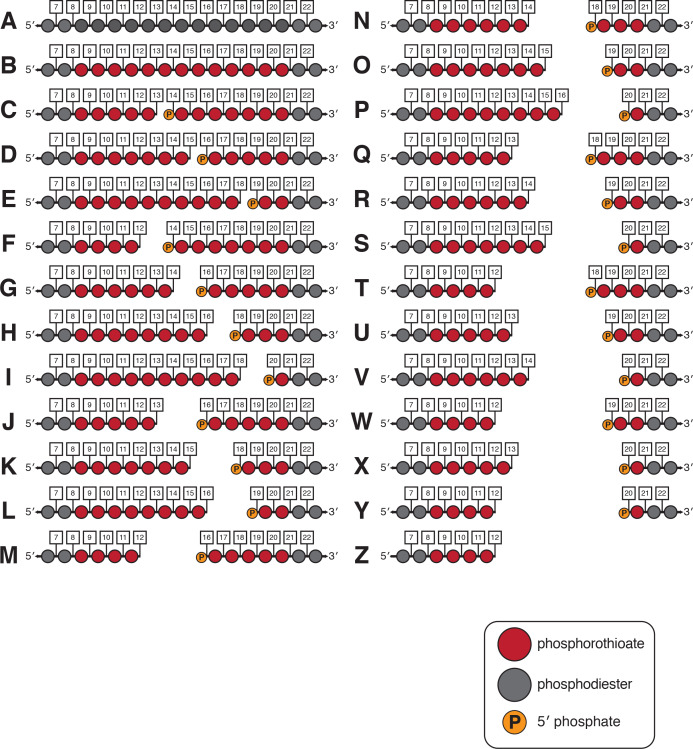
Non-target-strand variants used in gap-dependence experiments. Nucleotides 7–22 of the NTS are shown for each variant. A red circle indicates a phosphorothioate linkage. A gray circle indicates a phosphodiester linkage. A 5′ phosphate was placed on all PAM-distal NTS fragments to recapitulate the end chemistry left by the RuvC-catalyzed hydrolysis reaction.

**Appendix 2—figure 2—figure supplement 5.**
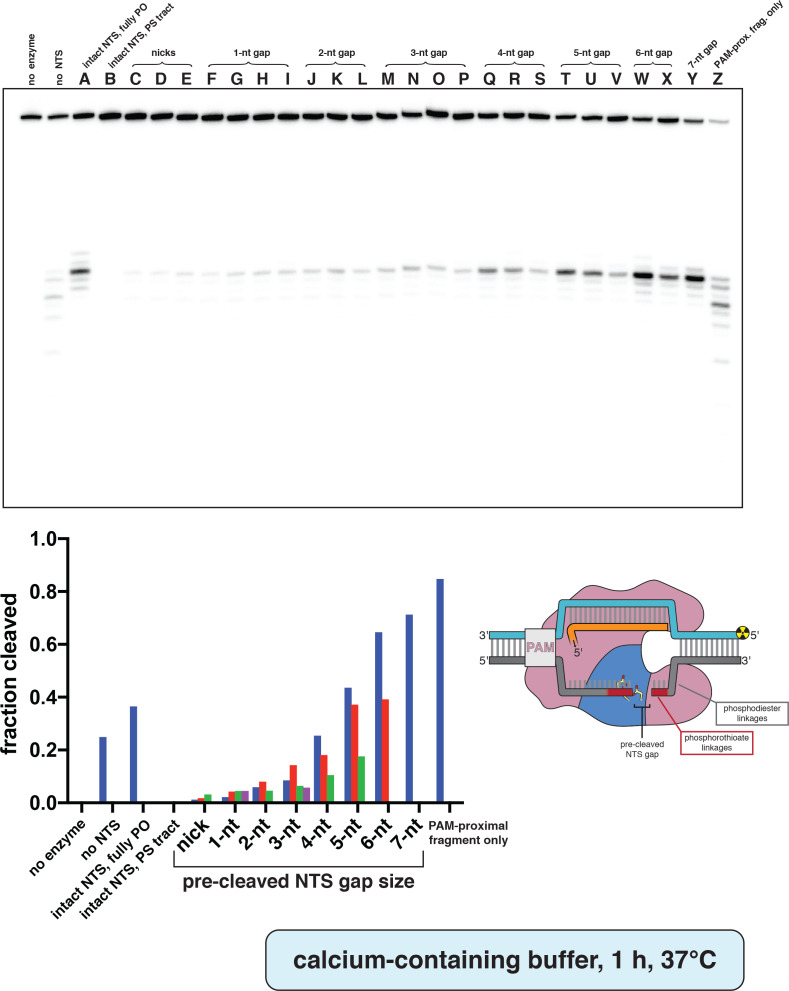
Phosphorimage and quantification of non-target-strand gap-dependence experiments, in CaCl_2_. Experiment was performed as described in legend to Appendix 2—figure 2A. Substrates indicated by a letter are as schematized in Appendix 2—figure 2—figure supplement 4. When a given category has more than one substrate (e.g., 3-nt gap includes substrates M, N, O, and P), the first listed substrate (M) is shown as a blue bar, the second (N) as a red bar, the third (O) as a green bar, and the fourth (P) as a pink bar. ‘Fraction cleaved’ is defined as (sum of the volume of all bands below the uncleaved band)/(total volume in lane).

**Appendix 2—figure 2—figure supplement 6.**
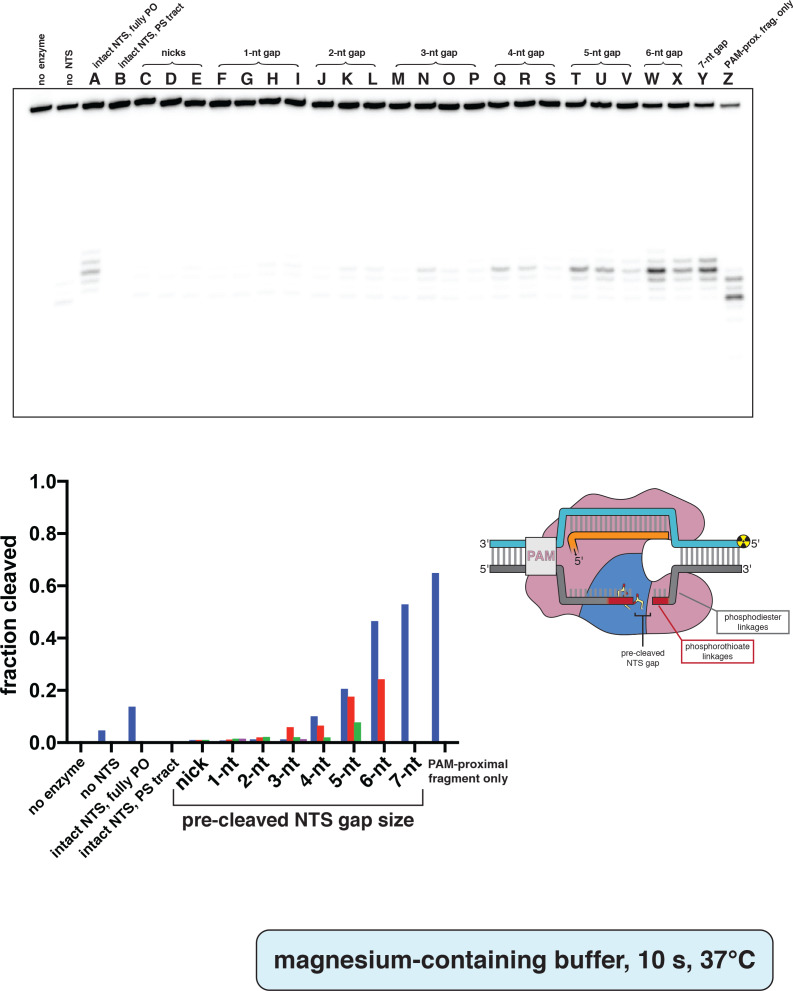
Phosphorimage and quantification of non-target-strand gap-dependence experiments, in MgCl_2_. Experiment was performed as described in legend to Appendix 2—figure 2A, except 5 mM MgCl_2_ was used instead of CaCl_2_, and the reaction was quenched after only 10 s at 37°C. Substrates indicated by a letter are as schematized in Appendix 2—figure 2—figure supplement 4. When a given category has more than one substrate (e.g., 3-nt gap includes substrates M, N, O, and P), the first listed substrate (M) is shown as a blue bar, the second (N) as a red bar, the third (O) as a green bar, and the fourth (P) as a pink bar. ‘Fraction cleaved’ is defined as (sum of the volume of all bands below the uncleaved band)/(total volume in lane).

**Appendix 2—figure 2—figure supplement 7.**
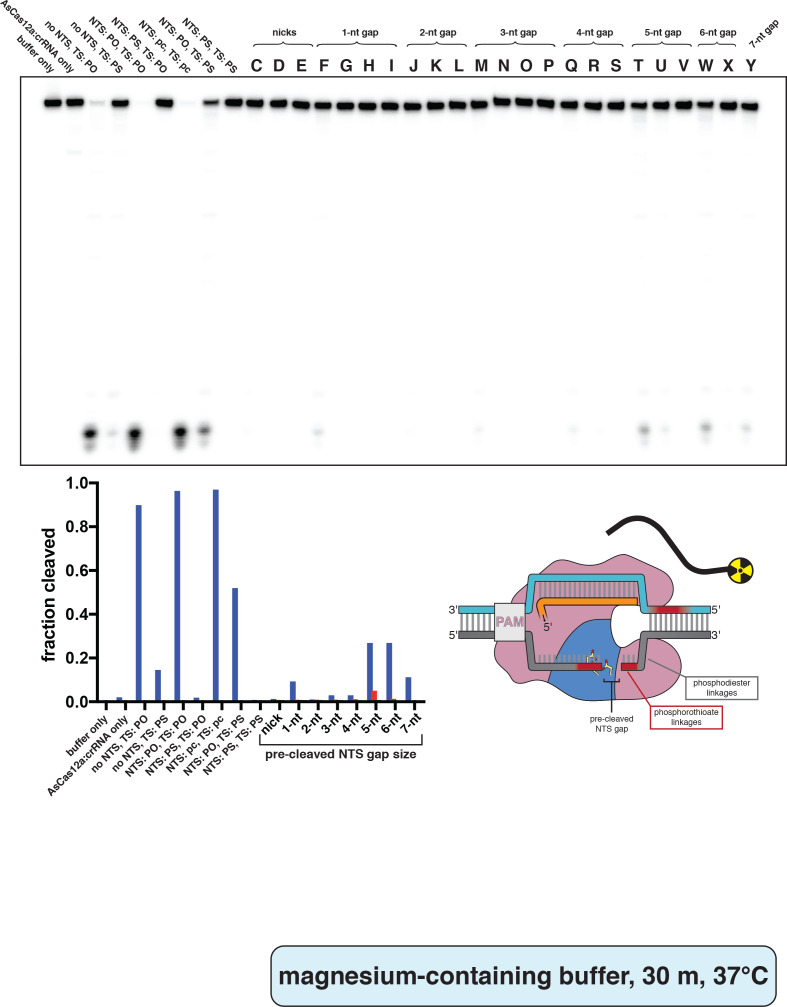
Phosphorimage and quantification of non-target-strand gap-dependence experiments, in MgCl_2_, with radiolabeled *trans* substrate. Extent of *trans* cleavage by wild type AsCas12a in the presence of various DNA activator variants, as resolved by denaturing PAGE. Cas12a ternary complex (final concentrations: 100 nM AsCas12a, 120 nM crRNA, pre-hybridized 120 nM TS/240 nM NTS) was assembled with each of the indicated NTS variants and combined with 2 nM (final concentration) radiolabeled non-specific *trans* ssDNA target in cleavage buffer (5 mM MgCl_2_). These reactions were then incubated for 30 min at 37°C prior to quenching and resolution by denaturing PAGE. Control lanes on the left contain some combination of intact NTS/TS with phosphodiesters (PO) or phosphorothioates (PS) across the standard cleavage sites; ‘pc’ stands for pre-cleaved (only PAM-proximal cleavage products: NTS truncated after nt 13, TS truncated after nt 22). Reactions without NTS contained 120 nM of a non-specific DNA oligonucleotide to account for substrate competition. All lanes indicated by a letter (C–Y) contained an NTS variant (see Appendix 2—figure 2—figure supplement 4) along with a TS containing phosphorothioates across the standard cleavage sites. When a given category has more than one substrate (e.g., 3-nt gap includes substrates M, N, O, and P), the first listed substrate (M) is shown as a blue bar, the second (N) as a red bar, the third (O) as a green bar, and the fourth (P) as a pink bar. ‘Fraction cleaved’ is defined as (sum of the volume of all bands below the uncleaved band)/(total volume in lane).

**Appendix 2—figure 2—figure supplement 8.**
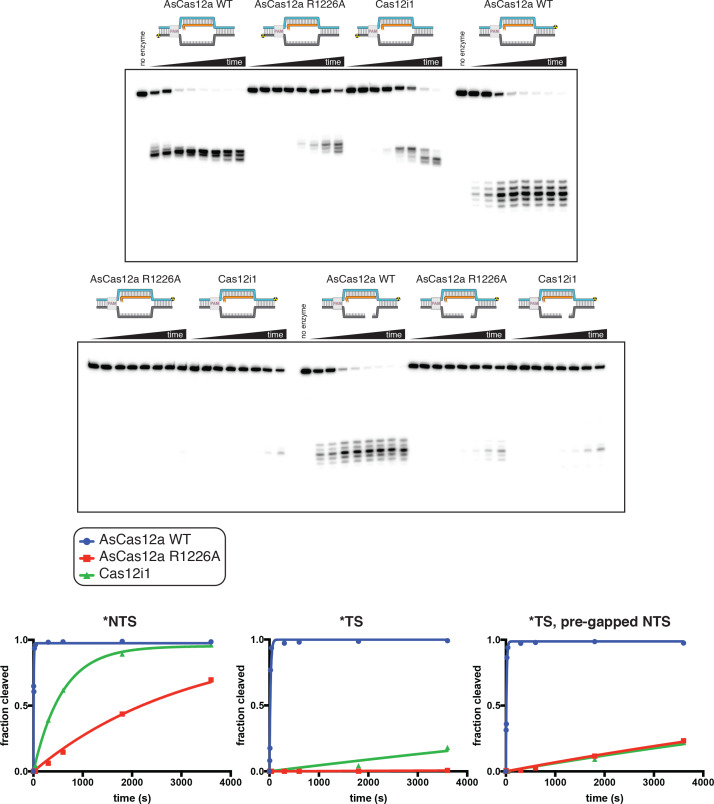
Phosphorimages and quantification of pre-gapped non-target-strand experiments. Experiment was performed as described in legend to Appendix 2—figure 2B. ‘Fraction cleaved’ is defined as (sum of the volume of all bands below the uncleaved band)/(total volume in lane). Data were fit to an exponential decay (y = (y_0_-plateau)*exp(-k*x)+plateau), with y_0_ constrained to 0. The plateau value was constrained to 1 for those time courses that did not exceed fraction cleaved = 0.5 by the 1-hr timepoint. The exponential decay constant k is reported as k_obs_ in Appendix 2—figure 2B. The data shown here are from a representative replicate (n = 3).

**Appendix 2—figure 2—figure supplement 9.**
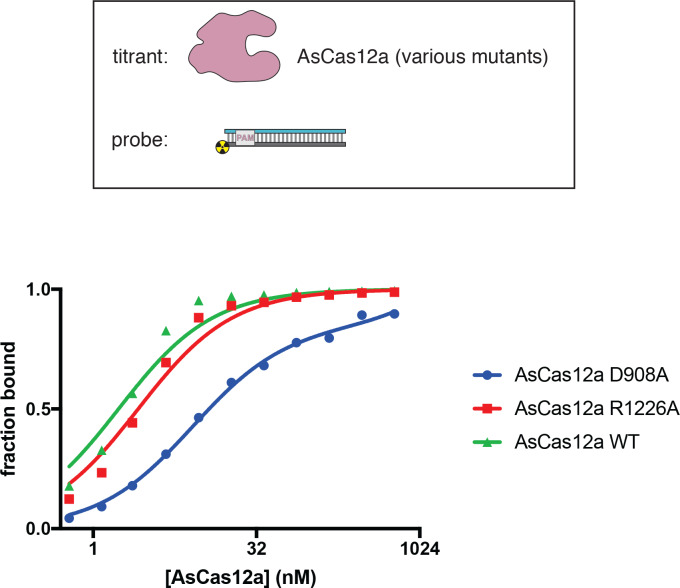
Affinity measurements for RNA-guided interaction of AsCas12a mutants with dsDNA. The affinity of AsCas12a/crRNA for a cognate DNA target was assessed by a filter-binding assay, with three variants of AsCas12a. AsCas12a was titrated in a solution with fixed [crRNA] (750 nM) and [DNA probe] (100 pM), in binding buffer containing MgCl_2_, followed by separation of protein-bound DNA from free DNA on membranes. ‘Fraction bound’ is defined as (volume of Protran spot)/(total volume of Protran spot + Hybond N+ spot). The value of ‘fraction bound’ was 0 at [AsCas12a]=0 for all protein variants (not shown due to the logarithmic x-axis). All data shown are from a representative replicate (n = 3). Data were fit to the sum of a hyperbola and a line (y = B_max_*x/(K_D_+x)+NS*x), where NS describes a non-specific binding mode. It is common to see B_max_ values below 1 in filter-binding assays, in which the process of physical separation can disrupt bound species. K_D_ for AsCas12a D908A was 11 nM ±3 (SD) (n = 3). K_D_ for AsCas12a R1226A was 2.7 nM ±0.2 (SD) (n = 3). K_D_ for AsCas12a WT was 1.86 nM ±0.04 (SD) (n = 3).

**Appendix 2—figure 2—figure supplement 10.**
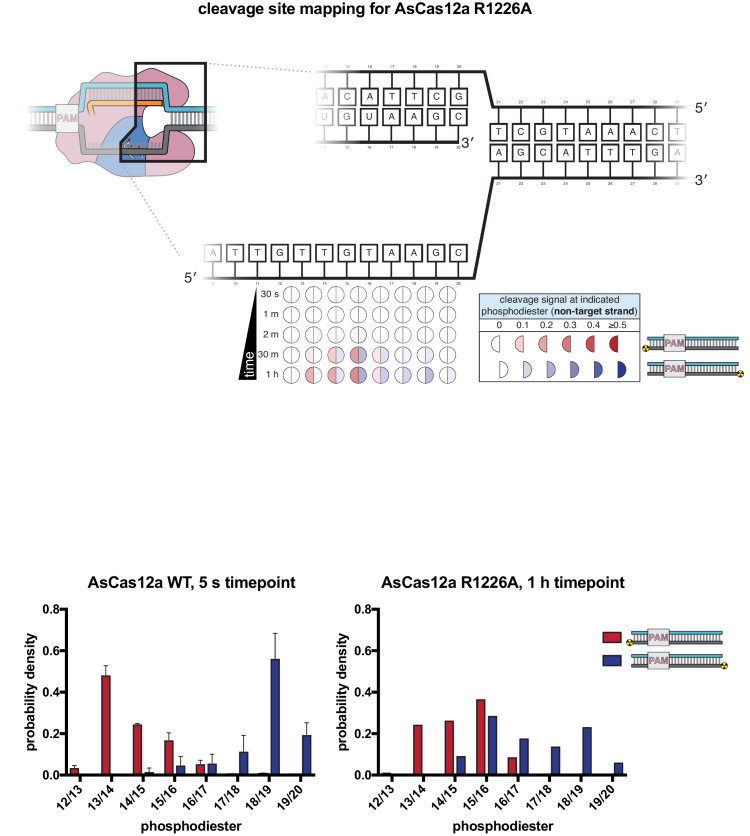
Non-target-strand cleavage product mapping for AsCas12a R1226A. The evolution of the pattern of *cis* cleavage over time for AsCas12a R1226A was assayed analogously to that of WT AsCas12a, as in Appendix 2—figure 1. At the 1-hr timepoint, AsCas12a R1226A had made at least one cut in ~70% of the assayed NTS molecules, which matches the average fraction cleaved by WT AsCas12a at the 5-s timepoint. The cleavage site distributions in each of these experiments can be compared by calculating the probability density of each cleavage product as: (volume of band corresponding to cleavage at phosphodiester X/Y)/(total volume of all cleaved products), such that the area under each distribution is 1. Unlike the WT distribution, for which the 5′-mapped and 3′-mapped distributions are mostly non-overlapping, the R1226A distributions have significant overlap at dinucleotides 14/15, 15/16, and 16/17. In our ensemble biochemical experiment, this overlap is consistent with (although not uniquely explainable by) the presence of a large population of molecules that have been cleaved exactly once (i.e., have not undergone gap formation). If this explanation is correct, these data point to an accumulation of AsCas12a R1226a complexes with a once-cut NTS, supporting the model that gap formation is disproportionately slow (and perhaps rate-limiting for TS cleavage) for this mutant.

**Appendix 2—figure 2—figure supplement 11.**
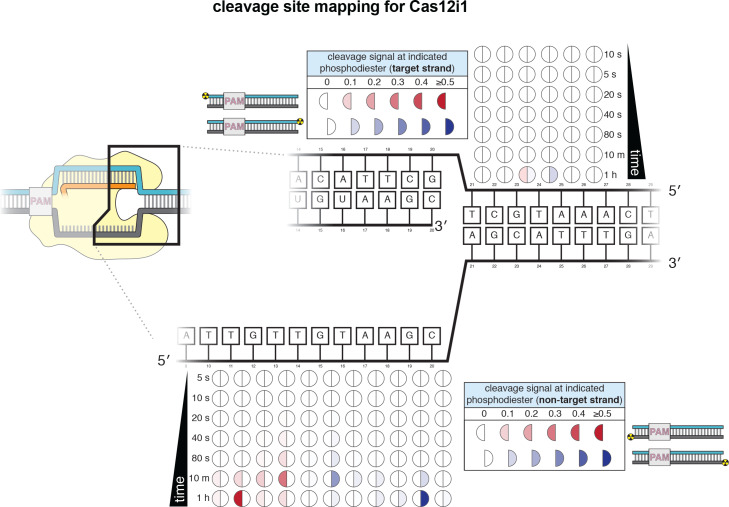
Cleavage product mapping for Cas12i1. The evolution of the pattern of *cis* cleavage over time for Cas12i1 was assayed analogously to that of WT AsCas12a, as in Appendix 2—figure 1. The pattern of cleavage is largely similar to that of AsCas12a, although cleavage kinetics are dramatically slower, and disproportionately so for TS cleavage. The NTS gap of Cas12i1 is wider (8 nt) than that of AsCas12a (5 nt) at 1 hr. No DNA cleavage was detected on either strand when a RuvC-inactivated Cas12i1 mutant (D647A) was used.

## Data Availability

All data generated or analyzed during this study are included in the manuscript and supporting files, with the exception of raw phosphorimages (which are difficult to interpret without the authors' guidance because of complex and arbitrary gel-loading schemes) and fully sampled molecular dynamics trajectories (which have a file size of ~2.2 TB). Representative images and trajectories have been included in the manuscript, supplementary figures, and/or source data. The original files are available from the authors upon request.

## Additional files

Supplementary file 1. Sequences of plasmids, DNA oligonucleotides, and RNA oligonucleotides used in this work.

Transparent reporting form

## Appendix 1

The RuvC active site of Cas12a selectively cuts single-stranded DNA While the ssDNA specificity of Cas12a RuvC *trans* activity has been previously reported (Chen et al., 2018), we wanted to probe the strength and mechanism of this specificity, which could lend insight into the enzyme-substrate conformations that permit *cis* cleavage. For example, does *trans*-active Cas12a only cut DNA substrates that are completely single-stranded, or will it also cut small distortions in dsDNA, as has been observed for S1 nuclease, an unrelated DNase with reported specificity for single-stranded substrates (Wiegand et al., 1975)? To compare the substrate range of Cas12a RuvC to that of S1 nuclease, we tested the susceptibility of various radiolabeled DNA structures (including a single strand, a duplex, a nicked duplex, and duplexes with gaps, bubbles, and bulges) to cleavage by the two enzymes, used at concentrations with comparable activity. In contrast to S1 nuclease, which exhibited minimal discrimination against even the fully duplex substrate, Cas12a discriminated strongly against substrates with up to 8-nt tracts of non-duplex DNA. This stringent substrate preference suggests that non-ssDNA structures are either sterically excluded from the RuvC active site or unable to assume catalytic geometry once bound. However, strand discontinuities as small as a nick were sufficient to permit low levels of internal strand cleavage by Cas12a (Appendix 1—figure 1, Appendix 1—figure 1—figure supplement 1). Notably, the nicked structure resembles the juxtaposed duplexes of an R-loop boundary, and the increased sensitivity of this substrate to *trans* cleavage may emerge from the same phenomenon that enables *cis* cleavage of the target strand.

## Appendix 2

Non-target-strand gap formation is required for efficient cleavage of the target strand Elucidating the mechanism of target-strand cleavage requires an understanding of its interplay with non-target-strand cleavage. To precisely determine the location of Cas12a-catalyzed NTS cleavage, we monitored the formation of DNA cleavage products over time by denaturing PAGE, and we distinguished between different DNA fragments by placing radiolabels on the 5′ or 3′ end of each strand (Appendix 2—figure 1, Appendix 2—figure 1—figure supplement 1, Figure 2—figure supplement 1). These experiments were conducted on a timescale and at an enzyme concentration for which *cis* cleavage events (i.e., events in which a Cas12a molecule cuts the DNA molecule to whose PAM it is bound) are the primary contributor to the observed DNA cutting signal (Appendix 2—figure 1—figure supplements 2, 3). Additionally, while *trans* cleavage events (i.e., events in which a Cas12a molecule cuts free DNA or DNA bound to another Cas12a molecule) may minorly contribute, the concentration-dependence of the *trans* cleavage mode allows it to be distinguished from *cis* cleavage processes (Appendix 2—figure 1—figure supplements 4, 5). According to these mapping experiments, the non-target strand has two major cleavage sites, at dinucleotides 13/14 and 18/19 (numbers denote distance from the PAM), suggesting the formation of a 5-nt gap within the tract of DNA displaced by the crRNA (Appendix 2—figure 1B). The evolution of the cleavage pattern over time indicates that, in at least some fraction of the molecules assayed, the NTS is first cut between the two major sites and achieves its final state through two or more ‘trimming’ events. A Cas12a ortholog from *Francisella novicida* also produced a gap in the NTS, implying that this phenomenon may be conserved across type V-A enzymes (Appendix 2—figure 1—figure supplement 6). Because our ensemble biochemical assay is blind to the occurrence of additional cuts that occur farther from the radiolabel than the first cut, we cannot unambiguously assign cleavage states to individual interference complex molecules. Nevertheless, by comparing the 5′- and 3′-mapped cut-site distributions at a given timepoint, we can roughly assess the predominant cleavage state of individual molecules. For example, a population of DNA molecules cut exactly once would yield completely overlapping 5′-/3′-mapped cut-site distributions, while a population with a gap would yield non-overlapping peaks in the two distributions. Therefore, in the target-strand mapping experiments, overlap of the 5′- and 3′-mapped distributions is consistent with (although not uniquely explainable by) a population of interference complexes that have cleaved the TS exactly once (Appendix 2—figure 1A). However, separation of the two distribution peaks (at dinucleotides 22/23 and 24/25) indicates that most individual complexes perform at least one additional cut in the TS, yielding a small TS gap prior to dissociation of the PAM-distal cleavage product (Appendix 2—figure 1A; Singh et al., 2018). These results show that while Cas12a activity does generate staggered cuts in dsDNA targets as previously reported (Zetsche et al., 2015), its trimming activity, most notably on the NTS, destroys the capacity of released DNA products to be trivially re-ligated in a restriction-enzyme-like procedure. In this particular DNA target, the major Cas12a cleavage products contain a 9-nt overhang on the PAM-proximal fragment and a 6-nt overhang on the PAM-distal fragment, with 4 nt of complementarity between these overhangs (Appendix 2—figure 1). These findings may aid in the study of eukaryotic DNA repair pathways elicited by Cas12a cleavage events and in biotechnological applications of Cas12a that exploit its staggered cuts. For Cas12a, non-target-strand cleavage is a prerequisite of target-strand cleavage (Swarts and Jinek, 2019; Appendix 2—figure 2—figure supplement 1). To test whether gap formation in the NTS is also an obligatory step of Cas12a-catalyzed DNA cleavage, we modified the NTS with phosphorothioates to selectively inhibit cleavage of certain DNA linkages. Substitution of calcium for magnesium in the reaction buffer produced a 13-fold increase in the RuvC DNase’s selectivity for phosphodiester over phosphorothioate linkages, allowing us to effectively halt expansion of a defined gap size in the NTS while simultaneously measuring cleavage of a fully phosphodiester-linked TS (Appendix 2—figure 2—figure supplement 2). Neither the calcium substitution nor the inclusion of phosphorothioates impaired enzyme-substrate complex assembly (Appendix 2—figure 2—figure supplement 3). Using a series of NTS variants that were chemically locked in various states of cleavage (i.e., intact, a single nick, and gaps of varying sizes) (Appendix 2—figure 2—figure supplement 4), we measured the extent of TS cleavage after one hour in calcium-containing buffer. TS cleavage was almost undetectable in the presence of a single nick, and its extent of cleavage only reached that observed with a fully phosphodiester-linked NTS when the gap was widened to 5 nt (Appendix 2—figure 2A, Appendix 2—figure 2—figure supplement 5). We observed a similar trend when the experiment was conducted in the presence of magnesium (Appendix 2—figure 2—figure supplement 6). Together, these results indicate that formation of a gap in the NTS accelerates TS cleavage. Although NTS gap formation is not strictly required for TS cleavage to occur, our bulk cleavage analysis suggests that the NTS gap does in fact form before TS cleavage in the native Cas12a cleavage pathway (Appendix 2—figure 1). Thus, for most experiments in this work that probed the mechanism of TS cleavage (Figures 2–5), we used substrates that recapitulated the 5-nt NTS gap (referred to as a ‘pre-cleaved’ or ‘pre-gapped’ NTS). The dependence of TS cleavage on NTS gap formation suggests that the NTS occludes the RuvC active site immediately following R-loop formation. After a NTS nicking event, RuvC releases and rebinds the NTS in different registers to cleave it in multiple locations, forming a gap that clears the active site for entry of the TS. Consistent with a substrate-occlusion model, we determined that *trans* ssDNA cleavage is enhanced by gap formation in the NTS and only achieves its maximal rate when the TS has also been cut (Appendix 2—figure 2—figure supplement 7). These observations hint at a possible evolutionary origin for non-specific *trans* activity—an enzyme that must loosely shuttle multiple *cis* substrates into and out of a shared catalytic center would benefit from a promiscuous and ‘open’ active site. Therefore, the target-activated non-specific ssDNase activity of type V Cas enzymes may be a mechanistic artifact of single-DNase *cis* cleavage rather than a direct immunological necessity. To further understand the interplay between non-target-strand and target-strand cleavage, we investigated strand cleavage kinetics in two type V Cas enzymes that have been reported to act as NTS ‘nickases’ (i.e., cleave the NTS but not the TS): the R1226A mutant of AsCas12a (R1226 lies adjacent to the RuvC active site in the tertiary protein structure) (Yamano et al., 2016) and the type V-I interference enzyme Cas12i1 (Yan et al., 2019). With our sensitive phosphorimaging assay, we determined that these enzymes do in fact cleave the TS, albeit slowly, suggesting that the cleavage signal simply did not rise above the detection limit in previous experiments (Yamano et al., 2016; Yan et al., 2019; Appendix 2—figure 2B, Appendix 2—figure 2—figure supplement 8). Importantly, slow TS cleavage activity was coupled to slow NTS cleavage activity for both AsCas12a R1226A and Cas12i1 (Appendix 2—figure 2B, Appendix 2—figure 2—figure supplement 8), suggesting that slow TS cleavage emerges mostly from low overall catalytic efficiency. While this low efficiency could be explained by weak target association for Cas12i1, which exhibited no detectable DNA binding activity in our filter-binding assay, the affinity of AsCas12a R1226A for DNA was unimpaired as compared to WT AsCas12a (Appendix 2—figure 2—figure supplement 9). Still, both enzymes exhibited k_TS_:k_NTS_ ratios lower than that of WT AsCas12a, suggesting that there may also be more fundamental differences in their DNA cleavage pathways (Appendix 2—figure 2B). Specifically, we wondered whether these enzymes were able to form non-target-strand gaps. To test this question, we began by performing cleavage site mapping on the NTS of AsCas12a R1226A. At the 1-hr timepoint, the 5′- and 3′-mapped cut-site distributions contained significant overlap, suggestive of a population of DNA strands containing either a single nick or a small gap (Appendix 2—figure 2—figure supplement 10). In contrast, at the 5-s timepoint of the NTS mapping experiments for WT AsCas12a, the two distributions were almost completely non-overlapping, having already developed peaks at the major cut sites (Appendix 2—figure 1B, Appendix 2—figure 2—figure supplement 10). Because the 1-hr timepoint for AsCas12a R1226A and the 5-s timepoint for WT AsCas12a have similar values (~70%) of total NTS cleavage (an unambiguous measure of the fraction of molecules that have experienced at least one cut), the difference in distributions implies a fundamental difference across the two enzymes in terms of their relative rates of NTS nicking and trimming (i.e., R1226A has a lower ratio of k_trim_:k_nick_ than WT) (Appendix 2—figure 1B, Appendix 2—figure 2—figure supplement 10). To more directly probe the kinetic contribution of NTS trimming activity, we measured the rate of AsCas12a R1226A TS cleavage in an interference complex with a pre-gapped NTS. The observed TS cleavage rate in this complex was 40-fold higher than in the one with an intact NTS (Appendix 2—figure 2B), suggesting that the physical basis for the disproportionately slow TS cleavage kinetics of AsCas12a R1226A actually lies in disproportionate slowing of a step prior to TS cleavage (i.e., NTS gap formation). Gap-widening cleavage events may be slower than the initial nicking event in this mutant due to the high entropic cost of associating a severed strand with the active site, as nicking of the NTS is expected to boost its conformational freedom (Xiao et al., 2018). Similarly to Cas12a, Cas12i1-mediated TS cleavage depends upon NTS cleavage (Appendix 2—figure 2—figure supplement 1). However, cleavage-site mapping for Cas12i1 revealed that a NTS gap had already formed at the earliest timepoints for which cleavage was detectable (Appendix 2—figure 2—figure supplement 11). Additionally, Cas12i1-mediated TS cleavage could be only slightly accelerated by pre-gapping the NTS, indicating that NTS trimming is not rate-limiting for TS cleavage in Cas12i1 (Appendix 2—figure 2B, Appendix 2—figure 2—figure supplement 8). These results suggest that across diverse families of type V interference complexes, the microscopic steps of double-strand break formation can vary in absolute rate and relative kinetic breakdown. Still, while both AsCas12a R1226A and Cas12i1 exhibit k_NTS_:k_TS_ ratios favorable for ‘nickase’ applications, their use may be limited by their low NTS cleavage rate, which is 10^2^–10^3^ times slower than that of WT AsCas12a under the tested conditions. Thus, Cas9 remains the tool of choice for generating RNA-guided nicks in either DNA strand because each of its DNase domains can be independently inactivated by point mutation (Jinek et al., 2012).
